# Chemical Constituents from the Roots and Rhizomes of *Asarum heterotropoides* var. *mandshuricum* and the In Vitro Anti-Inflammatory Activity

**DOI:** 10.3390/molecules22010125

**Published:** 2017-01-13

**Authors:** Yu Jing, Yi-Fan Zhang, Ming-Ying Shang, Guang-Xue Liu, Yao-Li Li, Xuan Wang, Shao-Qing Cai

**Affiliations:** 1 State Key Laboratory of Natural and Biomimetic Drugs, School of Pharmaceutical Sciences, Peking University Health Science Center, No. 38, Xueyuan Road, Beijing 100191, China; jing_jy@126.com (Y.J.); zhangyifan@bjmu.edu.cn (Y.-F.Z.); guangxl@bjmu.edu.cn (G.-X.L.); 13466759800@163.com (Y.-L.L.); xuanwang6818@bjmu.edu.cn (X.W.); 2 Tonghua Gold-Horse Pharmaceutical Group Co., Ltd., Beijing 100025, China

**Keywords:** *Asarum heterotropoides* var. *mandshuricum*, Aristolochiaceae, lignans, 8-*O*-4′ neolignan, amide, monoterpene, anti-inflammatory

## Abstract

Anti-inflammatory compounds were investigated from the ethanol extract of the roots and rhizomes of *Asarum heterotropoides* var. *mandshuricum*, a traditional Chinese medicine called Xixin and used for pain and inflammatory. Nine new compounds were isolated, including six new lignans, neoasarinin A–C (**1**–**3**), neoasarininoside A and B (**4** and **5**), and asarinin B (**7**), and one new monoterpene, asarincin A (**8**), two new amides, asaramid II and III (**10** and **11**), and one new natural monoterpene, asaricin B (**9**), along with 37 known compounds (**6**, **12**–**47**). Their structures and absolute configurations were elucidated on the basis of spectroscopic methods and chemical analyses. This is the first report of the absolute configuration of asarinin A (**6**). The 8-*O*-4′ neolignans (**1**–**5**) were reported in the genus *Asarum* for the first time. The 15 compounds **17**, **19**, **22**–**25**, **28**, **31**, **36**, **40**, **42**, **43**, **45**–**47** were isolated from the genus *Asarum*, and compounds **16**, **32**, **33**, **37** and **39** were isolated from *A. heterotropoides* var. *mandshuricum* for the first time. Thirty-seven of the isolates were evaluated for anti-inflammatory activity against the release of β-glucuronidase in polymorphonuclear leukocytes (PMNs) induced by the platelet-activating factor (PAF), and compounds **1**, **4**, **7**, **8**, **14**, **17**–**19**, **22**, **24**, **25**, **29**, **30**, **32**, **33**, **40**–**43**, **45**, and **46** showed potent anti-inflammatory activities in vitro, with 27.9%–72.6% inhibitions at 10^−5^ mol/L. The results of anti-inflammatory assay suggested that lignans obtained from the CHCl_3_ extract might be the main active components of Xixin.

## 1. Introduction

The roots and rhizomes of *Asarum heterotropoides* Fr. Schmidt var. *mandshuricum* (Maxim.) Kitag, which is described as Xixin in Chinese or Asari Radix et Rhizoma (AR) in the Pharmacopoeia of the People’s Republic of China (2010 edition), is one of the most important traditional Chinese medicines of the genus *Asarum* (Aristolochiaceae). It was widely used in traditional medicine as an anodyne and anti-allergic remedy to treat various diseases such as aphthous stomatitis, headache, toothache, and inflammation in some countries of Asia [1]. Previous phytochemical investigations of *A. heterotropoides* var. *mandshuricum* resulted in the identification of chemical constituents with diverse structures including volatile oil [2], lignans [3,4,5,6], amides [7], terpenes [8], flavones [6,9,10,11,12], and benzene derivatives [6,11,12,13,14]. Additionally, the volatile oil of AR was considered the main effective component contributing to analgesia and anti-inflammation [15,16]. However, when a clinical application of a water decoction of AR was used to treat pain or inflammation diseases, the decocting process lasted for a long time [17,18]. Moreover, several studies proved that the volatile oil content was very low in the water decoction boiling for a long time of AR [19,20]. Meanwhile, in the course of our studies on anti-inflammatory and anti-nociceptive effects in mice of water and ethanol extracts of AR [21], it was also found that both 95% and 50% ethanol extracts showed significantly anti-inflammatory effects in the xylene-induced mice ear edema test with inhibitory rates of 61.86% and 52.56% at 0.2 g/kg, respectively. According to the previous results, it could be concluded that non-volatile constituents existed in AR also had the anti-inflammatory and analgesic activities. However, although AR worked to control pain and inflammation in traditional Chinese medicine prescriptions, there was not enough research to explain its non-volatile active components of analgesia and anti-inflammation, in spite of a few previous papers reporting its potential anti-tussive, anti-tumor, and anti-inflammatory effects [5,6,22,23], along with anti-allergic and anti-inflammatory components of *Asiasarum sieboldi* F. Maekawa belonging to the same genus [24,25,26].

In order to investigate the anti-inflammatory constituents in the roots and rhizomes of *A. heterotropoides* var. *mandshuricum*, the present study was therefore designed to describe the extraction, isolation, structure characterization, and anti-inflammatory activity screening of non-volatile compounds isolated from ethanol extract of this plant. As a result, 47 compounds were isolated and identified, including six new lignans (**1**–**5**, **7**), one new monoterpene (**8**), two new amides (**10** and **11**), one new natural product (**9**), and 37 known compounds. The structures of new compounds were elucidated on the basis of IR, UV, MS, CD, and 1D and 2D NMR spectra. Since β-glucuronidase plays an important role in the inflammatory process, inhibitors of β-glucuronidase release may be accordingly considered therapeutic agents in inflammatory diseases [27]. Consequently, in order to evaluate the anti-inflammatory activity of non-volatile constituents, 37 compounds were screened in vitro by assessing the inhibitory rate of the release of β-glucuronidase in polymorphonuclear leukocytes (PMNs) induced by the platelet-activating factor (PAF) in this paper.

## 2. Results and Discussion

**Figure 1:**
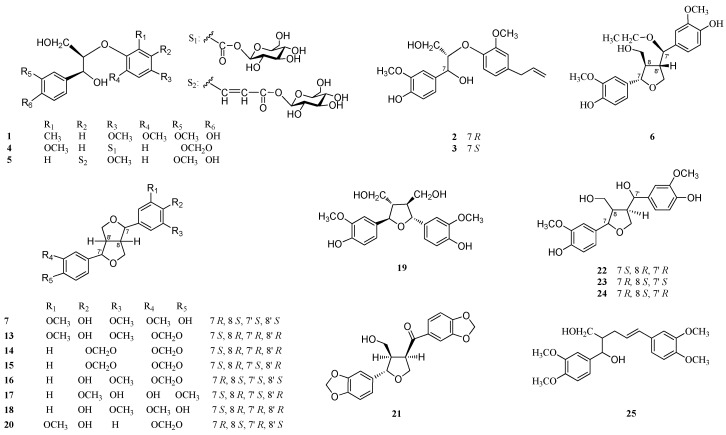
Chemical structures of compounds **1**–**7** and **13**–**25**.

**Figure 2:**
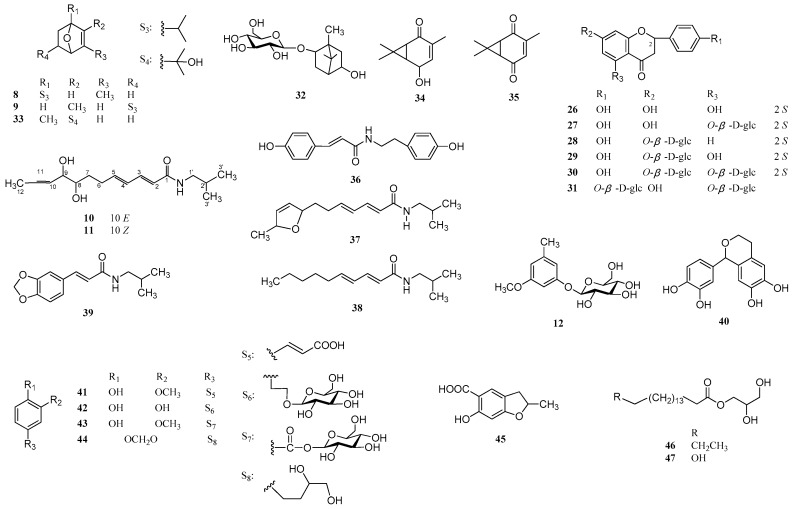
Chemical structures of compounds **8**–**12** and **26**–**47**.

The EtOH extract of the roots and rhizomes of *A. heterotropoides* var. *mandshuricum* was suspended in H_2_O and then partitioned with petroleum ether, CHCl_3_, EtOAc, and n-BuOH, successively. The CHCl_3_, EtOAc, and *n*-BuOH extracts were separated and purified by various column chromatographic methods to yield six new lignans: neoasarinin A (**1**), neoasarinin B (**2**), neoasarinin C (**3**), neoasarininoside A (**4**), neoasarininoside B (**5**), and asarinin B (**7**); one new monoterpene: asarincin A (**8**); two new amides: asaramid II (**10**) and III (**11**); and one new natural monoterpene: asaricin B (**9**), together with 37 known compounds (**6**, **12**–**47**) (Figure 1 and Figure 2). Compounds **1**, **2**, **4**–**9**, **12**–**19**, **21**–**25**, **27**–**33**, **37**, and **40**–**47** were tested for anti-inflammatory activity.

**Figure 3:**
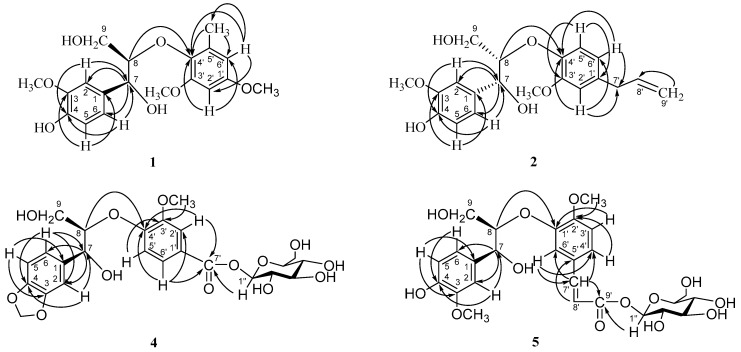
Key HMBC correlations (H → C) of compounds **1**, **2**, and **4**–**5**.

Compound **1**, an amorphous powder, was shown to have a molecular formula of C_19_H_24_O_7_ by HRMALDIMS (*m/z* 387.1403 [M + Na]^+^, calcd. 387.1414). The IR spectrum showed absorptions for hydroxyl (3445 cm^−1^). The ^1^H-NMR spectrum showed an ABX and an AX system aromatic proton signals at *δ* 6.96 (1H, d, *J* = 2.4 Hz, H-2), 6.77 (1H, dd, *J* = 8.4, 2.4 Hz, H-6), and 6.73 (1H, d, *J* = 8.4 Hz, H-5), and 6.41 (1H, d, *J* = 3.0 Hz, H-2′) and 6.29 (1H, d, *J* = 3.0 Hz, H-6′), respectively. The ^1^H-NMR signals at *δ* 3.81 (3H, s), 3.80 (3H, s), 3.71 (3H, s), and 2.12 (3H, s) supported the existence of three methoxyl groups and a methyl group. Through the analysis of HMBC spectrum, the correlations between H-2 and C-4, C-7, H-6 and C-4, C-7, H-5 and C-3, C-1, the methoxyl protons (*δ* 3.71) and C-3 indicated the presence of a 3-methoxyl-4-hydroxyphenyl unit. And the HMBC cross peaks between H-2′ and C-4′, C-6′, H-6′ and C-4′, C-2′ and methyl carbon (*δ* 15.7), methyl protons (*δ* 2.12) and C-4′, C-6′ could be attributed to a 5-methyl-1,3-dimethoxylphenyl unit (Figure 3). The other ^1^H-NMR signals, including two oxygenated methine protons (*δ* 4.87 (1H, d, *J* = 4.2 Hz, H-7) and 4.18 (1H, m, H-8)), an oxygenated methylene group (*δ* 3.83 (1H, m, H-9a) and 3.62 (1H, dd, *J* = 12.6, 3.6 Hz, H-9b)), in combination with the HMBC and ^1^H-^1^H COSY experiments, suggested that **1** could be a 8-*O*-4′ type of neolignan [28].

The coupling constant between H-7 and H-8 was measured to be 4.2 Hz, which resulted in an erythro configuration at C-7 and C-8 [29,30]. Also, the NOE cross peaks observed between H-7 and H-8 indicated the relative configuration to be *cis*. A negative Cotton effect at 219 nm suggested the absolute configuration of 7*S*, 8*R* in **1** by analysis of its CD spectrum [31,32]. From these data, the structure of **1**, therefore, was established as erythro-(7*S*, 8*R*)-7-(3-methoxyl-4-hydroxyphenyl)-8-(5′-methyl-1′,3′-dimethoxylphenoxyl) propane-7,9-diol, and it was named neoasarinin A.

The spectroscopic data of compound **1** are as follows: amorphous powder; ${\left[\alpha \right]}_{D}^{20}$ +10.0 (*c* 0.60, MeOH); UV (MeOH) *λ*_max_ (log *ε*) 231 (4.05), 280 (3.70), 308 (3.01) nm; CD (MeOH) *λ*_max_ (*c* 0.56 × 10^−4^ mol/L, Δ*ε*) 219 (–0.48), 293 (–0.24); IR (KBr) *ν*_max_ 3445, 2939, 2873, 1602, 1516, 1493, 1464, 1332, 1274, 1209, 1152, 1123, 1090, 1056, 1033, 825 cm^−1^; ^1^H-NMR (CD_3_OD, 600 MHz) and ^13^C-NMR (CD_3_OD, 150 MHz) data, see Table 1; EIMS *m/z* 364 [M]^+^ (4), 316 (11), 288 (4), 194 (32), 181 (8), 168 (100), 153 (63), 125 (25), 109(11), 93 (19), 65 (17), 53 (10); HRMALDIMS *m/z* 387.1403 [M + Na]^+^ (calcd. for C_19_H_24_O_7_Na, 387.1414).

Compound **2** was isolated as an amorphous powder. The molecular formula, C_20_H_24_O_6_, was established by HRMALDIMS (*m/z* 383.1457 [M + Na]^+^, calcd. 383.1465). The IR absorptions of **2** were indicative of the presence of a hydroxyl group (3423 cm^−1^) and a C=C double bond (1639 cm^−1^). The ^1^H-NMR signals of two oxygenated methine protons (*δ* 4.82 (1H, d, *J* = 5.4 Hz, H-7) and 4.28 (1H, m, H-8)), an oxygenated methylene group (*δ* 3.75–3.83 (2H, m, H-9)), and HMBC correlations from H-2 and H-6 to C-7, and H-8 to C-4′ (Figure 3), supported the idea that **2** could be a 8-*O*-4′ type of neolignan [28]. The ^1^H-NMR spectrum showed the presence of a 3-methoxyl-4-hydroxyphenyl unit (*δ* 7.00 (1H, d, *J* = 2.4 Hz, H-2), 6.73 (1H, d, *J* = 8.4 Hz, H-5), 6.82 (1H, dd, *J* = 8.4, 2.4 Hz, H-6) and a methoxyl group at *δ* 3.79 (3H, s)), which was supported by the HMBC spectrum. In addition, the ^1^H-NMR spectrum of **2** also showed an additional trisubstituted aromatic ring with an ABX system protons at *δ* 6.75 (1H, d, *J* = 1.8 Hz, H-2′), 6.83 (1H, d, *J* = 7.2 Hz, H-5′), 6.64 (1H, dd, *J* = 7.2, 1.8 Hz, H-6′) and a methoxyl group at *δ* 3.76 (3H, s). Furthermore, by the analysis of ^1^H-, ^13^C-NMR and ^1^H-^1^H COSY spectra, the signals at *δ* 3.28 (2H, d, *J* = 5.4 Hz, H-7′) and *δ* 39.4 (C-7′), 5.92 (1H, m, H-8′) and 137.7 (C-8′), and 5.03 (2H, m, H-9′) and 114.4 (C-9′) showed the existence of an allyl group. The long-range correlations between H-2′ and C-4′, C-7′, H-6′ and C-4′, C-7′, H-5′ and C-1′, C-3′, and methoxyl protons (*δ* 3.76) and C-3′ were observed in the HMBC spectrum (Figure 3), which led to the trisubstituted aromatic ring being determined as a 1-allyl -3-methoxylphenyl unit.

The relative configuration of **2** was established from the NOESY spectrum and the coupling constants between H-7 and H-8. The NOE cross peak of H-7 and H-8 was observed in the NOESY spectrum, suggested the *cis*-configuration between H-7 and H-8. Moreover, the coupling constant *J*_7,8_ = 5.4 Hz demonstrated the relative configuration to be erythro [29,30]. The positive cotton effect appearing at 241 nm in the CD spectrum indicated the absolute configuration of **2** was 7*R*, 8*S* [31,32]. Thus the structure of **2** was determined to be erythro-(7*R*, 8*S*)-7-(3-methoxyl-4-hydroxylphenyl)-8-(1′-allyl-3′-methoxylphenoxyl) propane-7,9-diol, which named as neoasarinin B.

The spectroscopic data of compound **2** are as follows: amorphous powder; ${\left[\alpha \right]}_{D}^{20}$ –3.6 (*c* 0.55, MeOH); UV (MeOH) *λ*_max_ (log *ε*) 211 (4.03), 229 (3.95), 280 (3.55) nm; CD (MeOH) *λ*_max_ (*c* 0.58 × 10^−4^ mol/L, Δ*ε*) 241 (+0.54), 274 (+0.04), 300 (–0.15); IR (KBr) *ν*_max_ 3423, 2936, 1639, 1605, 1511, 1463, 1429, 1268, 1223, 1153, 1126, 1031, 916, 817 cm^−1^; ^1^H-NMR (CD_3_OD, 600 MHz) and ^13^C-NMR (CD_3_OD, 150 MHz) data, see Table 1; HRMALDIMS *m/z* 383.1457 [M + Na]^+^ (calcd. for C_20_H_24_O_6_Na, 383.1465).

Compound **3** was isolated as an amorphous powder. The molecular formula of it was determined as C_20_H_24_O_6_ on the basis of HRESIMS (*m/z* 383.1455 [M + Na]^+^, calcd. 383.1465). Compound **3** had the same molecular formula as **2**. And IR, UV, ^1^H- and ^13^C-NMR spectra of the two compounds were very similar to each other, which revealed the identical plane structure of them with a 3-methoxyl-4-hydroxyphenyl unit and a 1-allyl-3-methoxylphenyl unit. The major differences between **2** and **3** were the chemical shift values of H-8, 9, and C-8, 9, and three aromatic protons for the 1-allyl-3-methoxylphenyl unit. The chemical shift values of *δ* 6.81 (H-2′), 6.98 (H-5′) and 6.69 (H-6′) for the 1-allyl-3-methoxylphenyl unit of **3** were all more downfield than those of **2** (*δ* 6.75 (H-2′), 6.83 (H-5′) and 6.64 (H-6′)). Furthermore, the more downfield chemical shift of C-8 (*δ* 86.3) of **3** was observed, and the chemical shifts of H-9 (*δ* 3.70 (1H, dd, *J* = 12.0, 3.6 Hz, H-9a) and *δ* 3.41 (1H, m, H-9b)) of **3** were more highfield than those of **2** (*δ* 3.75–3.83 (2H, m)). Through the analysis of the above data, it could be concluded that **3** and **2** were stereoisomers with different configurations at the chiral centers of C-7 and C-8, respectively.

The coupling constant between H-7 and H-8 was *J*_7,8_ = 6.0 Hz, suggesting the relative configuration of **3** to be threo [29,30], which confirmed no correlation between H-7 and H-8 in the NOESY spectrum. The CD spectrum of **3** showed the positive cotton effect at 238 nm, which established that the absolute configurations at C-7 and C-8 were 7*S* and 8*S*, respectively [31,32]. On the basis of the above data, the structure of **3** was concluded to be threo-(7*S*, 8*S*)-7-(3-methoxyl-4-hydroxyphenyl)-8-(1′-allyl-3′-methoxylphenoxyl) propane-7,9-diol, and it was named neoasarinin C.

The spectroscopic data of compound **3** are as follows: amorphous powder; ${\left[\alpha \right]}_{D}^{20}$ –5.3 (*c* 0.38, MeOH); UV (MeOH) *λ*_max_ (log *ε*) 205 (3.33), 230 (2.77), 279 (2.38) nm; CD (MeOH) *λ*_max_ (*c* 0.61 × 10^−4^ mol/L, Δ*ε*) 238 (+0.18), 288 (–0.18); IR (KBr) *ν*_max_ 3421, 2921, 1700, 1606, 1511, 1462, 1429, 1266, 1222, 1132, 1030, 874 cm^−1^; ^1^H-NMR (CD_3_OD, 600 MHz) and ^13^C-NMR (CD_3_OD, 150 MHz) data, see Table 1; HRESIMS *m/z* 383.1455 [M + Na]^+^ ( calcd. for C_20_H_24_O_6_Na, 383.1465).

Compound **4** was obtained as an amorphous powder. Its molecular formula was determined to be C_24_H_18_O_13_ by HRESIMS (*m/z* 525.1605 [M + H]^+^, calcd. 525.1602). The IR spectrum showed absorptions for hydroxyl (3133 cm^−1^) and conjugated carbonyl groups (1630 cm^−1^). The ^1^H- and ^13^C-NMR data were similar to those of the known threo-guaiacylglycerol-β-*O*-4′-vanillic acid ether, which revealed the plane structure of **4** to be a breakdown product of 8-*O*-4′ type of neolignan [33]. However, the major difference between **4** and threo-guaiacylglycerol-β-*O*-4′-vanillic acid ether was the presence of a methylenedioxy at *δ* 5.77 (2H, s) in **4**. The HMBC correlations between H-2 and C-4, H-5 and C-3, C-1, H-6 and C-4, and the methylenedioxy protons (*δ* 5.77) and C-3, C-4 suggested the existence of a 3,4-methylenedioxyphenyl group instead of the 3-methoxyl-4-hydroxyphenyl unit (Figure 3). In addition, the ^1^H- and ^13^C-NMR spectra of **4** exhibited the signal of a set of β-glucopyranose, including the anomeric proton at *δ* 5.58 (1H, d, *J* = 7.8 Hz, H-1″) and the six characteristic aliphatic carbon signals at *δ* 94.8, 77.5, 76.7, 72.7, 69.7, and 61.0. The glycosidic linkage was determined to be at the formyl group (*δ* 165.1) by the correlation observed between H-1″ and C-7′ in HMBC experiments (Figure 3). Acid hydrolysis of **4** afforded a D-glucose. The HMBC spectrum also showed the correlations between H-2, H-6 and C-7, H-8 and C-4′, and H-2′, H-6′ and C-7′, which further supported the above conclusions.

The coupling constant value of 4.8 Hz between H-7 and H-8 revealed the erythro configuration [29,30]. Furthermore, a negative Cotton effect at 240 nm indicated that the absolute configuration of **4** was 7*S*, 8*R* [31,32]. From these data, the structure of **4** was, therefore, established as erythro-(7*S*, 8*R*)-7-(3,4-methylenedioxyphenyl)-8-[1′-(formyl-β-d-glucopyranosyl)-3′-methoxylphenoxyl) propane-7,9-diol, and named as neoasarininoside A.

The spectroscopic data of compound **4** are as follows: amorphous powder; ${\left[\alpha \right]}_{D}^{20}$ +30.6 (*c* 0.52, MeOH); UV (MeOH) *λ*_max_ (log *ε*) 220 (4.01), 263 (3.81), 289 (3.71) nm; CD (MeOH) *λ*_max_ (*c* 0.36 × 10^−4^ mol/L, Δ*ε*) 240 (–0.13), 264 (+0.30), 298 (+0.14); IR (KBr) *ν*_max_ 3133, 1630, 1605, 1400, 1349, 760 cm^−1^; ^1^H-NMR (CD_3_OD, 600 MHz) and ^13^C-NMR (CD_3_OD, 150 MHz) data, see Table 2. ESIMS *m/z* 542 [M + NH_4_]^+^, 547 [M + Na]^+^; HRESIMS *m/z* 525.1605 [M + H]^+^ (calcd. for C_24_H_29_O_13_, 525.1602).

Compound **5** was isolated as a needle crystal. The molecular formula of it was determined as C_26_H_32_O_13_ on the basis of HRESIMS (*m/z* 551.1776 [M − H]^−^, calcd. 551.1770). The IR spectrum showed absorptions for hydroxyl (3422 cm^−1^) and conjugated carbonyl groups (1688 cm^−1^). The ^1^H and ^13^C-NMR spectrum of **5**, similarity with the known compound guaiacylglycerol-β-*O*-4′-ferulic acid ether [34], were attributed to two 1,3,4-trisubstituted aromatic rings (*δ* 7.09 (1H, d, *J* = 1.2 Hz, H-2) and 111.0 (C-2), 7.07 (1H, overlapping, H-5) and 116.21 (C-5), and 6.94 (1H, dd, *J* = 7.8, 1.2 Hz, H-6) and 119.8 (C-6), 7.12 (1H, overlapping, H-6′) and 110.8 (C-6′), 6.93 (1H, d, *J* = 7.8 Hz, H-3′) and 116.15 (C-3′), and 7.04 (1H, dd, *J* = 7.8, 1.8 Hz, H-4′) and 122.0 (C-4′)), a (*E*)-formylvinyl unit (*δ* 7.54 (1H, d, *J* = 15.6 Hz, H-7′) and 144.8 (C-7′), 6.31 (1H, d, *J* = 15.6 Hz, H-8′) and 116.11 (C-8′), and carboxyl carbon at *δ* 169.5 (C-9′)), two oxygenated methine protons (*δ* 4.84 (1H, d, *J* = 4.8 Hz, H-7) and 72.4 (C-7), and 4.47 (1H, m, H-8) and 84.1 (C-8)), an oxygenated methylene group (*δ* 3.85–3.76 (1H, overlapping, H-9a,), 3.65 (1H, dd, *J* = 12.0, 4.8 Hz, H-9b) and 60.8 (C-9)), and two methoxyl groups (*δ* 3.79 (6H, s) and 55.3), indicating **5** was an 8-*O*-4′ type of neolignan derivative. Comparing the NMR data of **5** with those of guaiacylglycerol-β-*O*-4′-ferulic acid ether, there was not a ferulic acid unit in the structure of **5** but an isoferulic acid unit, which further supported by the correlations between H-6′ and C-7′, C-4′, H-3′ and C-1′, C-5′, H-4′ and C-2′, H-7′ and C-6′, C-4′ and C-9′, H-8′ and C-5′, the methoxyl protons (*δ* 3.79) and C-2′ in HMBC spectrum (Figure 3). Moreover, the HMBC correlations between H-8 and C-1′, H-7 and C-2, C-6, H-2 and C-7 further confirmed the above conclusions (Figure 3). The presence of a β-glucopyranosyl moiety in the molecule was suggested by the NMR spectrum, which showed six carbon signals at *δ* 101.6, 76.8, 76.5, 73.6, 69.9, and 61.2, and an anomeric proton signal at *δ* 4.80 (1H, d, *J* = 7.2 Hz, H-1″). Acid hydrolysis and GC analysis proved the absolute configuration of the sugar moiety to be D-glucose. The HMBC correlation between H-1″ and carboxyl carbon (*δ* 169.5) was observed, confirming the β-d-glucopyranosyl group linked to C-9′. The assignments of the ^1^H and ^13^C-NMR data were established by the analysis of the ^1^H-^1^H COSY, HMQC and HMBC spectra. The coupling constant *J*_7,8_ was 4.8 Hz, which was indicative of the erythro configuration between C-7 and C-8 [29,30]. The absolute configurations, 7*S*, 8*R*, were assigned on the basis of a negative Cotton effect at 224 nm [31,32]. As a result, **5** was determined to be erythro-(7*S*, 8*R*)-7-(3-methoxyl-4-hydroxyphenyl)-8-{5′-[(*E*)-formylvinyl-β-d-glucopyranosyl]-2′-methoxylphenoxyl} propane-7,9-diol, and named neoasarininoside B.

The spectroscopic data of compound **5** are as follows: needle crystal (MeOH); m.p. 178–179 °C; ${\left[\alpha \right]}_{D}^{20}$ –23.3 (*c* 0.60, MeOH); UV (MeOH) *λ*_max_ (log *ε*) 205 (4.10), 284 (3.89), 319 (3.91) nm; CD (MeOH) *λ*_max_ (*c* 0.39 × 10^−4^ mol/L, Δ*ε*) 224 (–1.17), 279 (–0.45); IR (KBr) *ν*_max_ 3422, 2935, 1688, 1632, 1598, 1510, 1464, 1421, 1265, 1136, 1074, 1030, 755 cm^−1^; ^1^H-NMR (CD_3_OD, 600 MHz) and ^13^C-NMR (CD_3_OD, 150 MHz) data, see Table 2; ESIMS *m/z* 570 [M + NH_4_]^+^, 575 [M + Na]^+^; HRESIMS *m/z* 551.1776 [M − H]^−^ (calcd. for C_26_H_31_O_13_, 551.1770).

**Figure 4:**
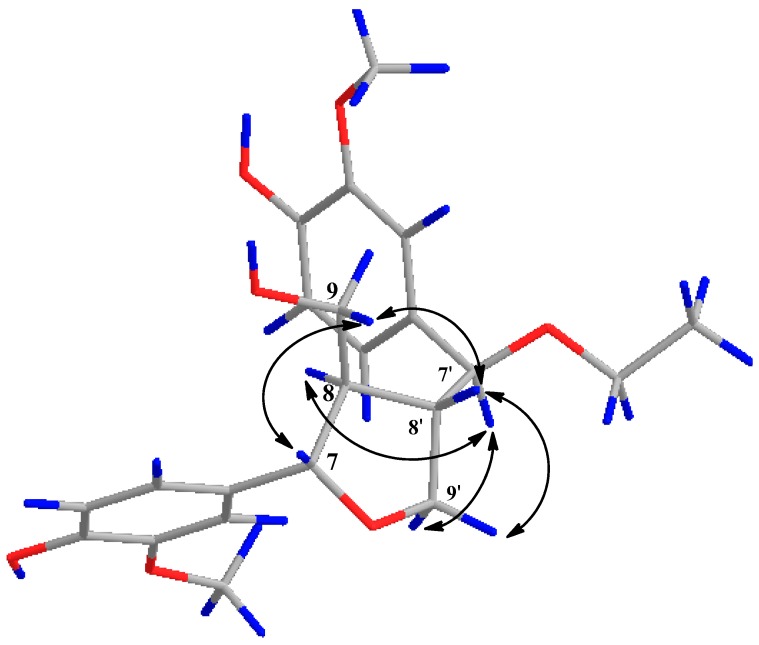
Key NOESY correlations (H ↔ H) of compound **6**.

Compound **6** was obtained as an amorphous powder, with the molecular formula of C_22_H_28_O_7_ derived from the ion peak at *m/z* 427.17261 [M + Na]^+^ (calcd 427.1727) in HRESIMS. The IR spectrum indicated the presence of a hydroxyl group (3412 cm^−1^). All the ^1^H-NMR, ^13^C-NMR, ^1^H-^1^H COSY, and HMBC data are in good agreement with those of 4-[1-ethoxy-1-(4-hydroxy-3-methoxy) phenyl]methyl-2-(4-hydroxy-3-methoxy)phenyl-3-hydroxymethyl-tetrahydrofuran [35]. The NOESY correlation between H-7 and H-9 indicated that the two protons, H-7 and H-8, were oriented as trans with each other. The NOE cross peaks were observed at H-7′ and H-9′a, H-8′ and H-9′b, which indicated that the relative configuration between C-7′ and C-8′ was trans. On the other hand, the trans configuration between C-8 and C-8′ was determined by the NOESY correlations between H-8′ and H-9, H-7′ and H-8 (Figure 4). The absolute configuration of **6** was determined by comparing the CD spectrum of **6** with those of tanegool and analogues [36,37]. The CD spectrum of **6** showed positive absorption peaks at 235 and 278 nm, which were identical with the known compound tanegool. Thus, the structure of **6** was determined to be (7*S*, 8*R*, 7′*R*, 8′*S*)-7′-[7′-ethoxyl-7′-(4′-hydroxyl-3′-methoxylphenyl)]methyl-7-(4-hydroxyl-3-methoxylphenyl)-8-hydroxymethyl-tetrahydrofuran and named asarinin A.

Compound **7** was obtained as a white amorphous powder. The molecular formula of it was determined as C_21_H_24_O_7_ on the basis of HRESIMS (*m/z* 411.1409 [M + Na]^+^, calcd. 411.1414). The IR absorption of **7** was indicative of the presence of hydroxyl (3446 cm^−1^). The UV spectrum showed maximum absorption at 241 and 280 nm, which corresponded to the furofuran type of lignan [38]. The ^1^H- and ^13^C-NMR spectra of **7** also indicated that it was an unsymmetrically substituted furofuran lignan. The ^1^H-NMR signals of an ABX system at *δ* 6.91 (1H, d, *J* = 1.2 Hz, H-2′), 6.89 (1H, d, *J* = 7.8 Hz, H-5′) and 6.83 (1H, dd, *J* = 7.8, 1.2 Hz, H-6′), the other two aromatic protons at 6.60 (2H, s, H-2, 6), together with three methoxyl groups at *δ* 3.91 (3H, s) and *δ* 3.90 (6H, s) showed the existence of a 4-hydroxyl-3-methoxylphenyl unit and a 4-hydroxyl-3,5-dimethoxylphenly unit. Comparing NMR data of **7** with those of (+)-de-4, 4′-*O*-dimethylepimagnolin [39], which was a microbial transformation product of (+)-epimagnolin by a specific type of *Aspergillus*, compound **7** presented identical spectroscopic data with the known compound. Although **7** exhibited the same ^1^H-NMR data as (+)-de-4, 4′-*O*-dimethylepimagnolin A, which has the absolute configuration 7*S*, 8*R*, 7′*R*, 8′*R* with optical rotation value of ${\left[\alpha \right]}_{D}^{19}$ +107.7 (*c* 0.1, CHCl_3_), its optical rotation, ${\left[\alpha \right]}_{D}^{20}$ –126.7 (*c* 0.60, CHCl_3_), was opposite. Accordingly, the absolute configuration was assigned as 7*R*, 8*S*, 7′*S*, 8′*S* in **7** [40]. Finally, compound **7** was determined as (7*R*, 8*S*, 7′*S*, 8′*S*)-7-(4-hydroxyl-3, 5-dimethoxylphenyl)-7′-(4′-hydroxyl-3′-methoxylphenyl)-10, 10′- dioxabicyclo[3. 3. 0]-octane, and was named asarinin B.

The spectroscopic data of compound **7** are as follows: amorphous powder; ${\left[\alpha \right]}_{D}^{20}$ —126.7 (*c* 0.60, CHCl_3_); UV (CHCl_3_) *λ*_max_ (log *ε*) 241 (3.86), 280 (3.58) nm; IR (KBr) *ν*_max_ 3446, 1612, 1517, 1457, 1426, 1359, 1277, 1242, 1204, 1161, 1111, 1069, 1031, 998, 731 cm^−1^; ^1^H-NMR (CDCl_3_, 600 MHz) and ^13^C-NMR (CDCl_3_, 150 MHz) data, see Table 3. HRESIMS *m/z* 411.1409 [M + Na]^+^ (calcd. for C_21_H_24_O_7_Na, 411.1414).

**Figure 5:**
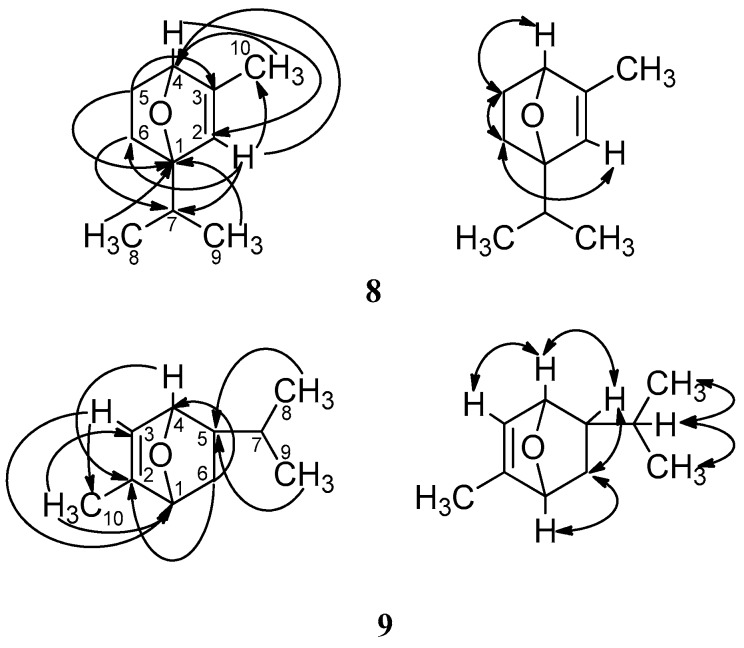
Key HMBC (H → C) and ^1^H-^1^H COSY correlations (H ↔ H) of compounds **8** and **9**.

Compound **8** was isolated as a white powder. Its molecular formula was found to be C_10_H_16_O by HRESIMS (*m/z* 153.1273 [M + H]^+^, calcd. 153.1273). The IR absorption spectrum of **8** showed the presence of a double bond (1608 cm^−1^). The ^1^H-NMR and HMQC spectra contained the signals for an olefinic proton at *δ* 5.54 (1H, t, *J* = 4.2, 1.2 Hz, H-2), an oxygenated methine at *δ* 3.94 (1H, t, *J* = 3.0, 2.4 Hz, H-4), two methylenes (*δ* 2.09 (1H, m, H-6a), 1.77 (1H, m, overlapping, H-6b), 1.94 (1H, dt, *J* = 13.8, 2.4 Hz, H-5a), 1.35 (1H, ddd, *J* = 13.2, 3.5 Hz, H-5b)), a methylene at *δ* 1.77 (1H, m, overlapping, H-7) and three methyl groups (*δ* 1.75 (3H, s, CH_3_-10), 1.15 (6H, s, CH_3_-8, 9)). The ^13^C-NMR data of **8** showed the existence of two olefinic carbons at *δ* 134.0 (C-3) and 124.8 (C-2), an oxygenated quaternary carbon at *δ* 71.4 (C-1), an oxygenated methine carbon at *δ* 67.9 (C-4), a methine carbon at *δ* 38.4 (C-7), two methylene carbons at *δ* 32.8 (C-5) and 26.7 (C-6), and three methyl carbons (*δ* 25.7 (C-8), 25.6 (C-9), and 19.8 (C-10)). Combined analysis of the spectroscopic data of **8** suggested that it was very similar to 2-methyl-5-isopropyl-7-oxabicyclo[2.2.1]hept-2-ene [41]. The principal differences between these two compounds were the substitution sites of the isopropyl group and the methyl group, linked to the 7-oxabicyclo[2.2.1]hept-2-ene moiety, respectively. In the HMBC spectrum, the correlations between H-2 and C-10, the methyl protons at *δ* 1.75 and C-4, indicated that the methyl at *δ* 1.75 was attached to C-3. Moreover, the isopropyl group was determined to connect with C-1, which was supported by HMBC correlations from H-2 to C-7, from H-6 to C-7, and the two methyl protons at *δ* 1.15 to C-1 (Figure 5). All other HMBC correlations observed were consistent with the structure of **8**. The relative configuration of **8** was determined by analysis of its NOESY spectrum, which showed no NOE correlation between H-4 and CH_3_-8, 9, suggesting that H-4 was on the opposite face of the molecule relative to the isopropyl group. Finally, the assignments of all protons and carbons were made by HMQC, HMBC, ^1^H-^1^H COSY spectra (Figure 5). Thus, the structure of **8** was elucidated as 1-isopropyl-3-methyl-7-oxabicyclo[2.2.1]hept-2-ene and named asaricin A.

The spectroscopic data of compound **8** are as follows: white powder; ${\left[\alpha \right]}_{D}^{20}$ –13.3 (*c* 0.60, MeOH); UV (MeOH) *λ*_max_ (log *ε*) 202 (3.07) nm; IR (KBr) *ν*_max_ 3340, 2975, 2931, 2891, 1608, 1471, 1435, 1378, 1313, 1293, 1253, 1159, 1054, 1033, 918, 666, 617 cm^−1^; ^1^H-NMR (CD_3_OD, 600 MHz) and ^13^C-NMR (CD_3_OD, 150 MHz) data, see Table 4. EIMS *m/z* 152 (15), 137 (29), 109 (100), 95 (27), 79 (66), 59 (90), 55 (22), 43 (71); HRESIMS *m/z* 153.1273 [M + H]^+^ (calcd. for C_10_H_17_O, 153.1273).

Compound **9** was obtained as a colorless prismatic crystal. The molecular formula of it was determined as C_10_H_16_O by HRESIMS (*m/z* 153.1271 [M + H]^+^, calcd. 153.1273). The IR spectrum displayed the absorption of a double bond (1632 cm^−1^). Comparing the ^1^H- and ^13^C-NMR data of **9** with those of asiasarinol [42], the existence of a 7-oxabicyclo[2.2.1]hept-2-ene moiety, a isopropyl group, and a methyl group in **9** was inferred. Furthermore, the locations of the isopropyl group and the methyl, connected with **9**, were determined by an HMBC experiment. The HMBC correlations of the methyl protons at *δ* 1.76 with C-3 and C-1, H-3 and C-10 supported the idea that the methyl at *δ* 1.76 was linked to C-2. In addition, the HMBC correlations from H-8, H-9 to C-5 revealed that the isopropyl was attached to C-5. The ^1^H-^1^H COSY spectrum of **9** further reinforced the above conclusions by the correlation of H-3 with H-4, H-4 with H-5, H-5 with H-6, H-6 with H-1 (Figure 5). On the basis of the above evidence, the structure of **9** was identified as 2-methyl-5-isopropyl-7-oxabicyclo[2.2.1]hept-2-ene, named asaricin B. Although reported as an intermediate product during the synthesis process of *α*-Phellandrene previously [41], asaricin B was first isolated from a plant.

The spectroscopic data of compound **9** are as follows: colorless prismatic crystal (EtOAc); mp 98–99 °C; ${\left[\alpha \right]}_{D}^{20}$ +13.6 (*c* 0.66, MeOH); UV (MeOH) *λ*_max_ (log *ε*) 205 (4.64) nm; IR (KBr) *ν*_max_ 3259, 2963, 2948, 2883, 1632, 1465, 1446, 1384, 1365, 1255, 1212, 1165, 1128, 1072, 1027, 989, 868, 707 cm^−1^; ^1^H-NMR (CD_3_OD, 600 MHz) and ^13^C-NMR (CD_3_OD, 150 MHz) data, see Table 4. EIMS *m/z* 152 (2), 137 (9), 127 (16), 111 (74), 109 (40), 100 (83), 71 (100), 55 (29), 43 (67); HRESIMS *m/z* 153.1271 [M + H]^+^ (calcd. for C_10_H_17_O, 153.1273).

**Figure 6:**
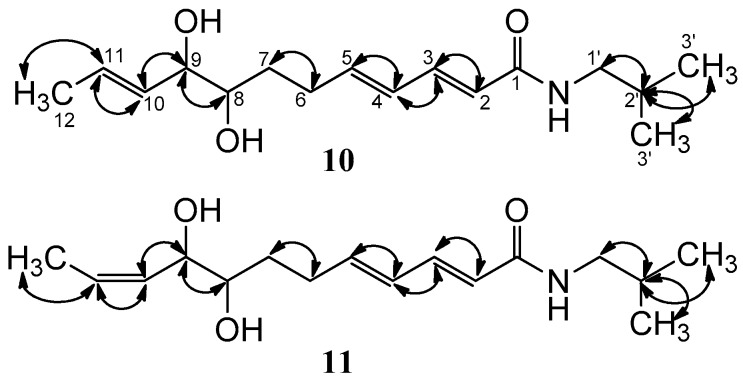
Key 1H-1H COSY correlations (H ↔ H) of compounds **10** and **11**.

Compound **10** was obtained as a white amorphous powder. Its molecular formula was established as C_16_H_27_NO_3_ by HRESIMS (*m/z* 282.2069 [M + H]^+^, calcd. 282.2063). ESI-MS gave the ion peak at *m/z* 282 [M + H]^+^, 264 [M + H − H_2_O]^+^, 246[M + H − 2H_2_O]^+^, indicating the presence of two hydroxyl groups in the structure of **10**. The IR bands at 3417 cm^−1^ and 1676 cm^−1^ were attributed to hydroxyl and carbonyl groups, respectively. The UV maximal absorption near 256 nm and the ^1^H-NMR signals of **10** at *δ* 0.91 (6H, m, H-3′, 4′), 1.78 (1H, m, H-2′) and 3.05 (2H, d, *J* = 7.0 Hz, H-1′) suggested the presence of an *N*-isobutylamide group [43]. Comparing the ^1^H- and ^13^C-NMR data of **10** with those of asaramid [42], it showed the presence of the (2*E*,4*E*)-2,4-dienamide moiety and an allylic methylene (*δ* 2.35 (2H, m, H-6) and 28.9 (C-6)). In addition, the ^1^H-, ^13^C-NMR and DEPT spectra of **10** indicated the existence of one methyl, two oxygenated methines, two olefinic methines, and a methylene. Furthermore, the geometry of the additional double bond between C-10 and C-11 was determined to be the *E*-form, which was supported by the coupling constant of *J*_10,11_ = 15.0 Hz. The ^1^H-NMR signals of two downfield shifted oxygenated methine protons at *δ* 3.38 (1H, m, H-8) and 3.81 (1H, m, H-9) implied that C-8 and C-9 bear one hydroxyl group. Moreover, the locations of double bonds between C-10 and C-11, and two hydroxyl groups at C-8 and C-9, respectively, were further confirmed by the ^1^H-^1^H COSY experiment, in which the methyl protons at *δ* 1.70 (3H, d, *J* = 6.0 Hz) coupled with the olefinic proton at *δ* 5.71 (1H, m, H-11), and the other olefinic proton at *δ* 5.48 (1H, dd, *J* = 15.0, 7.2 Hz, H-10) coupled with H-9, and H-9 coupled with H-8 (Figure 6). According to the IR, UV, ^1^H- and ^13^C-NMR, DEPT, ^1^H-^1^H COSY, and MS data, the structure of **10** was elucidated as (2*E*, 4*E*, 10*E*)-8, 9-dihydroxy-*N*-isobutyl-2, 4, 10-dodecatrienamide, and named asaramid II.

The spectroscopic data of compound **10** are as follows: white amorphous powder; ${\left[\alpha \right]}_{D}^{20}$ –1.7 (*c* 0.60, MeOH); UV (MeOH) *λ*_max_ (log *ε*) 256 (3.66) nm; IR (KBr) *ν*_max_ 3417, 2962, 1676, 1550, 1437, 1388, 1271, 1203, 1135, 1027, 719 cm^−1^; ^1^H-NMR (CD_3_OD, 600 MHz) and ^13^C-NMR (CD_3_OD, 150 MHz), see Table 5; ESIMS *m/z* 282 [M + H]^+^; HRESIMS *m/z* 282.2069 [M + H]^+^ (calcd. for C_16_H_28_NO_3_, 282.2063).

Compound **11** was obtained as a white amorphous powder, and had the same molecular formula as **10**, which was determined to be C_16_H_27_NO_3_ by HRESIMS (*m/z* 282.2063 [M + H]^+^, calcd. 282.2063). The IR, UV, ^1^H, and ^13^C-NMR data of **11** were very close to those of **10** except for the different chemical shift values of C-9 and C-12. The chemical shift values of **11** at *δ* 69.9 (C-9) and 12.3 (C-12) were at higher fields than *δ* 75.8 (C-9) and 16.7 (C-12) of **10**, respectively, which suggested that the geometry of the double bond between C-10 and C-11 was *Z*-form [44]. Moreover, the *Z*-configuration of the double bond was further confirmed by the coupling constant *J*_10,11_ = 9.0 Hz instead of 15.0 Hz. On the basis of IR, UV, ^1^H and ^13^C-NMR, DEPT, ^1^H-^1^H COSY (Figure 6), and MS data, the structure of **11** was identified as (2*E*, 4*E*, 10*Z*)-8, 9-dihydroxy-*N*-isobutyl-2, 4, 10-dodecatrienamide, and named aramid III.

The spectroscopic data of compound 11 are as follows: white amorphous powder; ${\left[\alpha \right]}_{D}^{20}$ –3.3 (*c* 0.60, MeOH); UV (MeOH) *λ*_max_ (log *ε*) 257 (3.64) nm; IR (KBr) *ν*_max_ 3408, 2962, 1678, 1551, 1437, 1386, 1270, 1204, 1136, 1026, 720 cm^−1^; ^1^H-NMR (CD_3_OD, 600 MHz) and ^13^C-NMR (CD_3_OD, 150 MHz), see Table 5; ESIMS *m/z* 282 [M + H]^+^; HRESIMS *m/z* 282.2063 [M + H]^+^ (calcd. for C_16_H_28_NO_3_, 282.2063).

The 37 known compounds (**6**, **12**–**47**), which comprise six structural classes, were fully identified by comparison of their physical and spectroscopic data with those reported in the corresponding literature. They were 14 lignans: asarinin A (**6**) [35], (1*R*, 2*S*, 5*R*, 6*R*)-5′-*O*-methylpluviatilol (**13**) [26], (–)-asarinin (**14**) [3], (–)-sesamin (**15**) [3], xanthoxylol (**16**) [45], clemaphenol A (**17**) [46], epipinoresinol (**18**) [47], neo-olivil (**19**) [48], (–)-piperitol (**20**) [49], episesaminone (**21**) [50], (7′*R*)-7′-hydroxylariciresinol (**22**) [51], tanegool (**23**) [35], (–)-tanegol (**24**) [52], morinols G (**25**) [53]; six flavanones: (2*S*)-naringenin (**26**) [54], (2*S*)-naringenin-5-*O*-β-d-glucopyranoside (**27**) [55], (2*S*)-liquiritigenin-7-*O*-β-d-glucopyranoside (**28**) [56], (2*S*)-naringenin-7-*O*-β-d-glucopyranoside (**29**) [57], (2*S*)-naringenin-5, 7-di-*O*-β-d-glucopyranoside (**30**) [58], naringenin-5, 4′-di-*O*-β-d-glucopyranoside (**31**) [59]; four monoterpenes: 2-exo-*O*-β-d-glucosyl-5-hydroxy-borneol (**32**) [42], aisasarinol (**33**) [42], asiarinol A (**34**) [8], car-3-ene-2, 5-dione (**35**) [8]; four straight chain amide derivatives: *N*-*trans*-coumaroyltyramine (**36**) [60], asaramid (**37**) [42], pellitorine (**38**) [7], *N*-isobutyl-3,4-methylenedioxy cinnamide (**39**) [61]; seven benzene derivatives: 3-methoxyl-5-methylbenzene-1-*O*-β-d-glucopyranoside (**12**) [62],1-(3′,4′-dihydroxy) phenyl-6,7-dihydroxy-isochroman (**40**) [63], *trans*-ferulic acid (**41**) [64], 2-(3, 4-dihydroxy)-phenyl-ethyl-*O*-β-d-glucopyranoside (**42**) [65], vanillic acid 1-*O*-β-d-glucopyranoside (**43**) [66], 3-(3,4-methylenedioxyphenyl)-1,2-propanediol (**44**) [67], 2, 3-dihydro-2-methyl-6-hydroxybenzofuran-5-carboxylic acid (**45**) [68]; and two fatty glycerides: glyceryl-1-stearate (**46**) [69], 2, 3-dihydroxypropyl-16-hydroxyhexadecanoate (**47**) [70]. The 15 known compounds **17**, **19**, **22**–**25**, **28**, **31**, **36**, **40**, **42**, **43**, and **45**–**47** were isolated for the first time from the genus *Asarum*, and five known compounds **16**, **32**, **33**, **37**, and **39** were first reported from *A. heterotropoides* var. *mandshuricum*.

Although previous studies had reported the isolation of bisepoxylignans and monoepoxylignans from the genus *Asarum* [3,5], five 8-*O*-4′ type neolignans, **1**–**5**, were reported in this genus for the first time. In addition, 1-*O*-feruloyl-β-d-glucopyranoside was found in *Asiasarum sieboldi* [58], but it was interesting that **5** contained the 1-*O*-isoferuloyl-β-d-glucopyranosyl unit in its structure, which was not reported in the compounds isolated from the genus *Asarum*.

**Figure 7:**
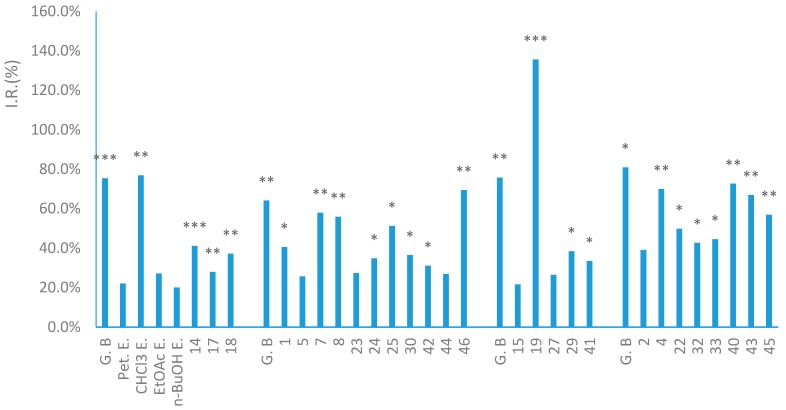
Isolated compounds and extracts from roots and rhizomes of *Asarum heterotropoides* var. *mandshuricum* inhibit the release of β-glucuronidase in PMN cells induced by the PAF. Asterisks indicate significant differences comparing with control, * *p* < 0.05, ** *p* < 0.01, *** *p* < 0.001.

As mentioned in the introduction, a few papers have reported some anti-inflammatory compounds isolated from *A. heterotropoides* var. *mandshuricum* and *A. sieboldi*, which showed the potent inhibition of (–)-sesamin (**15**) in both LPS-induced NO production in BV-2 microglial cells and TNFα-induced NF-κB transcriptional activity in HepG2 cell assays [25,26], along with (1*R*, 2*S*, 5*R*, 6*R*)-5′-*O*-methylpluviatilol (**13**), (–)-asarinin (**14**) and epipinoresinol (**18**) in LPS-activated macrophages RAW264.7 assay [5], but there has been no prior report on the evaluation of the anti-inflammatory effects of non-volatile compounds obtained from *A. heterotropoides* var. *mandshuricum* in PAF-induced β-glucuronidase release in a PMNs cell assay. Thus, 37 of the isolates, including 18 lignans (**1**, **2**, **4**–**7**, **13**–**19** and **21**–**25**), five flavanones (**27**–**31**), four monoterpenes (**8**, **9**, **32** and **33**), one amide derivative (**37**), seven benzene derivatives (**12** and **40**–**45**), and two fatty glycerides (**46** and **47**), at 10^−5^ mol/L, were screened for anti-inflammatory activity in vitro by assessing the inhibitory rate of the release of β-glucuronidase in polymorphonuclear leukocytes (PMNs) induced by the platelet-activating factor (PAF). Ginkgolide B was used as the positive control and gave inhibitory rates of 64.1%–80.9% for four different 96-well plates (1^#^–4^#^) at 10^−5^ mol/L. The Student’s *t*-test for unpaired observations between the model (stimulated by PAF alone) and the control (cultured in medium alone) or tested samples was carried out to identify statistical differences; * p < 0.05, ** *p* < 0.01, *** *p* < 0.001; *p* < 0.05 were considered as significantly different. The results showed that 10 of the lignans (**1**, **4**, **7**, **14**, **17**–**19**, **22**, **24** and **25**), two of the flavonones (**29** and **30**), three of the monoterpenes (**8**, **32** and **33**), five of the benzene derivatives (**40**–**43** and **45**), and one fatty glyceride (**46**) possessed varying degrees of anti-inflammatory activity, as shown in Figure 7 and Table S1 in Supplementary Materials. However, the I.R. values of compounds **6**, **9**, **12**, **13**, **16**, **21**, **28**, **31**, **37**, and **47** were all less than 20%. Compound **19**, a monoepoxylignan, exhibited the highest inhibitory activity with a rate of 135.6% (*p* < 0.001), much more than that of the positive control of the 3^#^ 96-well plate (75.7%). Two other monoepoxylignans, compounds **22** and **24**, displayed anti-inflammatory activity, with inhibitory rates of 49.7% (*p* < 0.05) and 34.7% (*p* < 0.05), respectively. Four bisepoxylignans, **7**, **14**, **17**, and 1**8**, also gave significant inhibition with a rate of 27.9%–57.9%. These lignans showing anti-inflammatory activities were observed to commonly bear a tetrahydrofuran ring in their structure. In addition, two 8-*O*-4′ neolignans, compounds **1** and **4**, also produced a strong inhibitory effect with a rate of 40.5% (*p* < 0.05) and 69.9% (*p* < 0.01), respectively. Moreover, one monoterpene, **8**, three benzene derivatives, **40**, **43**, and **45**, and one fatty glyceride compound, **46**, exhibited high inhibitory activity with rates of 55.8%, 72.6%, 66.9%, 56.9%, and 69.4% (*p* < 0.01, for each), respectively. For the other active compounds, monoterpenes **32** and **33**, flavonone glucosides **29** and **30**, and benzene derivatives **41** and **42**, the inhibitory rates of release of β-glucuronidase were 31.0%–44.4% (*p* < 0.05, for each). The other compounds showed weak or no inhibition activity in this study.

In this anti-inflammatory activity assay in vitro, we also tested the inhibitory activity of β-glucuronidase release of the different polarity extracts, including Pet. extract, CHCl_3_ extract, EtOAc extract, and *n*-BuOH extract obtained from the ethanol extract of roots and rhizomes of *A. heterotropoides* var. *mandshuricum*. The results showed that the CHCl_3_ extract displayed strong inhibitory activity with a rate of 76.8% (*p* < 0.01) at 10 μg/mL, while the other three extracts exhibited no anti-inflammatory activity (Figure 7). Moreover, by detailed analysis of the 21 anti-inflammatory active compounds, it was found that 13 of them were isolated from the CHCl_3_ extract, comprising nine lignans (**1**, **7**, **14**, **17**–**19**, **22**, **24**, and **25**), two benzene derivatives (**41** and **45**), one monoterpene (**8**), and one fatty glyceride (**46**). According to the results of the anti-inflammatory study, it was inferred that the lowly polar components that existed in the CHCl_3_ extract, especially lignans, as well as benzene derivatives, were the main active anti-inflammatory components of this traditional Chinese medicine. In addition, the anti-inflammatory active compound **14** was found to be the most abundant among all isolates—the amount obtained from 231 g CHCl_3_ extract was about 10 g, likely responsible for most of the anti-inflammatory effect of the CHCl_3_ extract. Based on these results, it is possible that one of the mechanisms for the anti-inflammatory effect of *A. heterotropoides* var. *mandshuricum* is due to the inhibition of the release of β-glucuronidase in PMN cells. However, a number of mediators and mechanisms are also involved in inflammatory reaction, so additional investigations are required to find the anti-inflammatory mechanisms and active components of *A. heterotropoides* var. *mandshuricum*.

## 3. Experimental Section

### 3.1. General

Melting points were obtained on an XT-4A micromelting point apparatus (Shanghai Hui Tong Optical Instrument Co., Ltd., Shanghai, China) without correction. Optical rotations were determined on a Perkin-Elmer 243B digital polarimeter (Boston, MA, USA). UV spectra were carried out on a Cary 300 UV-Vis spectrophotometer (Agilent Technologies, Santa Clara, CA, USA). CD spectra were measured on a JASCO J-810 spectropolarimeter (Jasco, Tokoyo, Japan). A Nicolet NEXUS-470 FTIR spectrophotometer (Madison, WI, USA) was used for scanning IR spectroscopy. NMR spectra were recorded on Varian INOVA-500 (Walnut Creek, CA, USA) and JOEL JNM-ECA600 spectrometers (JEOL, Tokyo, Japan). The chemical shifts are expressed as *δ* (ppm) values using solvent as an internal standard, and coupling constant, *J*, are in Hz. Mass spectra were detected with Bruker APEX IV FT (Bruker, Billerica, MA, USA) and ABI Q-STAR mass spectrometers (Navarre, FL, USA). GC was carried out on a Shimadzu GC-2010 series system fitted with a FID detector (Shimadzu Corporation, Kyoto, Japan) and performed with a DB-1701 column (30 m × 0.25 mm i.d., 0.25 μm film thickness, Agilent Technologies, Santa Clara, CA, USA). Semi-preparative HPLC was conducted on an Alltima C_18_ column (10 mm i.d. × 250 mm, 10 μm, Alltech, Nicholasville, KY, USA) equipped with an Alltech 426 HPLC pump and an Alltech UVIS 2000 detector (Alltech, Nicholasville, KY, USA). Column chromatography was performed with silica gel (200–300 mesh, Qingdao Marine Chemical Co., Ltd., Qingdao, China) or Sephadex LH-20 gel (Pharmacia Co., Ltd., Shanghai, China). TLC analysis was performed on silica gel (400 mesh, Qingdao Marine Chemical Co., Ltd., Qingdao, China) and precoated polyamide plates (0.2 mm, Zhejiang Siqing Biochem Co., Ltd., Taizhou, China) plates. d-glucose was obtained from Beijing Chemical Reagent Company. All other chemical solvents used for isolation were of analytical grade (Beijing Beihua Fine Chemicals Co., Ltd., Beijing, China). Fractions were monitored by TLC and spots were detected by UV illumination or visualized by heating silica gel plates sprayed with 10% H_2_SO_4_ in 95% EtOH.

### 3.2. Plant Material

The roots and rhizomes of *Asarum heterotropoides* Fr. Schmidt var. *mandshuricum* (Maxim.) Kitag were purchased from Benxi City, Liaoning province, China, in May 2005, and authenticated by Professor Shao-Qing Cai of Peking University Health Science Center. The voucher specimen (No. 050526) was deposited in the Herbarium of Pharmacognosy, School of Pharmaceutical Sciences, Peking University, Beijing, China.

### 3.3. Extraction and Isolation

The air-dried and powdered roots and rhizomes of *A. heterotropoides* var. *mandshuricum* (36 kg) were extracted three times (2 h, 1.5 h, 1.5 h for each) under reflux with 95% ethanol and then three times (2 h, 1.5 h, 1.5 h for each) with 50% ethanol successively. The combined extracts were concentrated under reduced pressure to give a dark brown residue (9.2 kg), then 7.8 kg of it was suspended in H_2_O (16.5 L) and partitioned sequentially with petroleum ether (Pet.) (60–90 °C) (4 × 5 L), CHCl_3_ (4 × 5 L), EtOAc (4 × 5 L), and *n*-BuOH (4 × 5 L), respectively. The CHCl_3_ layer (231 g) was fractionated on silica gel CC eluting with a gradient of petroleum ether–EtOAc (10:1 to 0:1) to obtain 16 fractions, C1–C16. Fraction C11 was subjected to silica gel CC eluting with a gradient of CHCl_3_–MeOH (1:30 to 1:35) to yield four subfractions C11-1–C11-4. C11-1 was then subjected to chromatography on silica gel CC eluting with petroleum ether–Me_2_CO to produce seven subfractions C11-1-1–C11-1-7. C11-1-5 was chromatographed over silica gel with petroleum ether–Me_2_CO (2.5:1) as eluent, and subfraction was then separated by Sephadex LH-20 (CHCl_3_–MeOH, 6:4) and semi-preparative HPLC eluted with a gradient of MeOH–H_2_O to yield **7** (18 mg). C11-3 was subjected to silica gel CC eluting with petroleum ether–Me_2_CO (4.5:1) to yield **9** (4 mg). Fraction C12 was chromatographed on silica gel with a gradient of CHCl_3_–MeOH to give four fractions C12-1–C12-4. C12-3 was further divided into five subfractions C12-3-1–C12-3-5 with repeated silica gel CC eluting with CHCl_3_–MeOH (80:1 to 15:1). Chromatography of C12-3-1 on silica gel with CHCl_3_–EtOAc (5:1, 4:1) and CH_2_Cl_2_–Me_2_CO (7:1) as eluent successively and purified by semi-preparative HPLC eluting with a gradient of MeOH-H_2_O to yield **1** (23 mg), **2** (11.9 mg), **3** (2.1 mg) and **6** (5.1 mg). C12-4 was separated on silica gel CC eluting with CHCl_3_–MeOH (70:1 to 15:1) and further purified by C_18_ reversed-phase silica gel CC eluting with 70% MeOH in H_2_O to afford **8** (43 mg). Fraction C13 was chromatographed with a gradient of CHCl_3_–MeOH (35:1, 20:1, 5:1) as eluent to give six subfractions C13-1–C13-6. C13-6 was then subjected to silica gel CC with petroleum ether–Me_2_CO (30:1), and subfraction was further separated by semi-preparative HPLC with a gradient of MeOH–H_2_O as the mobile phase to yield **10** (12 mg) and **11** (6.5 mg).

The EtOAc layer (50 g) was fractionated on silica gel CC eluting with a gradient of CHCl_3_–MeOH (10:1 to 0:1) to yield 10 fractions, E1–E10. Fraction E6 was chromatographed on silica gel CC eluting with CHCl_3_–MeOH–H_2_O (15:1:0.1, 10:1:0.1), and purified by Sephadex LH-20 with MeOH to afford **12** (8.1 mg). Fraction E8 was separated on silica gel CC eluting with CHCl_3_–MeOH–H_2_O (8:1:0.1, 5:1:0.1) and then purified by semi-preparative HPLC with MeOH–H_2_O as eluent to yield **4** (3.9 mg).

The *n*-BuOH layer (400 g) was fractionated on silica gel CC eluting with a gradient of CHCl_3_–MeOH (7:1 to 0:1) to yield 14 fractions, B1–B14. Fraction B6 was further separated on silica gel CC eluting with CHCl_3_–MeOH–H_2_O (5:1:0.1 to 2:1:0.1) to give nine subfractions B6-1–B6-9. B6-7 was further divided by silica gel CC using CHCl_3_–MeOH–H_2_O (5:1:0.1), and purified by Sephadex LH-20 eluting with MeOH to afford **5** (20 mg). The extraction and isolation of known compounds **13**–**47** is described in the Supplementary Materials.

### 3.4. Acid Hydrolysis of Compounds ***4*** and ***5***, and GC Analysis

Compounds **4** (1 mg) and **5** (2 mg) in 2 N HCl (3 mL) were refluxed at 80 °C for 1 h. After cooling, the reaction mixture was neutralized with NaHCO_3_ and successively extracted with CHCl_3_ (4 × 3 mL). The aqueous layer was then evaporated to dryness and the residue was dissolved in anhydrous pyridine (200 μL), and l-cysteine methyl ester hydrochloride (0.06 mol/L, 200 μL) was added. The mixture was stirred at 60 °C for 1 h. Then 150 μL trimethylsilylation reagent hexamethyldisilazane–trimethyl chlorosilane (HMDS–TMCS, 3:1) was added, and the mixture was stirred at 60 °C for an additional 30 min. After centrifugation, the supernatant was concentrated under a stream of N_2_. The residue was portioned between *n*-hexane and H_2_O (0.2 mL each), and the *n*-hexane layer (2 μL) was analyzed by GC for sugar identification (detector, FID; injection temperature, 260 °C; detector temperature, 280 °C; temperature gradient system for the oven, 160 °C for 1 min and then raised to 230 °C at a rate of 5 °C/min; carrier gas, N_2_; flow rate, 1 mL/min). d-Glucose was identified for compounds **4** and **5** by comparison with retention time of authentic d-glucose (*t*_R_ = 20.76 min) after treatment in the same manner.

### 3.5. Anti-Inflammatory Activity Assay

Compounds and extracts used to test were dissolved in DMSO at the concentration of 10^−5^ mol/L and 10 μg/mL. A suspension of rat polymorphonuclear leukocytes (PMNs) (2.5 × 10^6^ cells/mL, 250 μL) was incubated at 37 °C for 15 min with the presence of the test sample (2.5 μL) (n = 3) [71]. Then cytochalasin B (1 mmol/L, 2.5 μL, Sigma-Aldrich, Shanghai, China) was added and incubated for 5 min, followed by activating with platelet-activating factor (PAF) (1 μmol/L, 2.5 μL, Sigma-Aldrich, Shanghai, China). After 10 min, the reaction was terminated in an ice bath. The supernatant was obtained by centrifugation at 4000 rpm for 5 min (4 °C), and then the supernatants (25 μL) and phenolphthalein glucuronic acid (2.5 mmol/L, 25 μL, Sigma-Aldrich, Shanghai, China) were incubated with acetic acid buffer (0.1 mol/L, 100 μL, pH 4.6) at 37 °C, 5% CO_2_, for 18 h. Finally, NaOH (0.2 mol/L, 150 μL) was added to terminate the reaction. The results were quantified by reading the absorbance at 550 nm by BIO-RAD model 450 enzyme-labeling instrument. The inhibitory rate (I.R.) was obtained by the following formula:

I.R. (%) = (OD_model_ − OD_sample_)/(OD_model_ − OD_control_) × 100%.

OD_model_, OD_sample_, and OD_control_ refer to the average absorbance of the four wells of PAF, the three wells of the test sample, and the control group, respectively.

## 4. Conclusions

The roots and rhizomes of *A. heterotropoides* var. *mandshuricum* are used in traditional Chinese medicine for the treatment of pain and inflammatory diseases [1]. Previous research proved that some compositions of the volatile oil, monoterpenes, lignans, and amides isolated from the genus *Asarum* showed potential anti-inflammatory activity [5,15,16,24,25,26]. In this paper, 47 compounds, including nine new compounds, one new natural compound, and 37 known compounds, were isolated and identified from the ethanol extract of this plant. Meanwhile, 8-*O*-4′ neolignans were reported in this genus for the first time. In order to evaluate the potential anti-inflammatory effects of non-volatile constituents separated from the roots and rhizomes of *A. heterotropoides* var. *mandshuricum*, 37 compounds were tested to assess the inhibitory rate of the release of β-glucuronidase in PMNs cells induced by PAF. The results showed that 10 of the lignans (**1**, **4**, **7**, **14**, **17**–**19**, **22**, **24**, and **25**), two of the flavonones (**29** and **30**), three of the monoterpenes (**8**, **32** and **33**), five of the benzene derivatives (**40**–**43** and **45**) and one fatty glyceride (**46**) possessed varying degrees of anti-inflammatory activity. The lignans, especially the tetrahydrofuran ring in their structures (**7**, **14**, **17**–**19**, **22**, and **24**), displayed significant anti-inflammatory activity. From an integrated analysis of previous research [5,25,26], it could be inferred that lignans are the main non-volatile constituents with anti-inflammatory activity. Additionally, the flavonones are commonly found in the genus *Asarum* [9,55,57,72,73], but there is no study reporting whether the flavonones obtained from this genus possess anti-inflammatory activity since this type of compound had been proved to be potentially useful for inhibiting the inflammatory response [74,75]. This study demonstrated that two flavonones (**29** and **30)** showed anti-inflammatory activity against the release of β-glucuronidase in PMNs cells, which is the first report of the anti-inflammatory effects of flavonones from this genus. Interestingly, (–)-asarinin (**14**), the effects of which had been reported for LPS-activated NO production in macrophages RAW264.7 cells and PAF-induced β-glucuronidase release in PMN cells in the literature [5] and in this paper, respectively, was the most abundant in this plant (Xixin) among all isolates. This primary finding provides support for further study of this compound for the development of a novel anti-inflammatory agent. The results provide a scientific explanation for the use of this plant as an herbal medicine in the treatment of inflammatory diseases.

## Figures and Tables

**Table 1:** NMR spectroscopic data (CD_3_OD) for compounds **1**–**3**.

Position	1		2		3	
	*δ*_C_	*δ*_H_ (*J* in Hz)	*δ*_C_	*δ*_H_ (*J* in Hz)	*δ*_C_	*δ*_H_ (*J* in Hz)
1	133.1	—	132.8	—	132.2	—
2	110.1	6.96, d (2.4)	110.5	7.00, d (2.4)	110.4	7.01, d (1.8)
3	147.4	—	147.4	—	147.5	—
4	145.5	—	145.7	—	145.9	—
5	114.4	6.73, d (8.4)	114.3 ^a^	6.73, d (8.4)	114.5 ^b^	6.75, d (8.4)
6	119.2	6.77, dd (8.4, 2.4)	119.6	6.82, dd (8.4, 2.4)	119.4	6.85, dd (8.4, 1.8)
7	73.0	4.87, d (4.2)	72.8	4.82, d (5.4)	72.8	4.88, d (6.0)
8	85.1	4.18, m	85.3	4.28, m	86.3	4.20, m
9	60.2	3.83, m, H-9a	60.8	3.75–3.83, m	60.5	3.70, dd (12.0, 3.6), H-9a
		3.62, dd (12.6, 3.6), H-9b				3.41, m, H-9b
1′	152.9	—	134.6	—	134.9	—
2′	97.4	6.41, d (3.0)	112.8	6.75, d (1.8)	112.7	6.81, d (1.8)
3′	156.0	—	150.6	—	150.4	—
4′	138.2	—	146.1	—	146.5	—
5′	132.6	—	118.3	6.83, d (7.2)	118.2	6.98, d (7.8)
6′	106.6	6.29, d (3.0)	120.7	6.64, dd (7.2, 1.8)	120.8	6.69, dd (7.8, 1.8)
7′	—	—	39.4	3.28, d (5.4)	39.5	3.33, m
8′	—	—	137.7	5.92, m	137.7	5.93, m
9′	—	—	114.4 ^a^	5.03, m	114.5 ^b^	5.03, m
3-OCH_3_	54.9	3.71, s	55.4	3.79, s	55.0	3.82, s
1′-OCH_3_	54.5	3.80, s				
3′-OCH_3_	55.0	3.81, s	55.3	3.76, s	55.2	3.84, s
5′-CH_3_	15.7	2.12, s				

^a−b^ Assignments may be interchanged in the same column.

**Table 2:** NMR spectroscopic data (CD_3_OD) for compounds **4** and **5**.

Position	4		5	
	*δ*_C_	*δ*_H_ (*J* in Hz)	*δ*_C_	*δ*_H_ (*J* in Hz)
1	135.1	—	136.2	—
2	107.4 ^a^	6.87, d (1.8)	111.0	7.09, d (1.2)
3	147.5	—	149.1	—
4	147.1	—	146.1	—
5	107.1 ^a^	6.59, d (7.8)	116.21 ^d^	7.07, m
6	120.4	6.78, m	119.8	6.94, dd (7.8, 1.2)
7	72.5	4.71, d (4.8)	72.4	4.84, d (4.8)
8	83.8	4.44, m	84.1	4.47, m
9	61.0 ^b^	3.77–3.74 ^c^, m	60.8	3.85–3.76 ^e^, m, H-9a 3.65, dd (12.0, 4.8), H-9b
1′	122.1	—	150.4	—
2′	113.0	7.46, d (1.2)	150.2	—
3′	149.7	—	116.15 ^d^	6.93, d (7.8)
4′	152.9	—	122.0	7.04, dd (7.8, 1.8)
5′	114.7	6.92, d (8.0)	128.4	—
6′	123.8	7.52, dd (7.8, 1.2)	110.8	7.12, m
7′	165.1	—	144.8	7.54, d (15.6)
8′	—	—	116.11 ^d^	6.31, d (15.6)
9′	—	—	169.5	—
1″	94.8	5.58, d (7.8)	101.6	4.80, d (7.2)
2″	72.7	3.62–3.32, m	73.6	3.45–3.36 , m
3″	76.7		76.5	
4″	69.7		69.9	
5″	77.5		76.8	
6″	61.0 ^b^	3.77–3.74 ^c^, m	61.2	3.85–3.76 ^e^, m
-OCH_2_O-	100.9	5.77, s	—	—
3-OCH_3_	—	—	55.3	3.79, s
2′-OCH_3_	—	—	55.3	3.79, s
3′-OCH_3_	55.2	3.75, s	—	—

^a−e^ Assignments may be interchanged in the same column.

**Table 3:** NMR spectroscopic data for **7** (in CDCl_3_).

Position	7	
	*δ*_C_	*δ*_H_ (*J* in Hz)
1	133.1	—
2	102.4	6.60, s
3	147.1	—
4	133.7	—
5	147.1	—
6	102.4	6.60, s
7	87.8	4.44, d (6.6)
8	54.5	2.90, dd (7.2, 13.8)
9	71.1	4.13, d (9.6) 3.87–3.83, m, H-9b
1′	129.6	—
2′	108.6	6.91, d (1.2)
3′	146.8	—
4′	145.4	—
5′	114.3	6.89, d (7.8)
6′	119.2	6.83, dd (7.8, 1.2)
7′	82.3	4.85, d ( 6.0)
8′	50.2	3.34–3.31, m, H-8′
9′	69.7	3.87–3.83, m, H-9′a 3.34–3.31, m, H-9′b
7′-OCH_2_-	—	—
-CH_3_	—	—
3-OCH_3_	56.4	3.90, s
5-OCH_3_	56.4	3.90, s
3′-OCH_3_	56.0	3.91, s
4-OH	—	5.60 ^a^, brs
4′-OH	—	5.48 ^a^, brs

^a^ Assignments may be interchanged in the same column.

**Table 4:** NMR spectroscopic data (CD_3_OD) for compounds **8** and **9**.

Position	8		9	
	*δ*_C_	*δ*_H_ (*J* in Hz)	*δ*_C_	*δ*_H_ (*J* in Hz)
1	71.4	—	67.0	3.90, d (3.0)
2	124.8	5.54, t (4.2, 1.2)	136.0	—
3	134.0	—	129.6	5.46, d (1.2)
4	67.9	3.94, t (3.0, 2.4)	68.6	3.85, dd (3.0, 1.2)
5	32.8	1.94, dt (13.8, 2.4), H-5a 1.35, ddd (13.8, 3.5), H-5b	41.5	1.58, m
6	26.7	2.09, m, H-6a 1.77, m, H-6b	29.5	1.71, dt (13.2, 3.0), H-6a 1.38, ddd (13.2, 4.2), H-6b
7	38.4	1.77, m,	25.6	2.10, m
8	25.7	1.15, brs	20.0	0.96, d (6.6)
9	25.6	1.15, brs	15.7	0.81, d (7.2)
10	19.8	1.75, s	19.3	1.76, s

**Table 5:** NMR spectroscopic data (CD_3_OD) for compounds **10** and **11.**

Position	10		11	
	*δ*_C_, Mult.	*δ*_H_ (*J* in Hz)	*δ*_C_, Mult.	*δ*_H_ (*J* in Hz)
1	167.7, C	—	167.6, C	—
2	121.8, CH	5.94, d (15.0)	121.8, CH	5.92, d (15.0)
3	140.7, CH	7.10, dd (15.0, 10.8)	140.7, CH	7.10, dd (15.0, 10.8)
4	128.0, CH	6.21, m	127.1, CH	6.22, m
5	142.2, CH	6.10, m	142.2, CH	6.11, m
6	28.9, CH_2_	2.35, m	28.9, CH_2_	2.38, m
7	31.6, CH_2_	2.23, m	31.3, CH_2_	2.22, m
8	73.6, CH	3.38, m	73.6, CH	3.49, m
9	75.8, CH	3.81, m	69.9, CH	4.29, m
10	130.6, CH	5.48, dd (15.0, 7.2)	129.7, CH	5.46, dd (9.0, 7.2)
11	128.6, CH	5.71, m	128.7, CH	5.65, m
12	16.7, CH_3_	1.70, d (6.0)	12.3, CH_3_	1.67, d (7.2)
1′	46.5, CH_2_	3.05, d (7.2)	46.7, CH_2_	3.05, d (7.2)
2′	28.3, CH	1.78, m	28.4, CH	1.78, m
3′, 4′	19.1, CH_3_	0.91, m	19.1, CH_3_	0.91, d (6.6)

## Supplementary Materials

Supplementary materials can be accessed at: http://www.mdpi.com/1420-3049/22/1/125/s1. The NMR, HR-MS and HPLC-MS/MS spectra of Compounds **1**–**11**, and the extraction and isolation of known compounds **13**–**47**, and anti-inflammatory activity assay in vitro are included in the Supplementary Data.
