# Supplementary material for: Chemical Constituents from the Roots and Rhizomes of Asarum heterotropoides var. mandshuricum and the In Vitro Anti-Inflammatory Activity
Source: Molecules. 2017 Jan 13;22(1):125. doi: 10.3390/molecules22010125 (PMC6155747; doi:10.3390/molecules22010125)
Supplement: Supplementary file 1 [file molecules-22-00125-s001.pdf]

Supplementary Materials: Chemical Constituents from the Roots and Rhizomes of *Asarum heterotropoides* var. *mandshuricum* and the In Vitro Anti-Inflammatory Activity

Yu Jing, Yi-Fan Zhang, Ming-Ying Shang, Guang-Xue Liu, Yao-Li Li, Xuan Wang and Shao-Qing Cai

1. Spectra of compounds 1–11

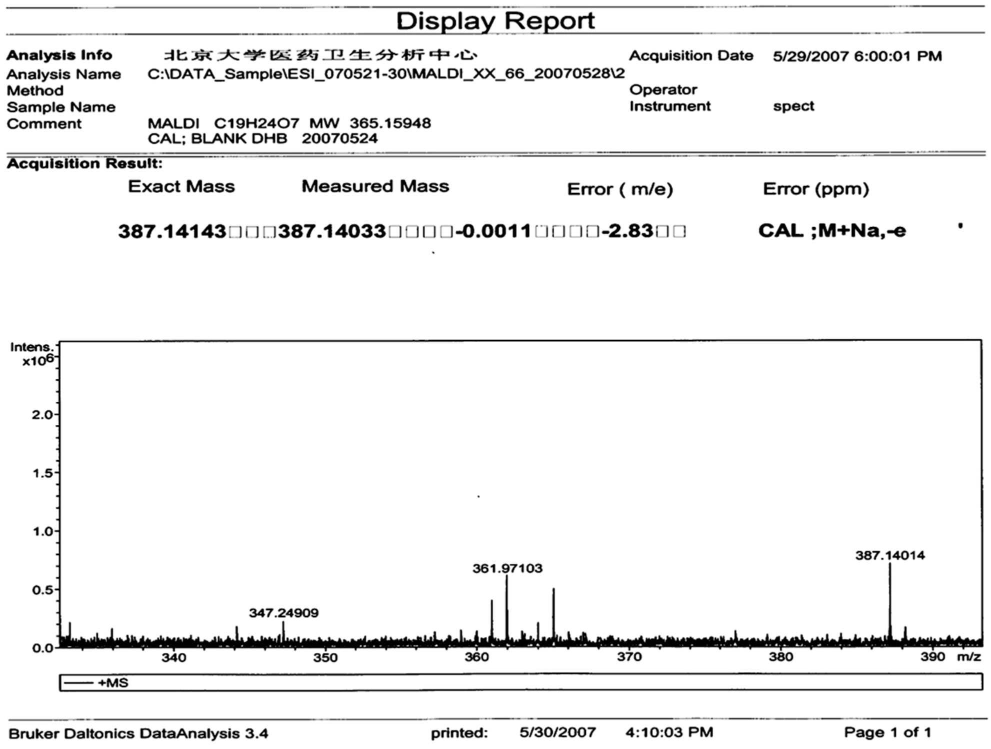

Figure S1. HRMALDIMS spectrum of compound **1**.

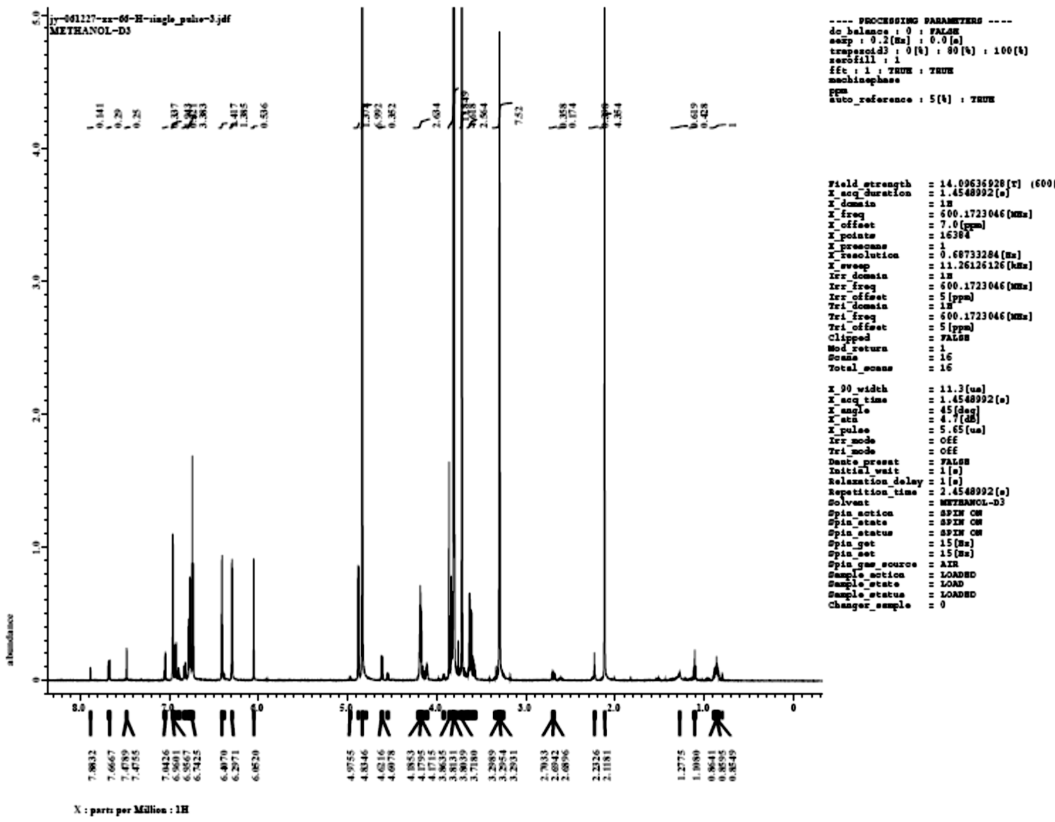

Figure S2. ^{1}H-NMR spectrum of compound **1** in CD_3OD.

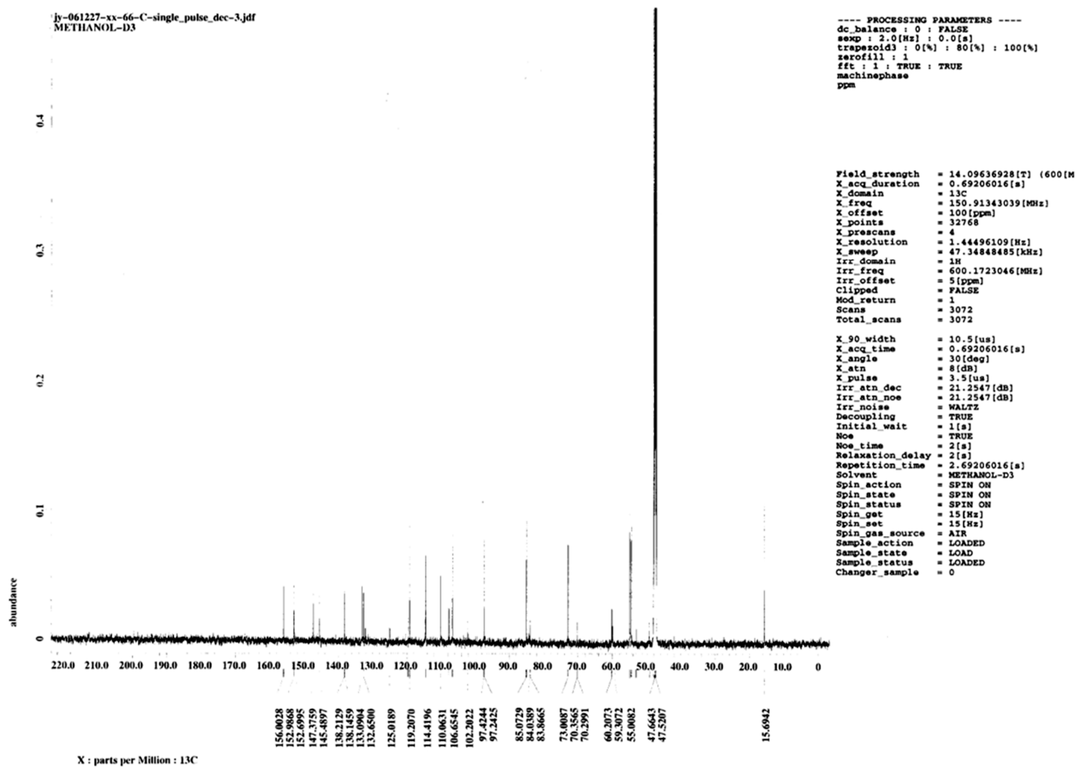

Figure S3. ^{13}C-NMR spectrum of compound **1** in CD_3OD.

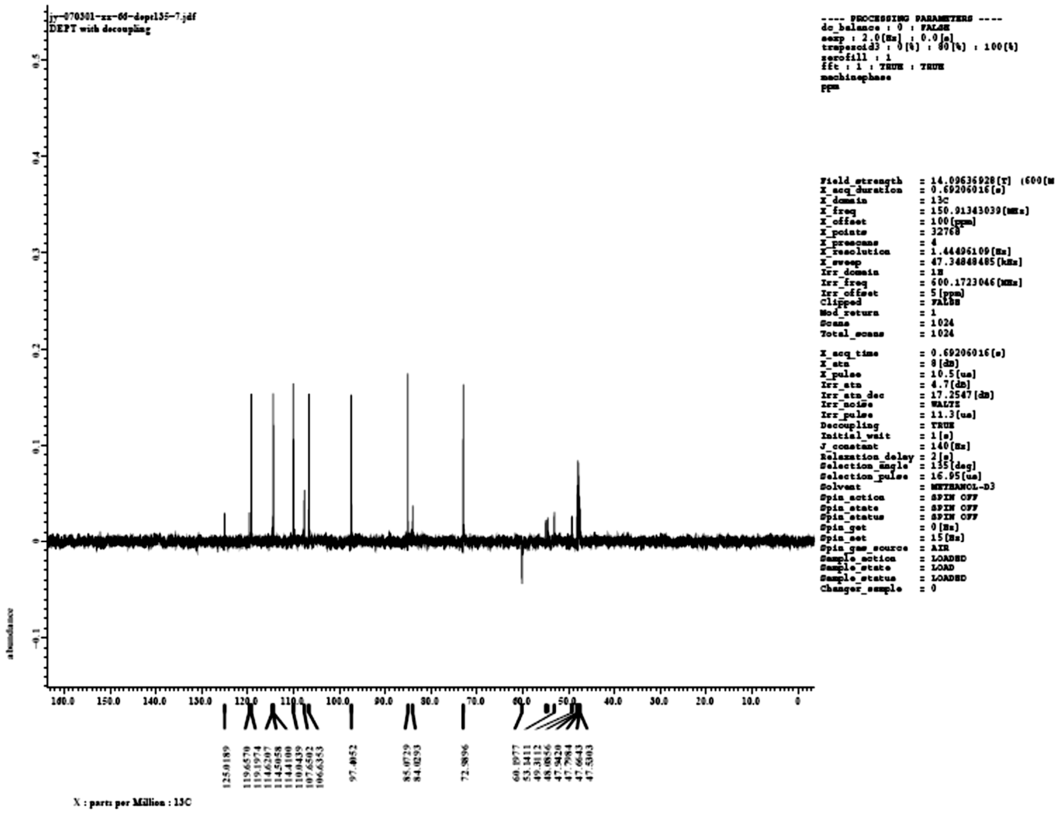

Figure S4. DEPT NMR spectrum of compound **1** in CD_3OD.

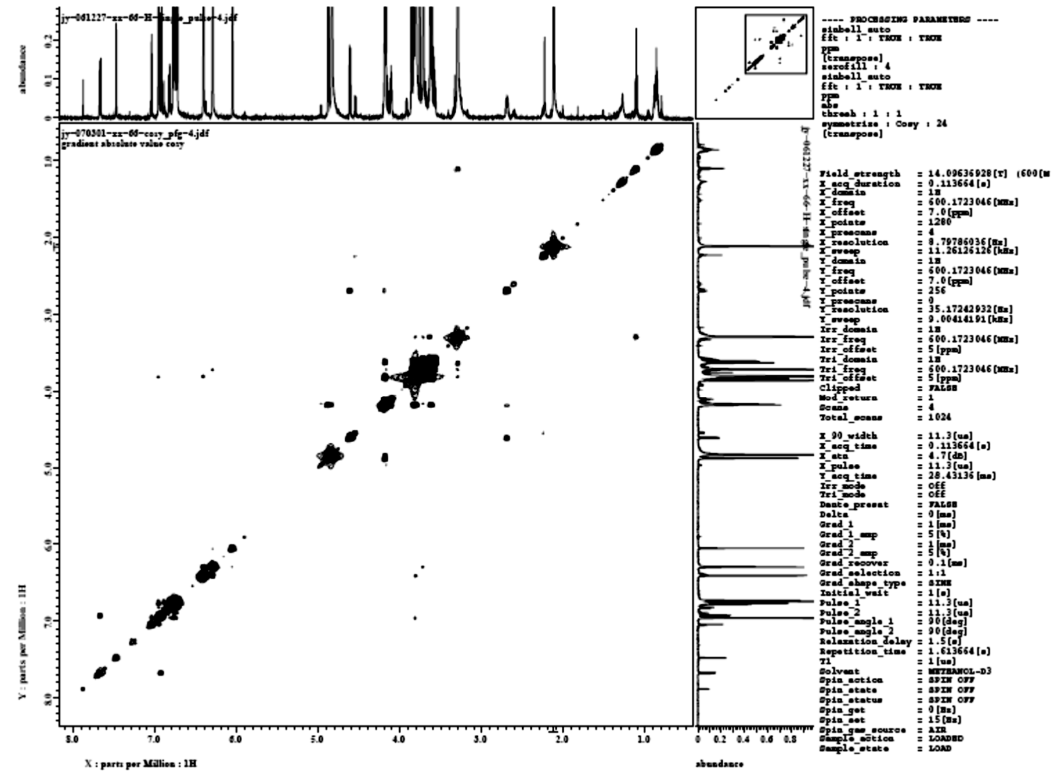

Figure S5. ^1H–^1H COSY NMR spectrum of compound **1** in CD_3OD.

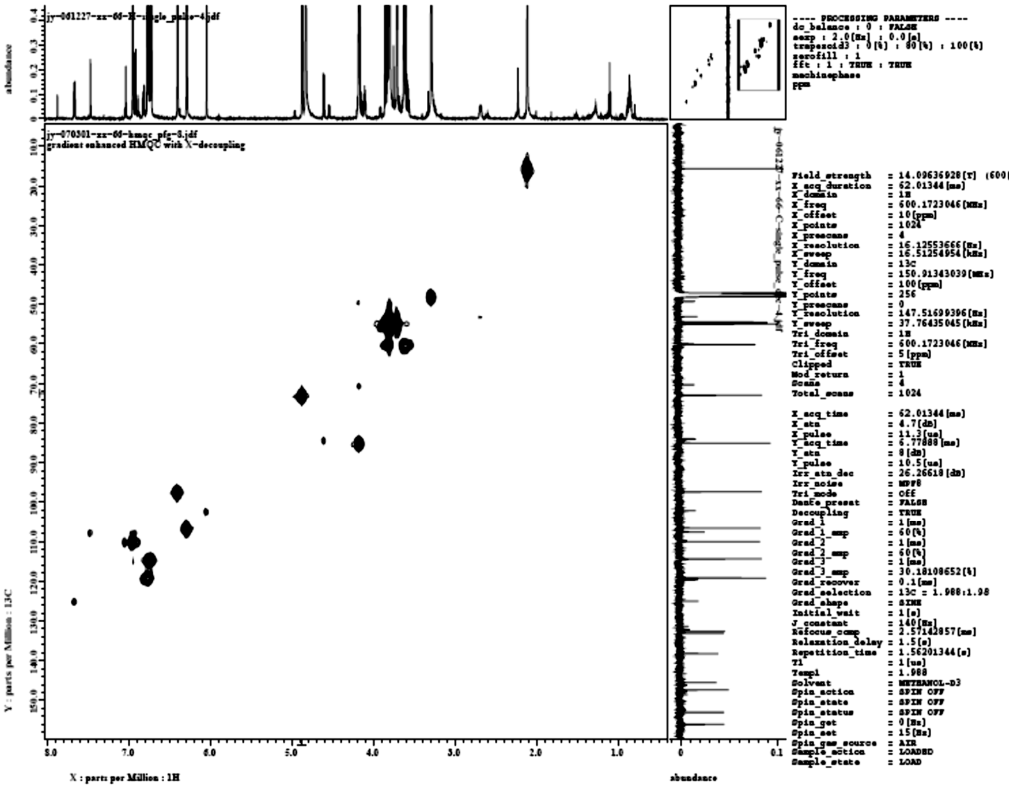

Figure S6. HMQC-NMR spectrum of compound **1** in CD_3OD.

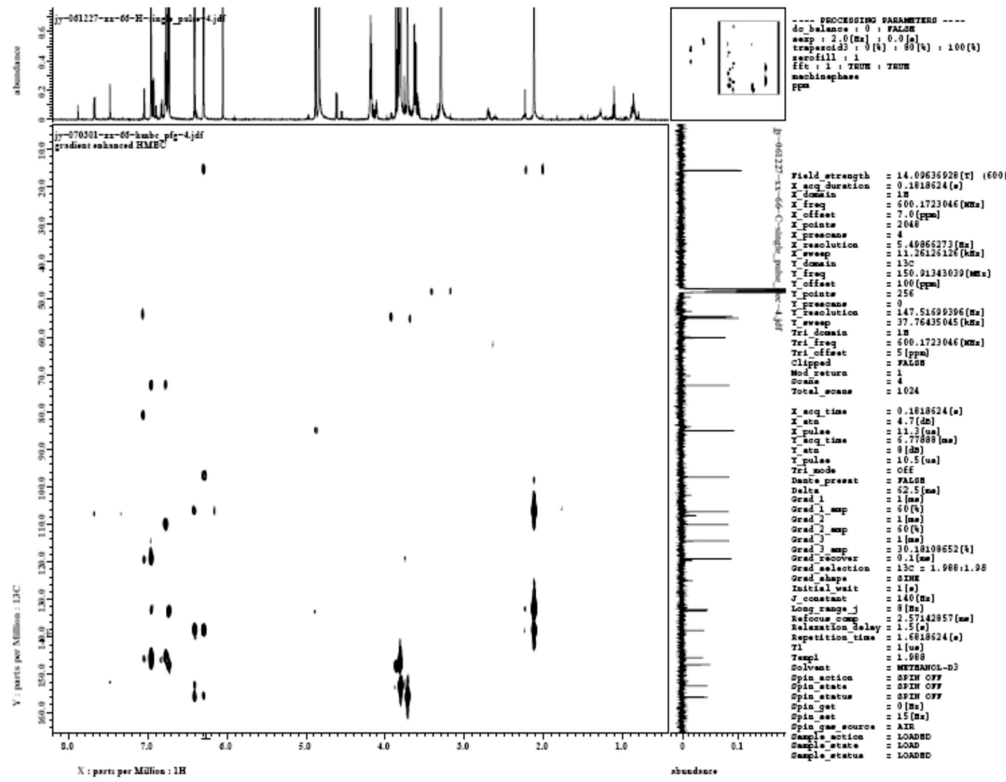

Figure S7. HMBC-NMR spectrum of compound **1** in CD_3OD.

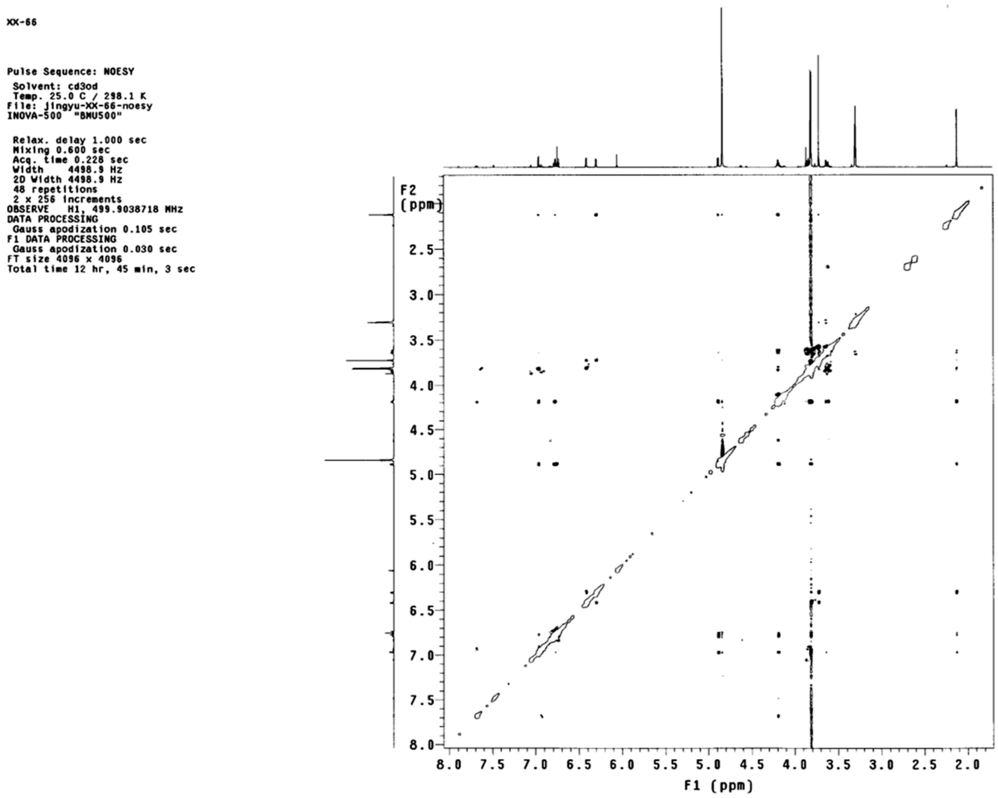

Figure S8. NOESY NMR spectrum of compound **1** in CD_3OD.

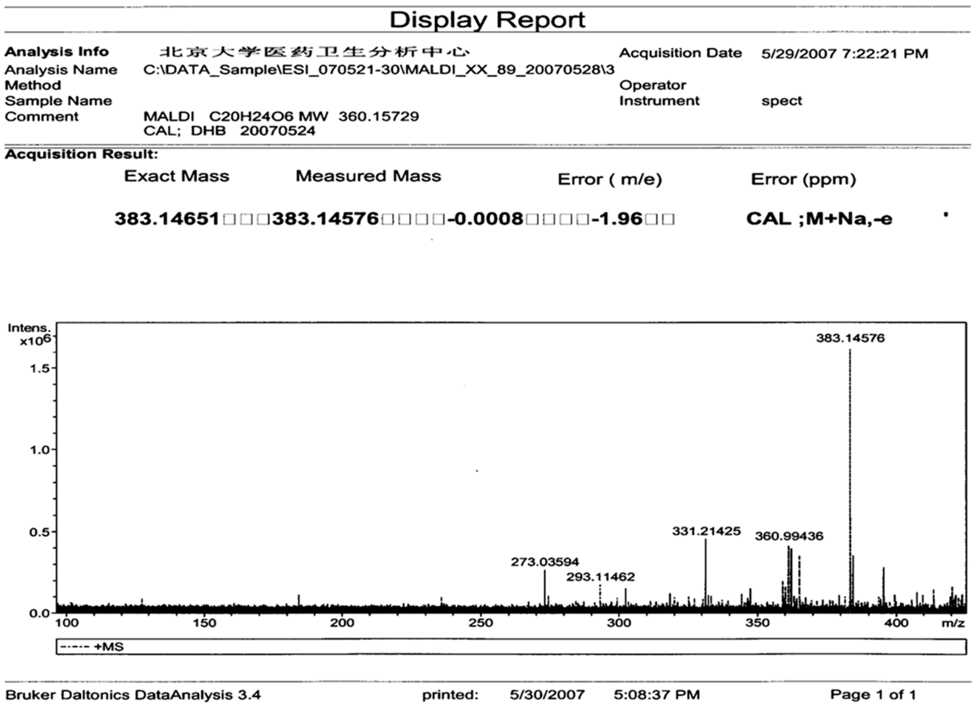

Figure S9. HRMALDIMS spectrum of compound **2**.

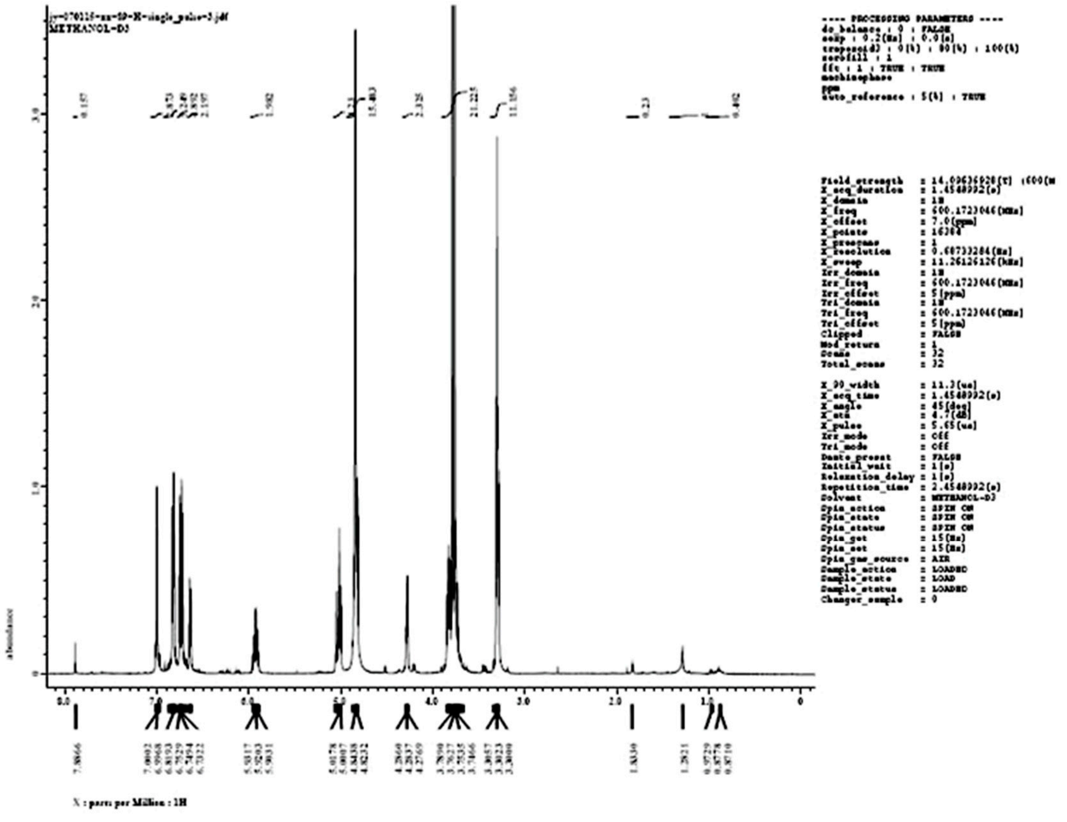

Figure S10. ^{1}H-NMR spectrum of compound **2** in CD_3OD.

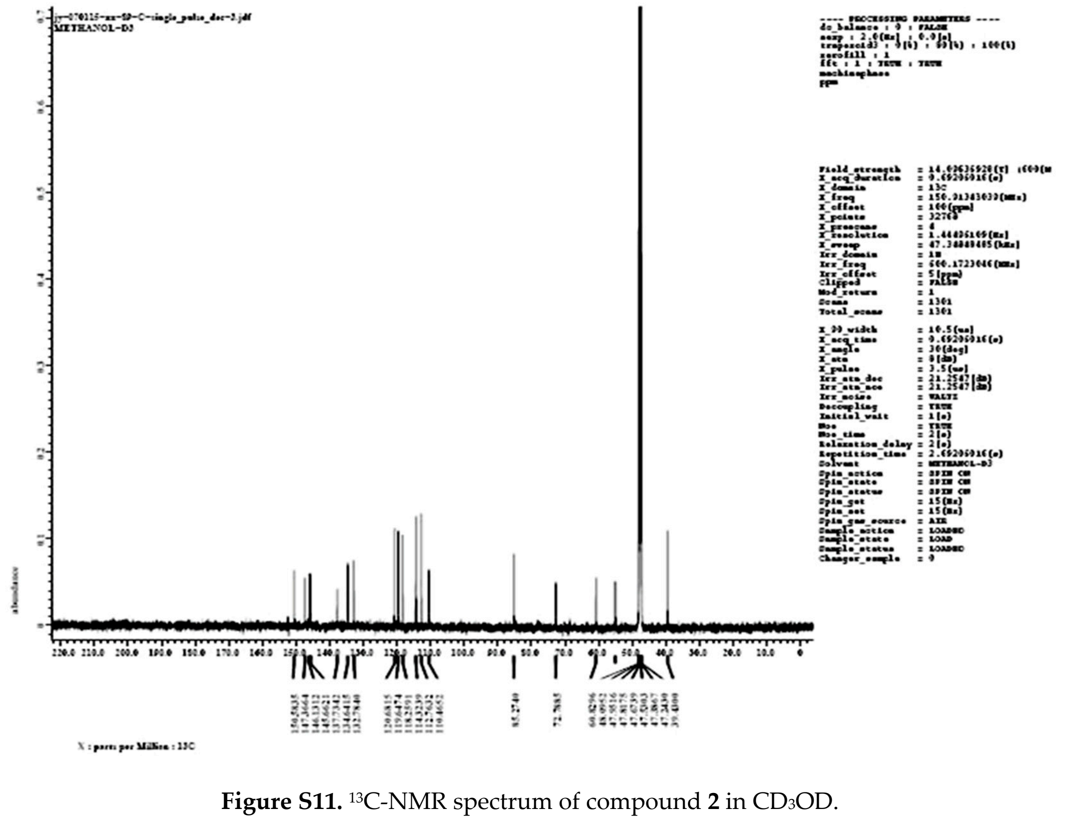

Figure S11. ^{13}C-NMR spectrum of compound **2** in CD_3OD.

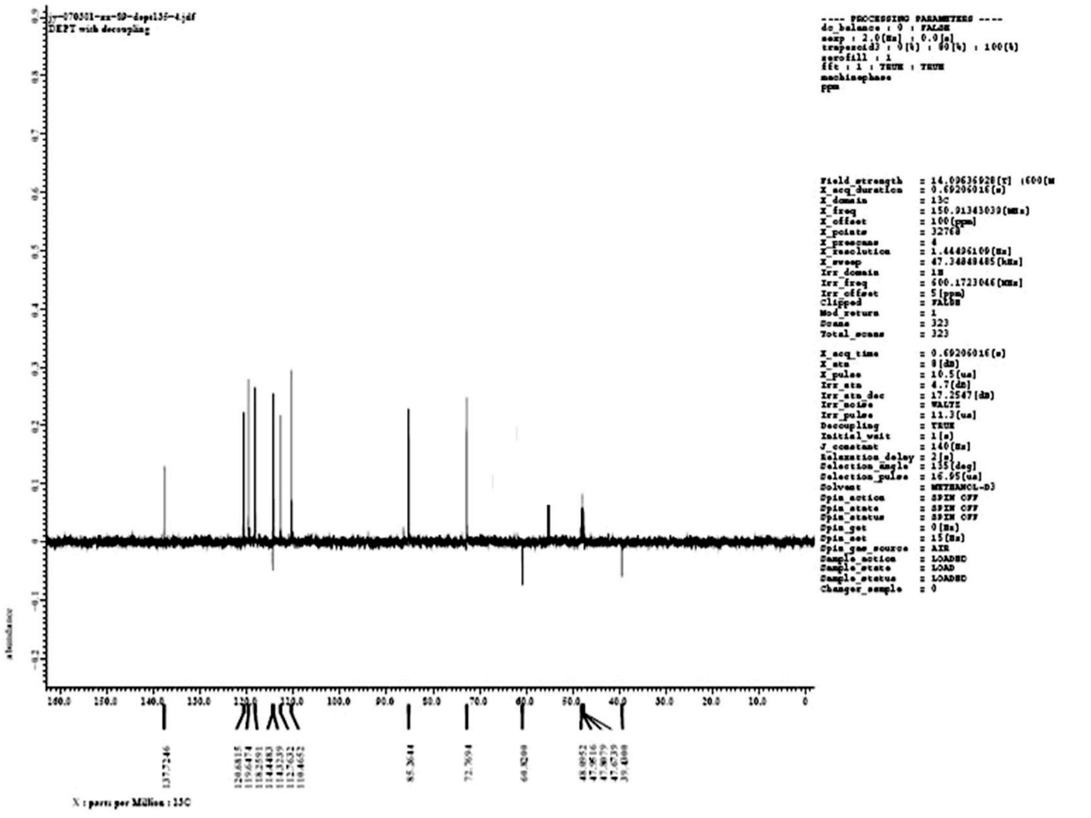

Figure S12 DEPT NMR spectrum of compound **2** in CD_3OD.

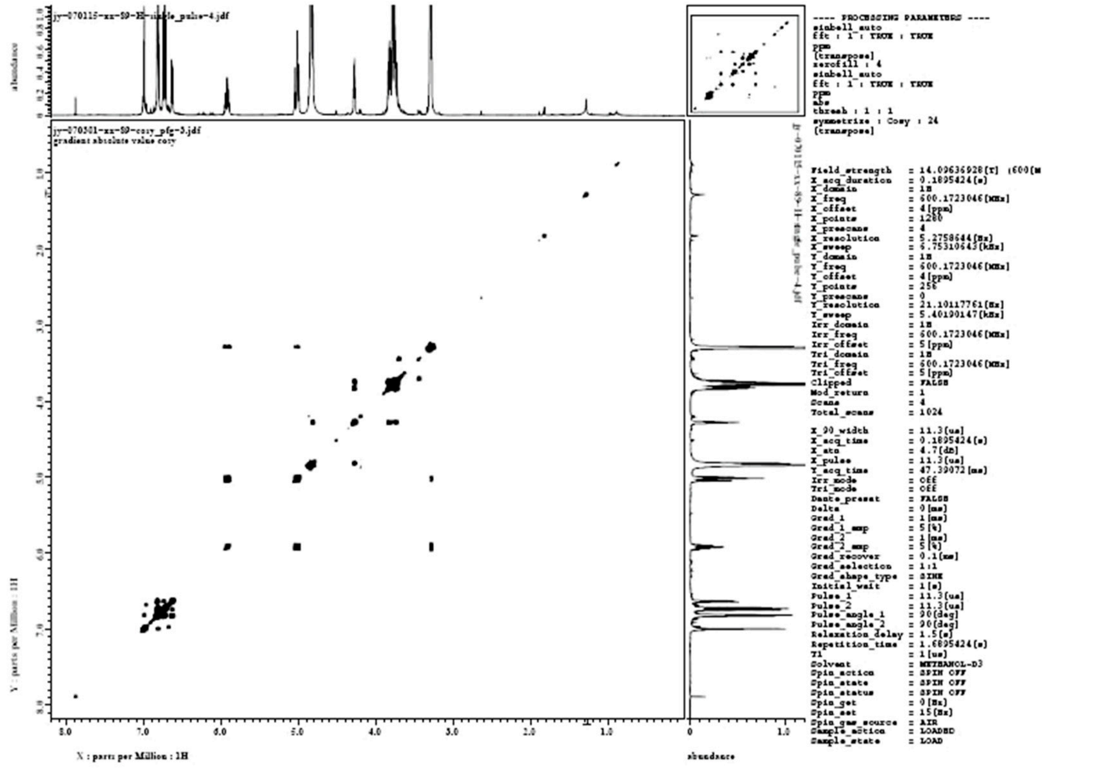

Figure S13. ^{1}H–^{1}H COSY NMR spectrum of compound **2** in CD_3OD.

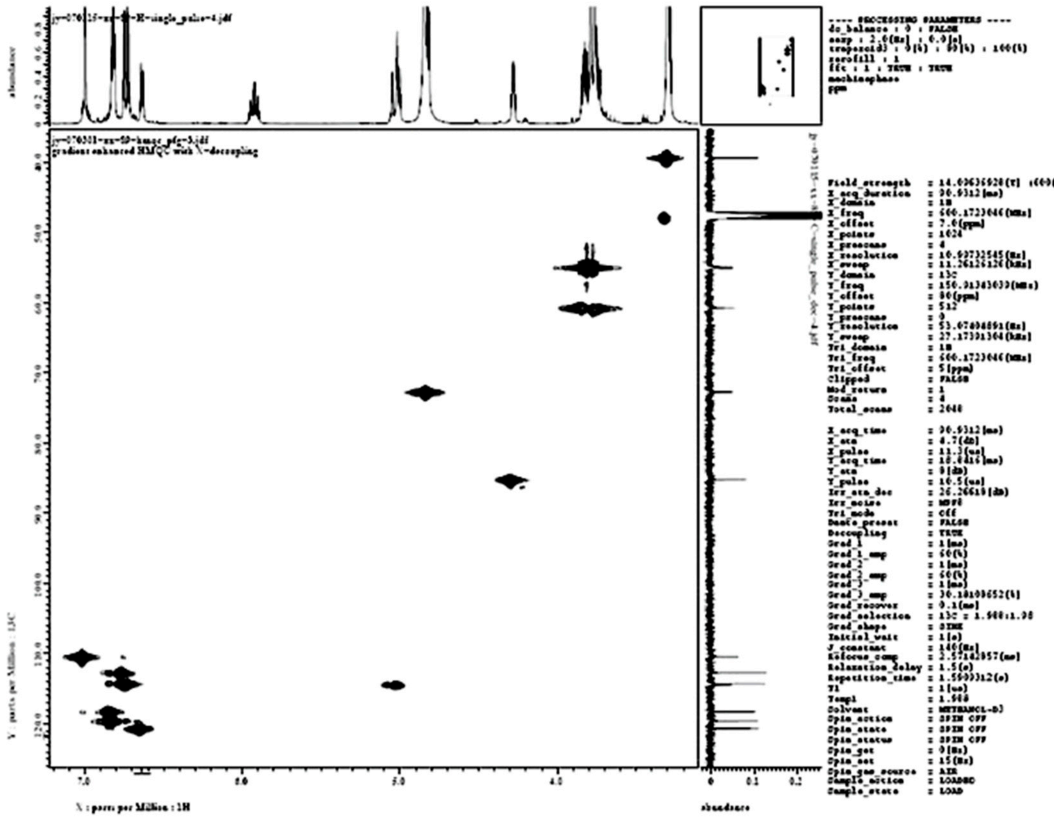

Figure S14. HMQC-NMR spectrum of compound **2** in CD_3OD.

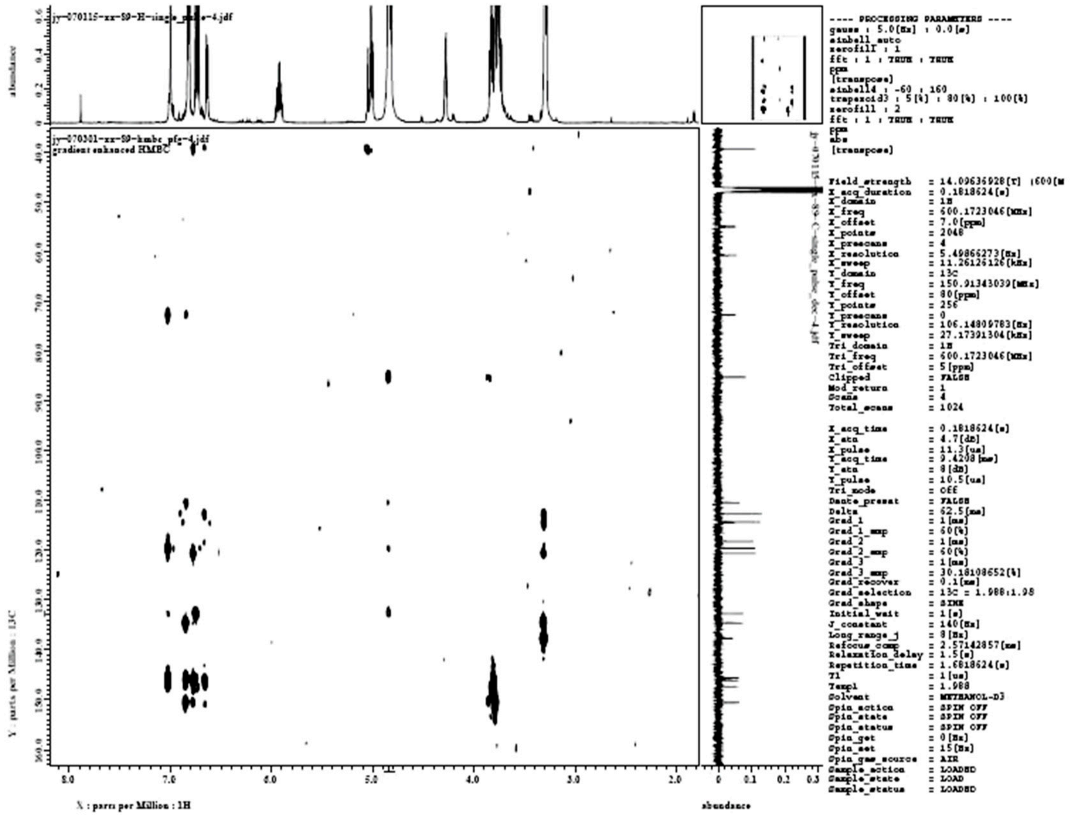

Figure S15. HMBC-NMR spectrum of compound **2** in CD_3OD.

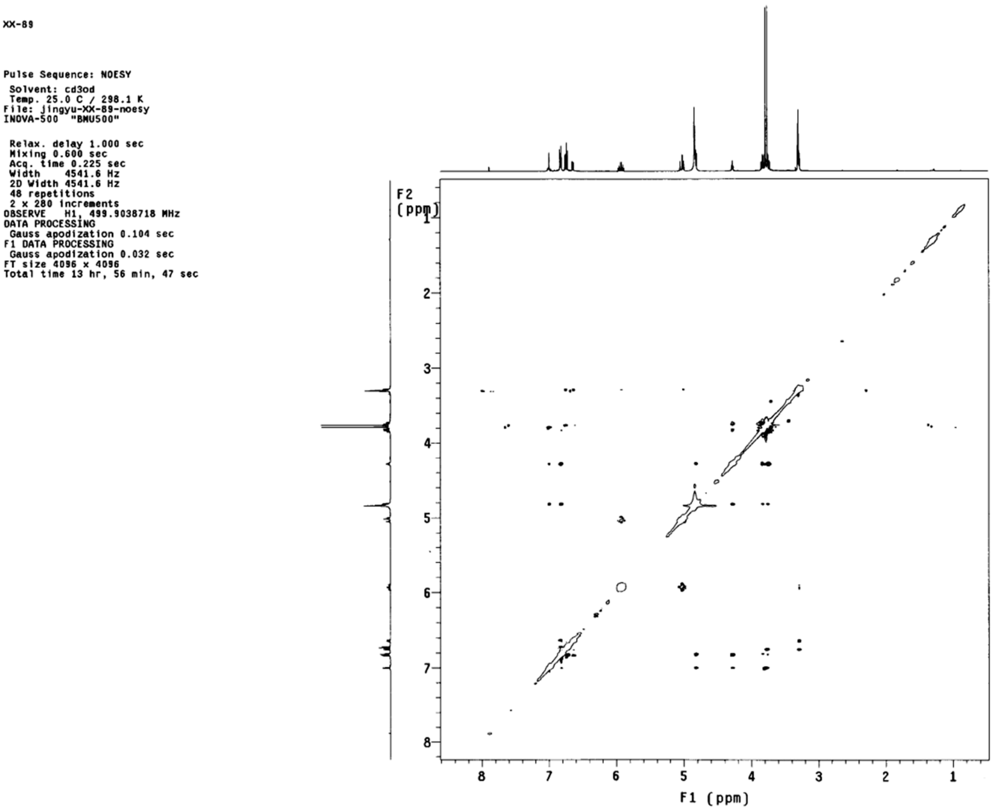

Figure S16. NOESY NMR spectrum of compound **2** in CD_3OD.

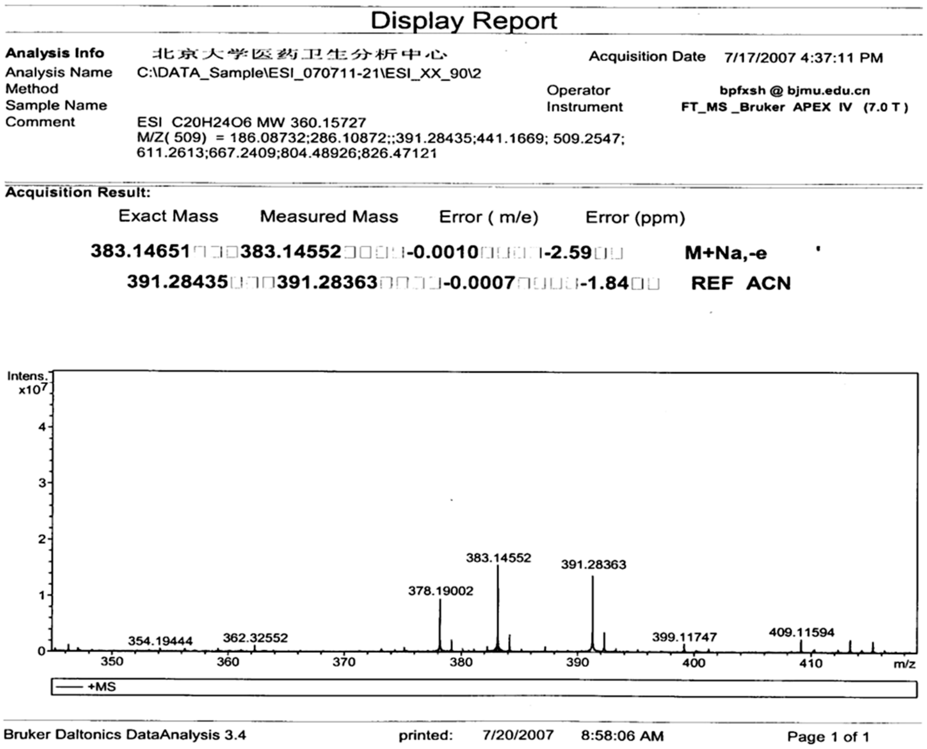

Figure S17. HRESIMS spectrum of compound **3**.

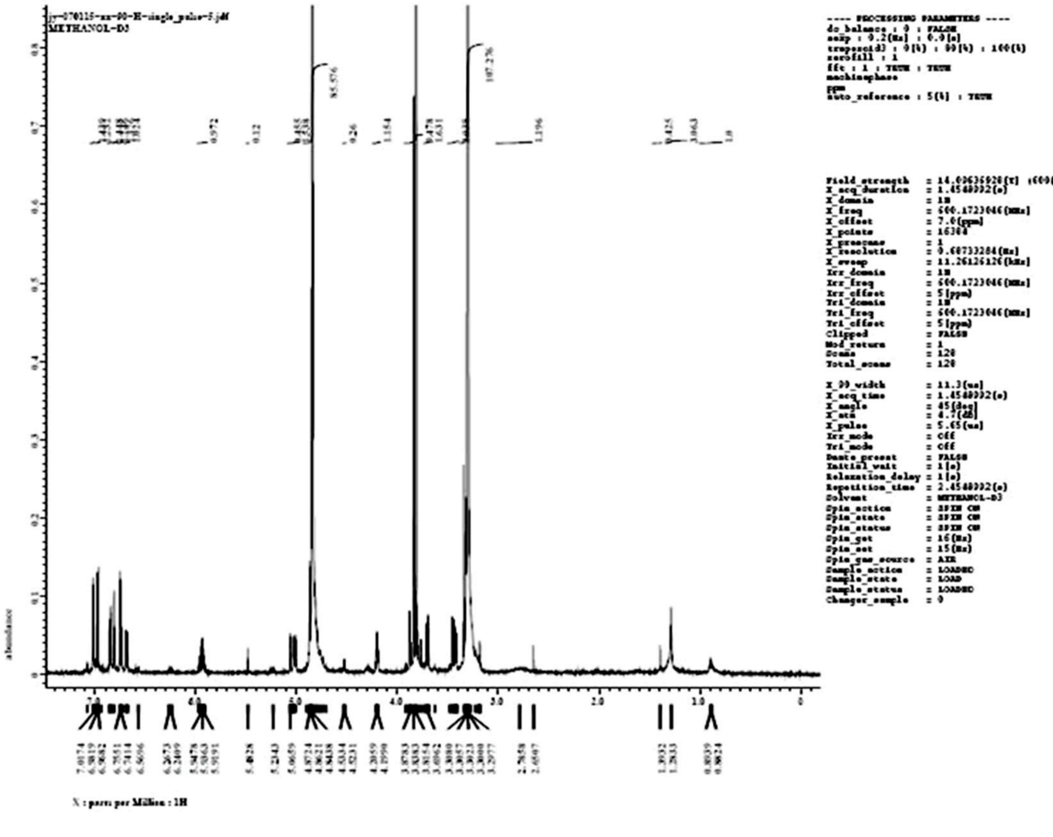

Figure S18. ^{1}H-NMR spectrum of compound **3** in CD_3OD.

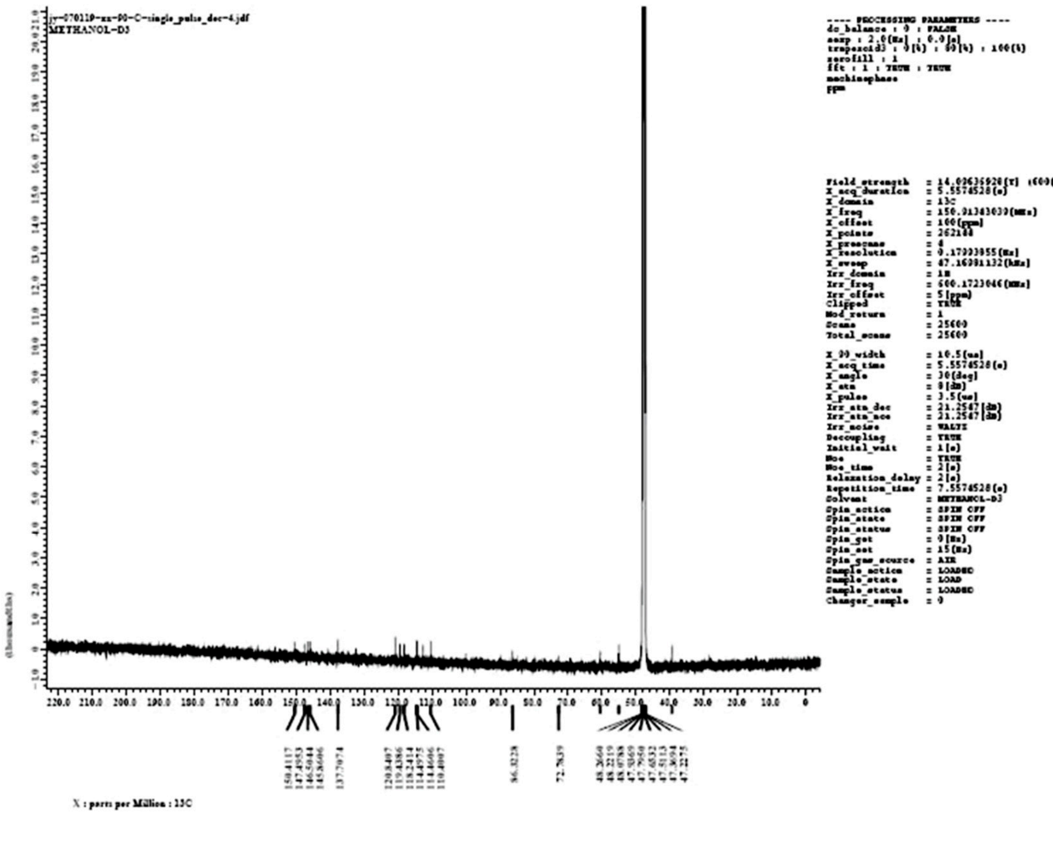

Figure S19. ^{13}C-NMR spectrum of compound **3** in CD_3OD.

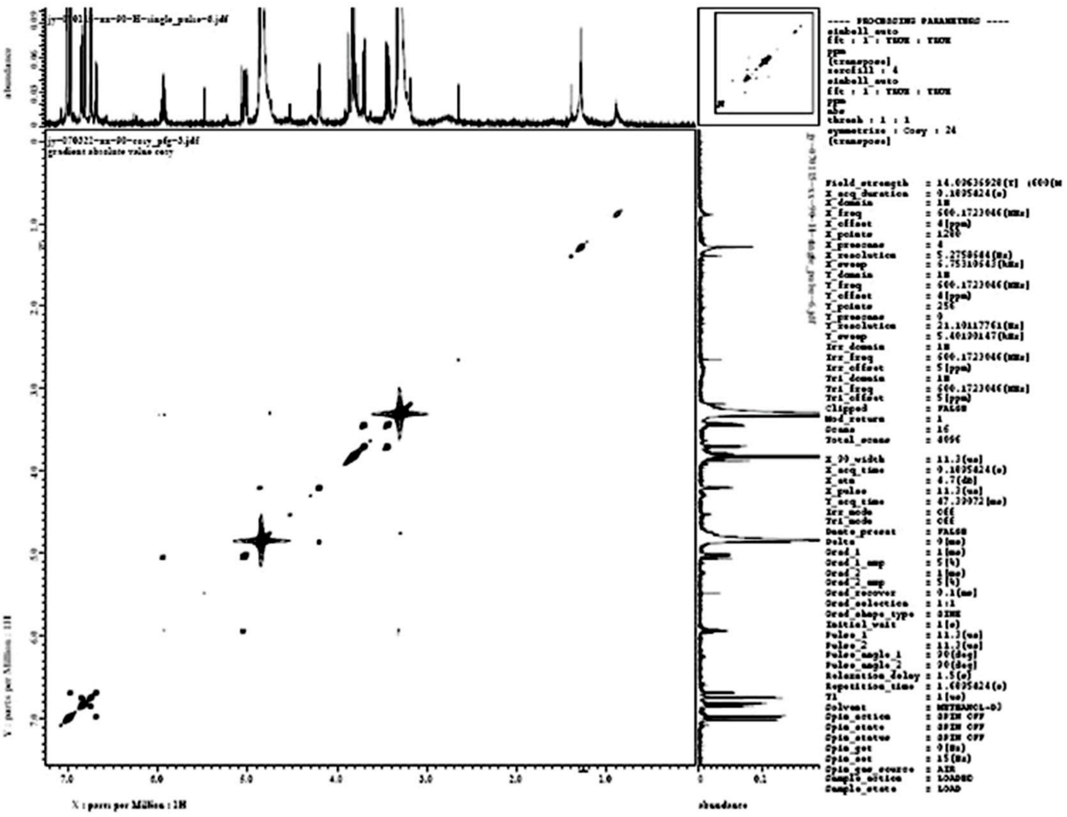

Figure S20. ^{1}H–^{1}H COSY NMR spectrum of compound **3** in CD_3OD.

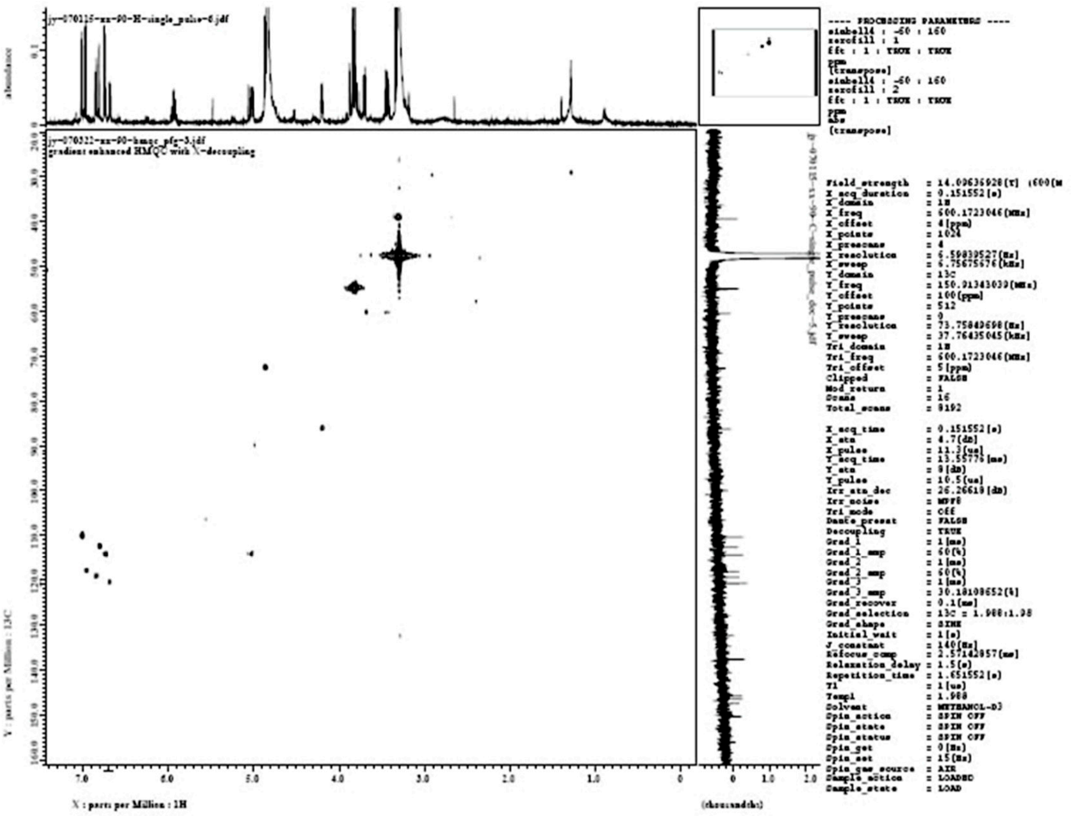

Figure S21. HMQC-NMR spectrum of compound **3** in CD_3OD.

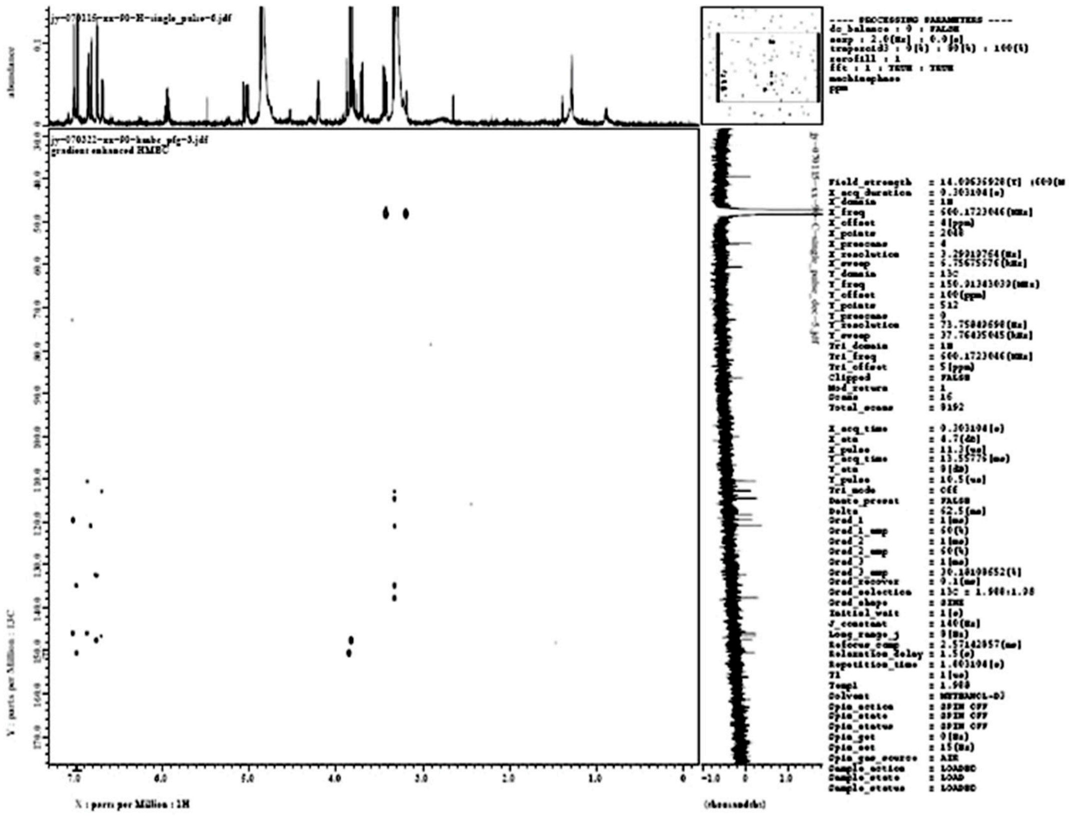

Figure S22. HMBC-NMR spectrum of compound **3** in CD_3OD.

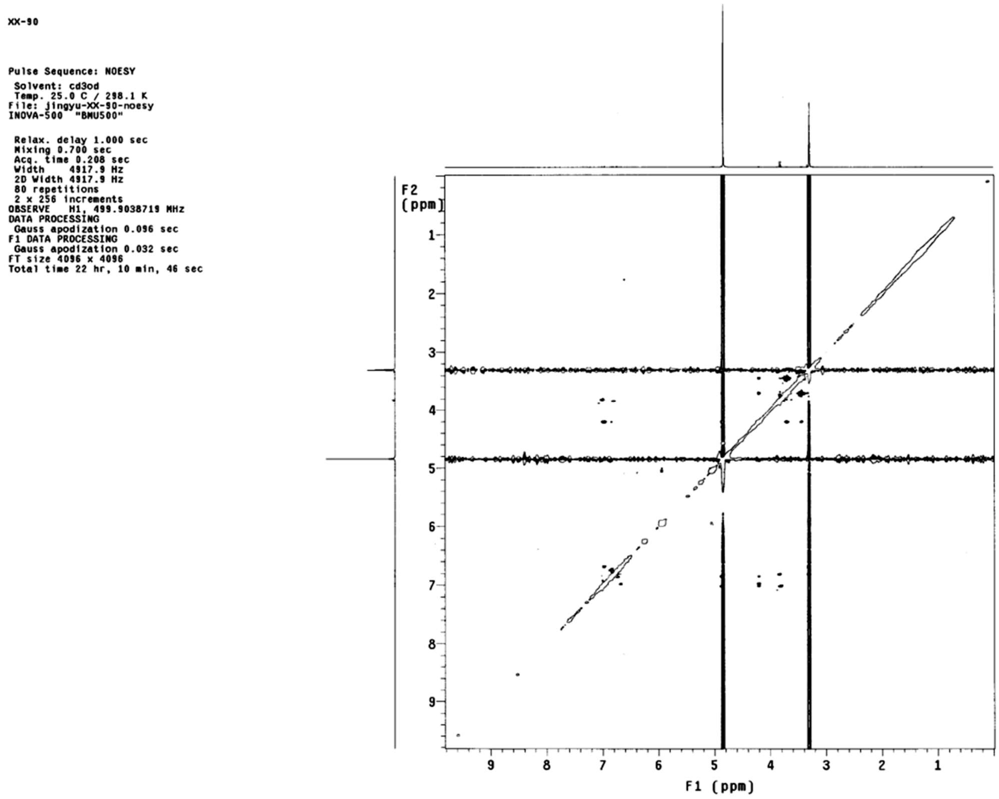

Figure S23. NOESY NMR spectrum of compound **3** in CD_3OD.

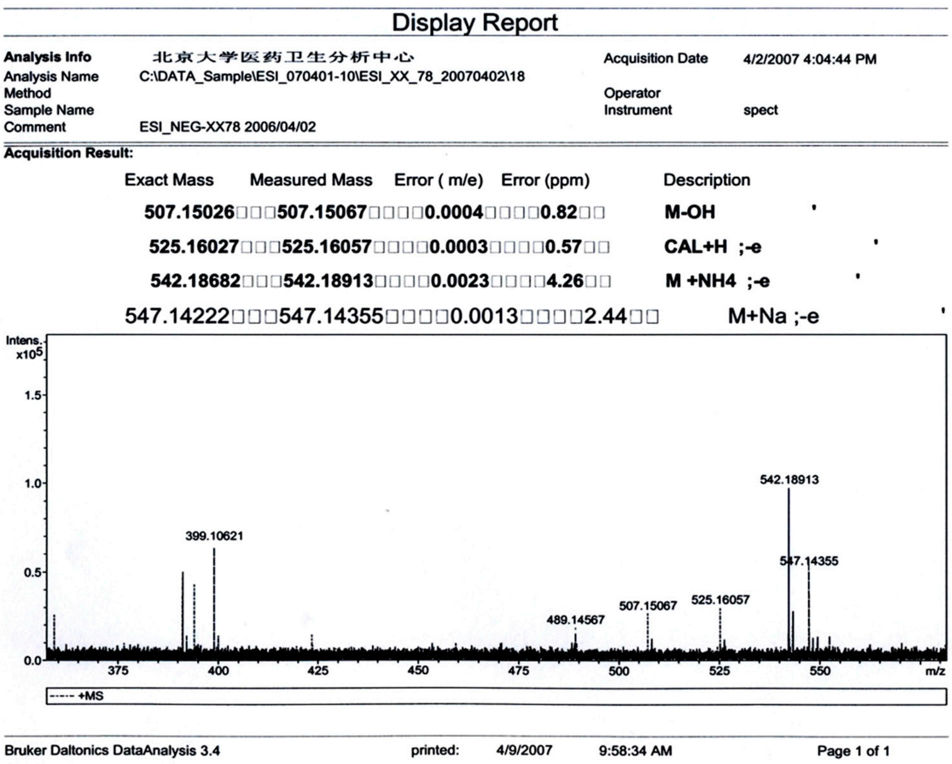

Figure S24. HRESIMS spectrum of compound **4**.

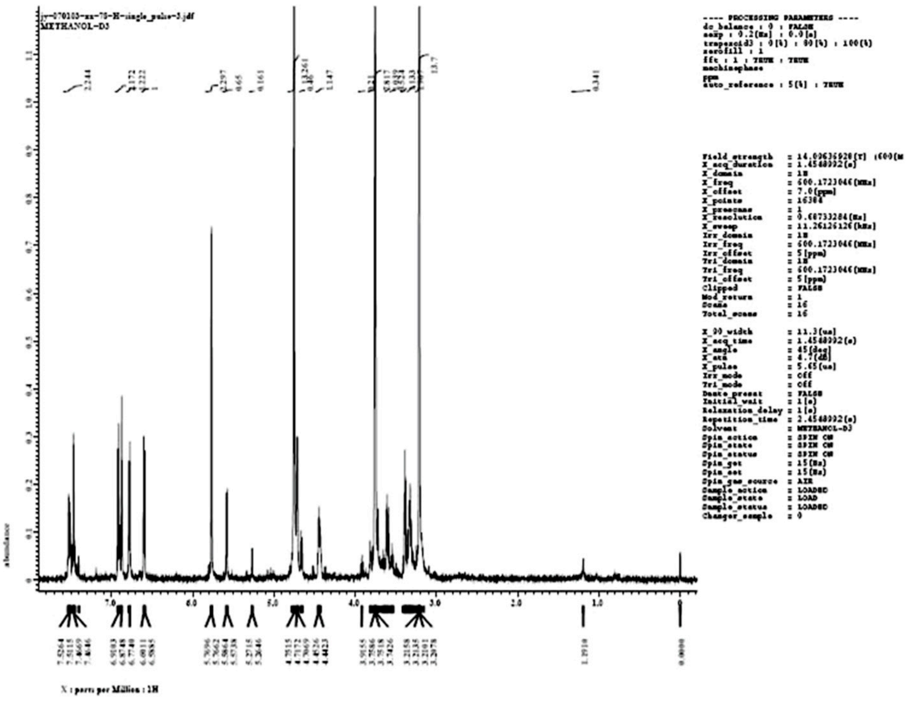

Figure S25. ^{1}H-NMR spectrum of compound **4** in CD_3OD.

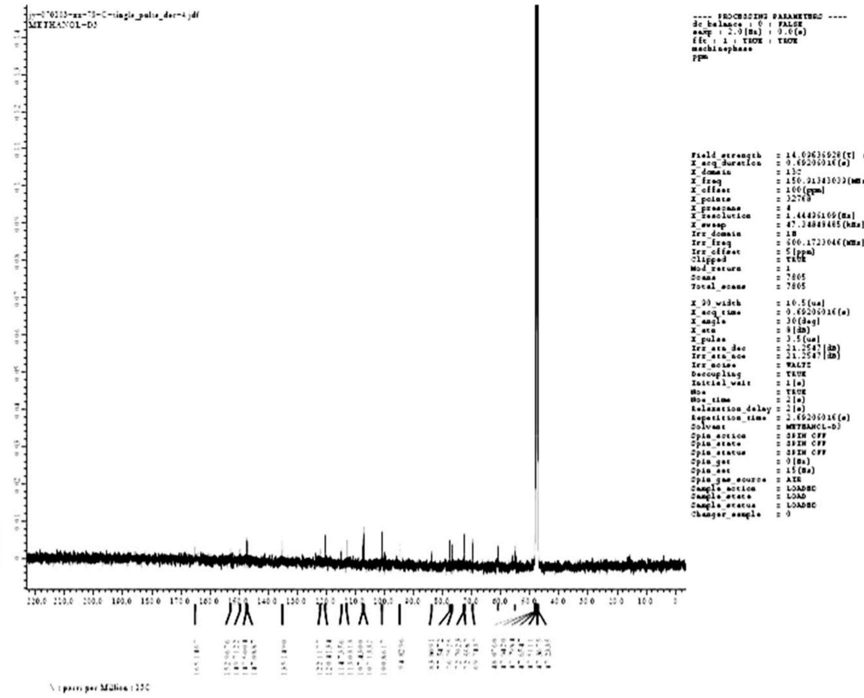

Figure S26. ^{13}C-NMR spectrum of compound **4** in CD_3OD.

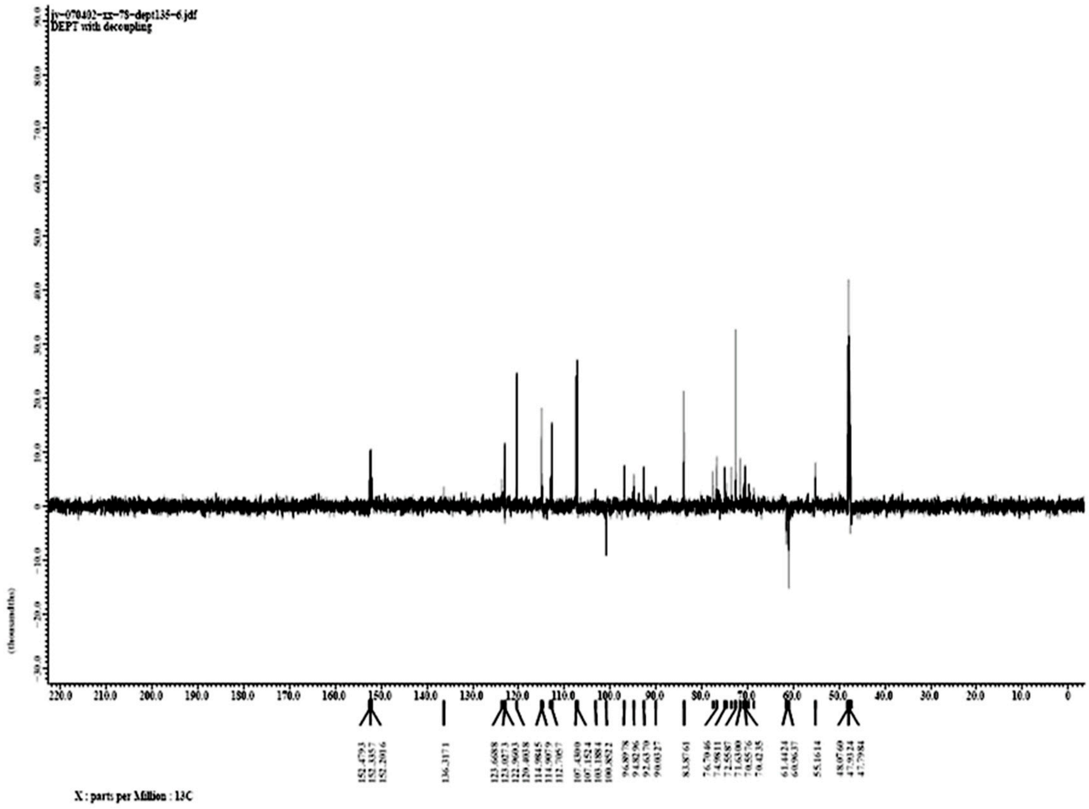

Figure S27. DEPT NMR spectrum of compound **4** in CD_3OD.

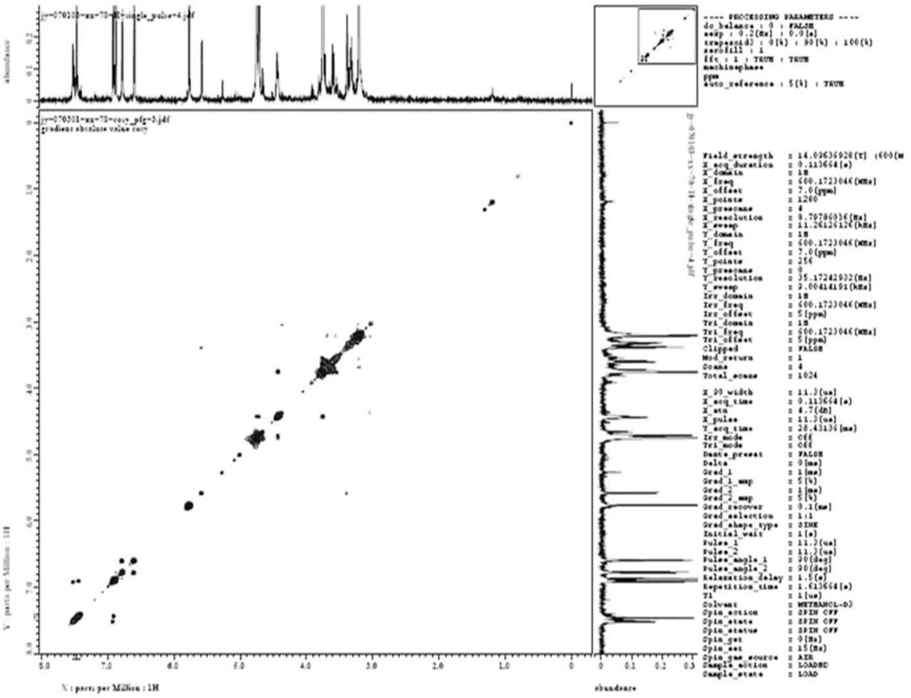

Figure S28.^{1}H–^{1}H COSY NMR spectrum of compound **4** in CD_3OD.

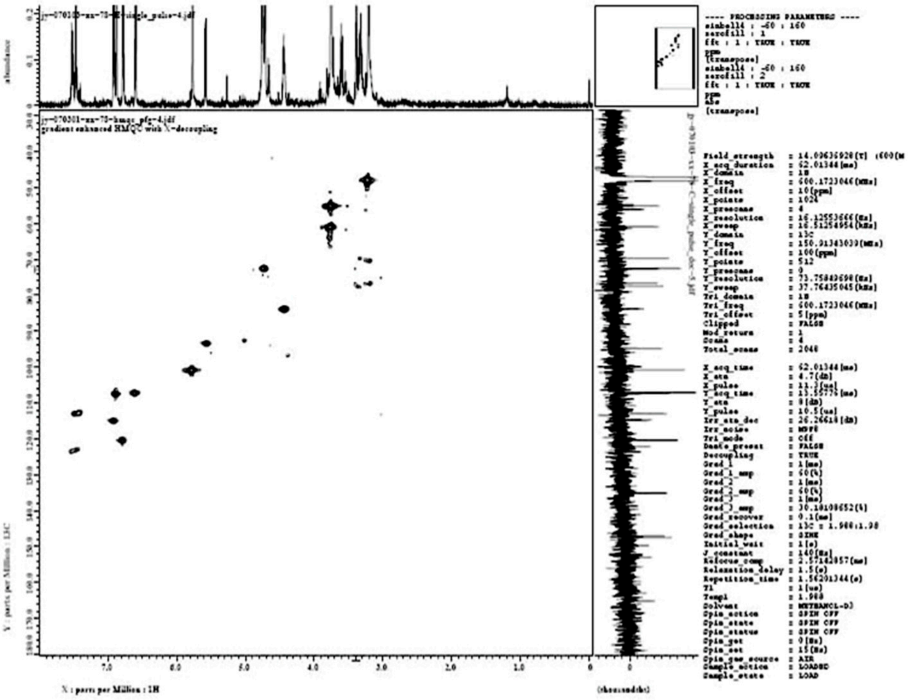

Figure S29. HMQC-NMR spectrum of compound **4** in CD_3OD.

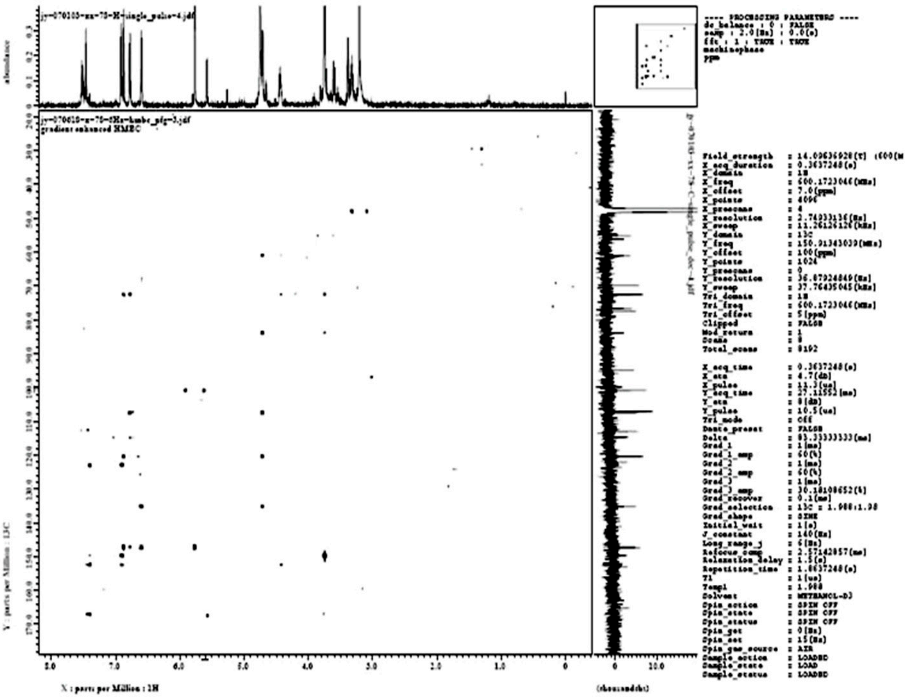

Figure S30. HMBC-NMR spectrum of compound **4** in CD_3OD.

Display Report

**Analysis Info**	北京大学医药卫生分析中心	Acquisition Date	4/2/2007 3:51:12 PM
Analysis Name	C:\DATA_Sample\ESI_070401-10\ESI_XX_70_20070402\10		
Method		Operator	
Sample Name		Instrument	spect
Comment	ESI_NEG-XX70 2006/04/02		

Acquisition Result:

Exact Mass	Measured Mass	Error (m/e)	Error (ppm)	Description
**551.17702□□□**	**551.17761□□□□**	**0.0006□□□□**	**1.07□□**	**-H;+e**

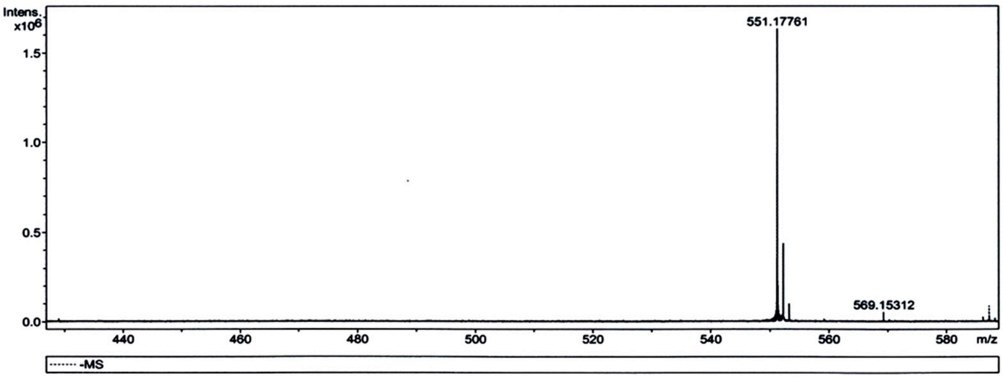

Bruker Daltonics DataAnalysis 3.4 printed: 4/9/2007 9:49:30 AM Page 1 of 1

Figure S31. HRESIMS spectrum of compound **5**.

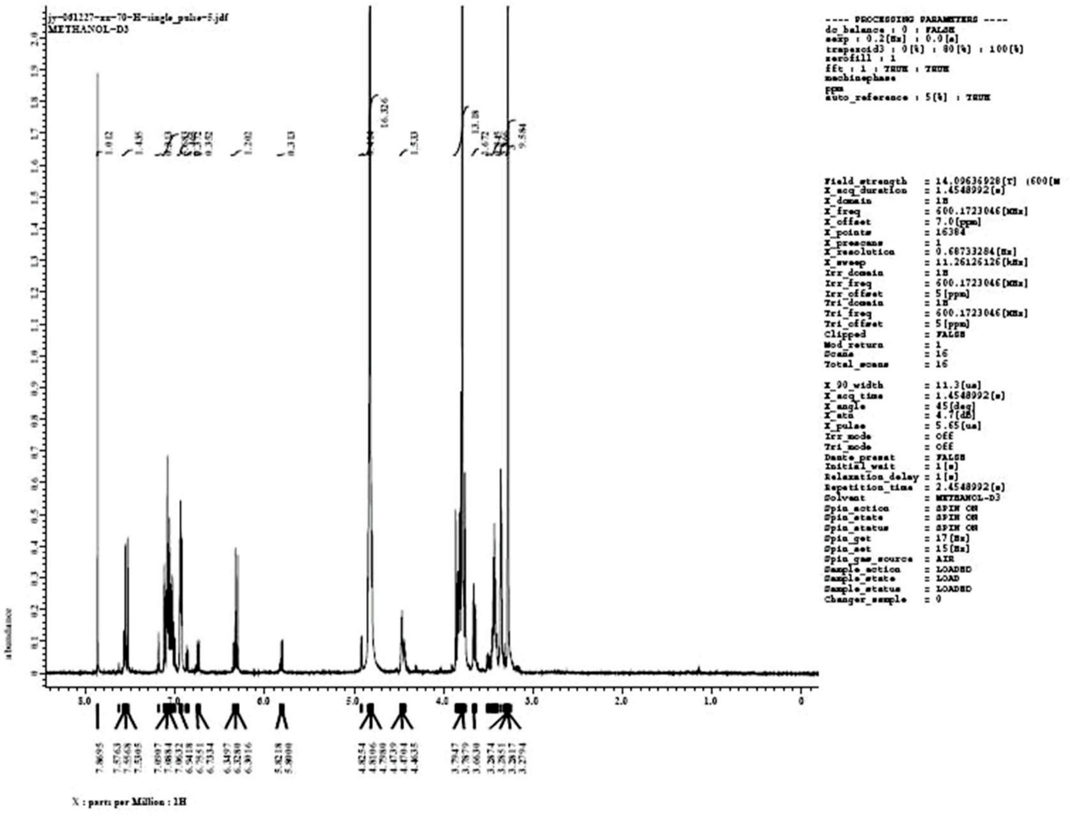

Figure S32. ^{1}H-NMR spectrum of compound **5** in CD_3OD.

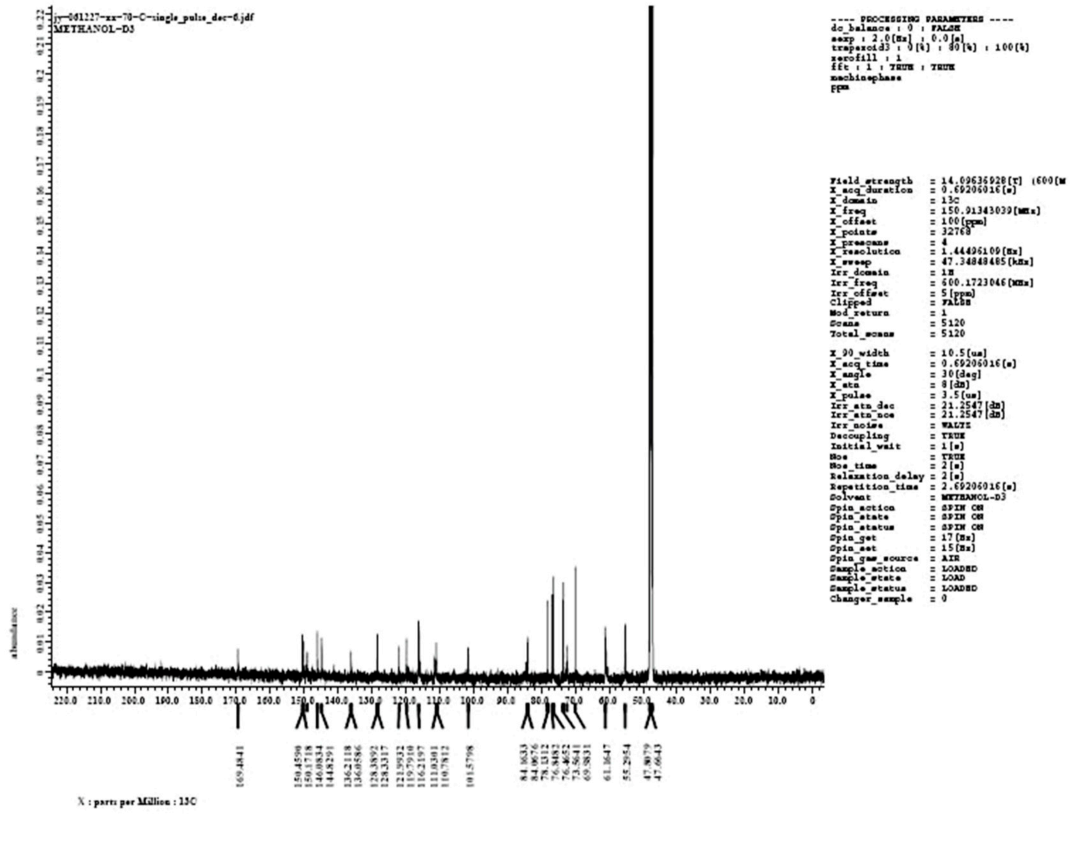

Figure S33. ^{13}C-NMR spectrum of compound **5** in CD_3OD.

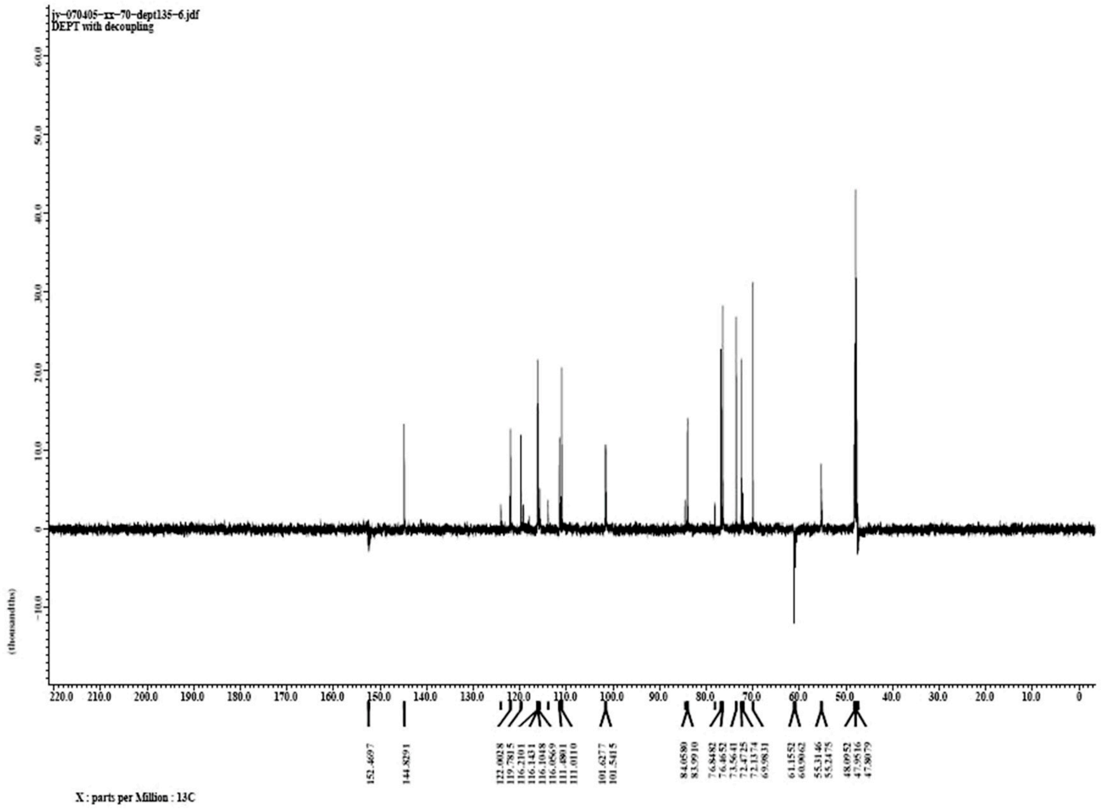

Figure S34. DEPT NMR spectrum of compound **5** in CD_3OD.

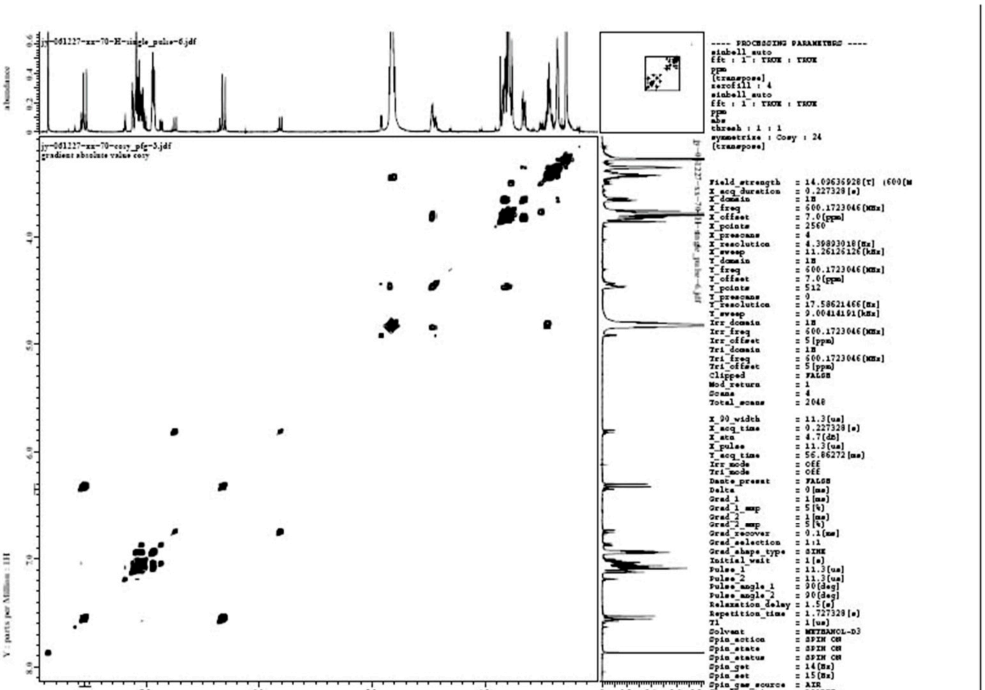

Figure S35.^1H–^1H COSY NMR spectrum of compound **5** in CD_3OD.

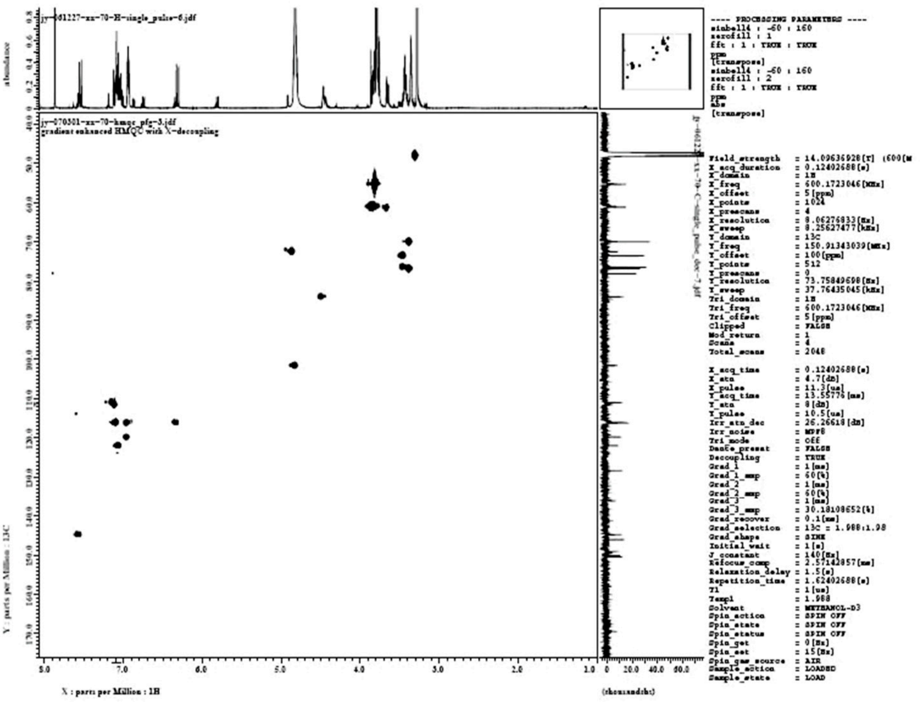

Figure S36. HMQC-NMR spectrum of compound **5** in CD_3OD.

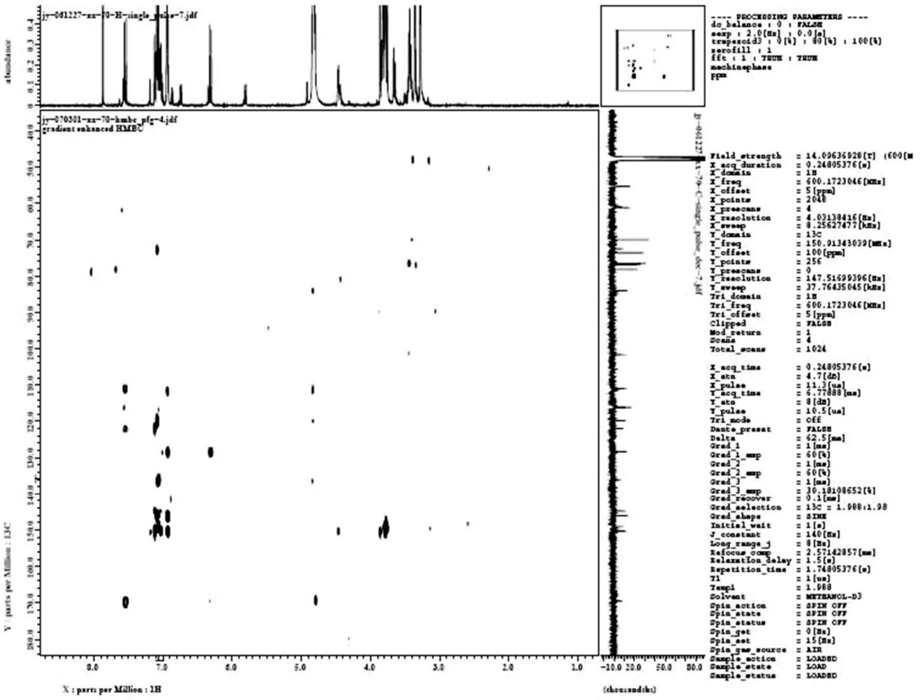

Figure S37. HMBC-NMR spectrum of compound **5** in CD_3OD.

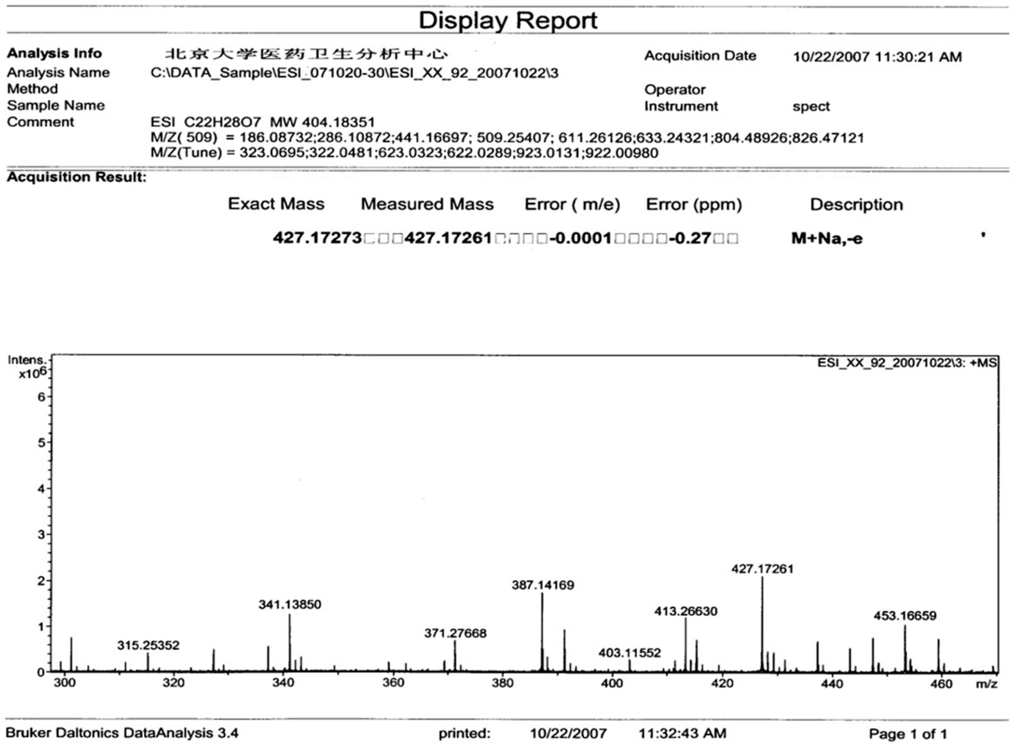

Figure S38. HRESIMS spectrum of compound **6**.

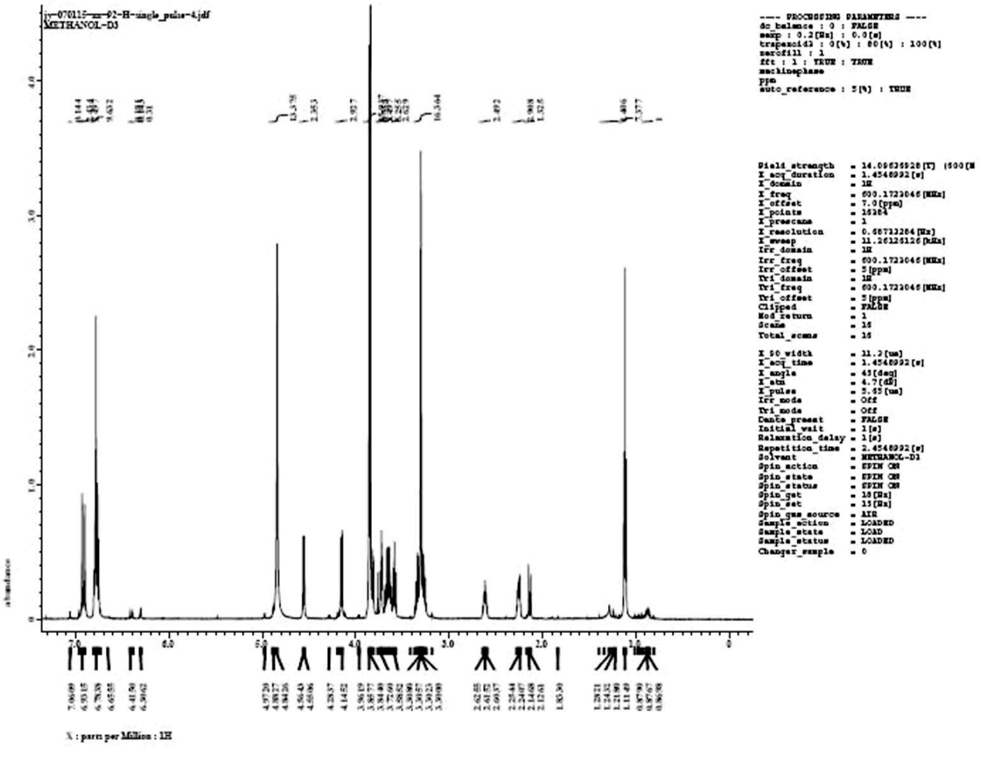

Figure S39. ^{1}H-NMR spectrum of compound **6** in CD_3OD.

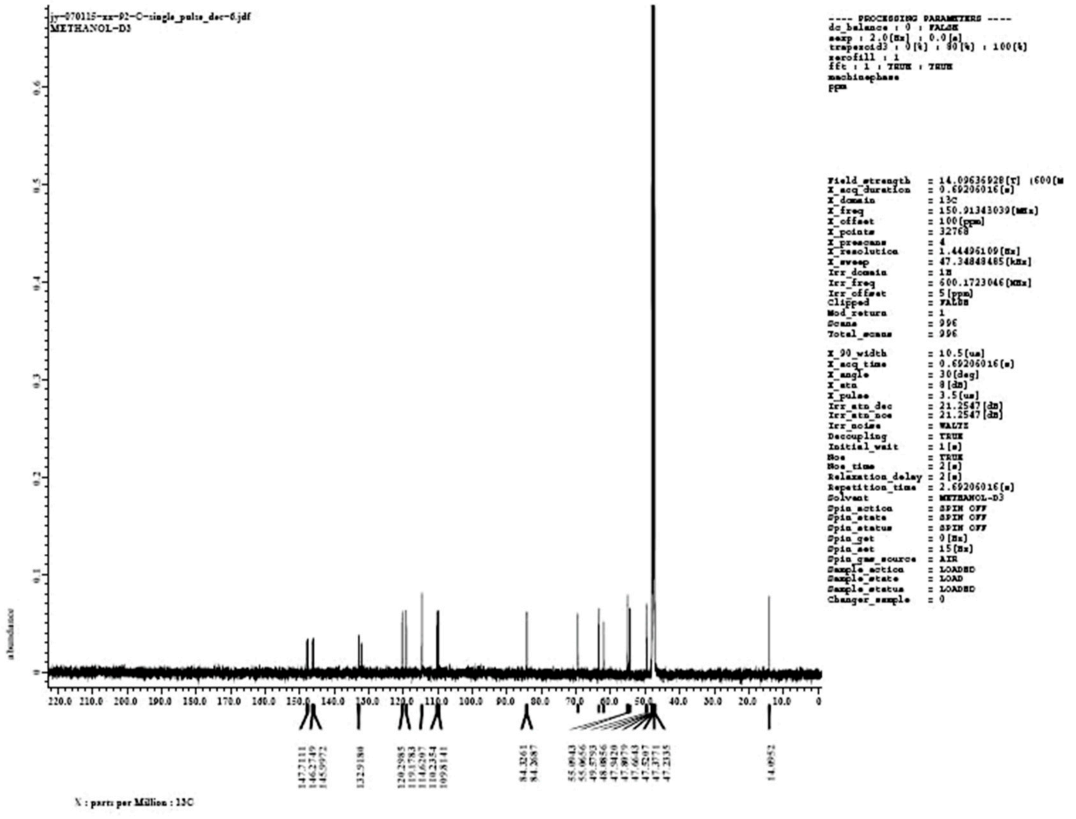

Figure S40. ^{13}C-NMR spectrum of compound **6** in CD_3OD.

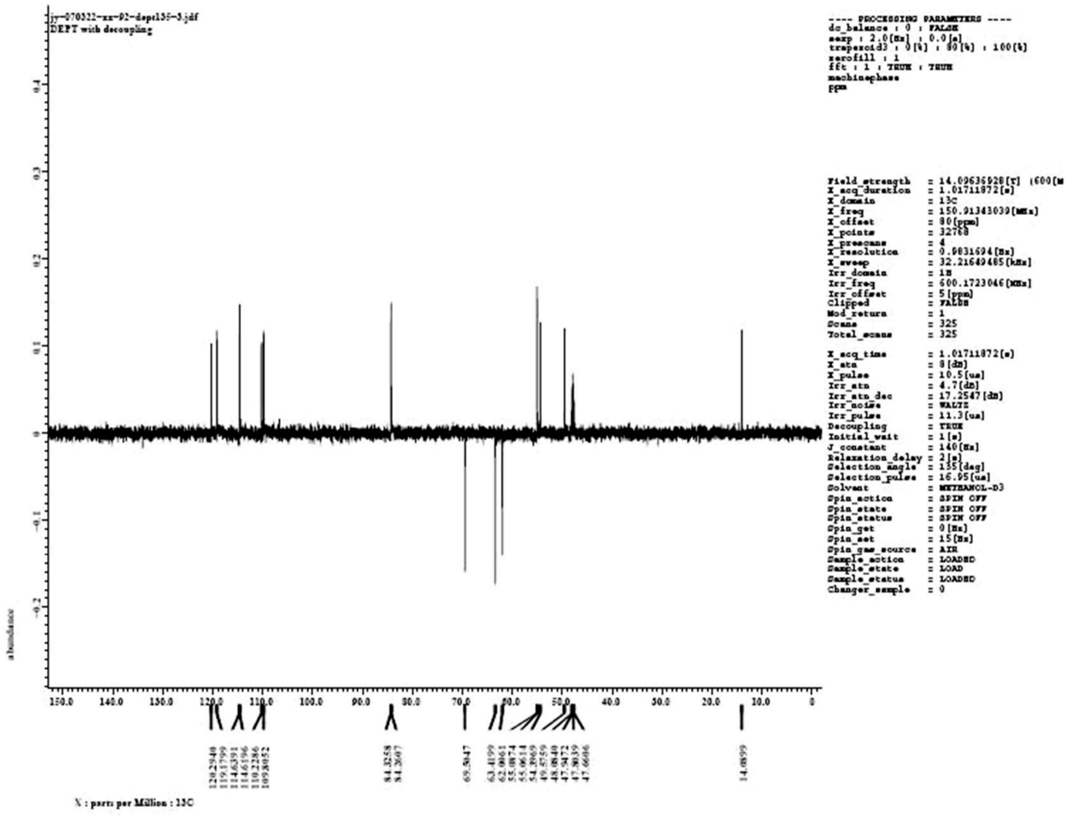

Figure S41. DEPT NMR spectrum of compound **6** in CD_3OD.

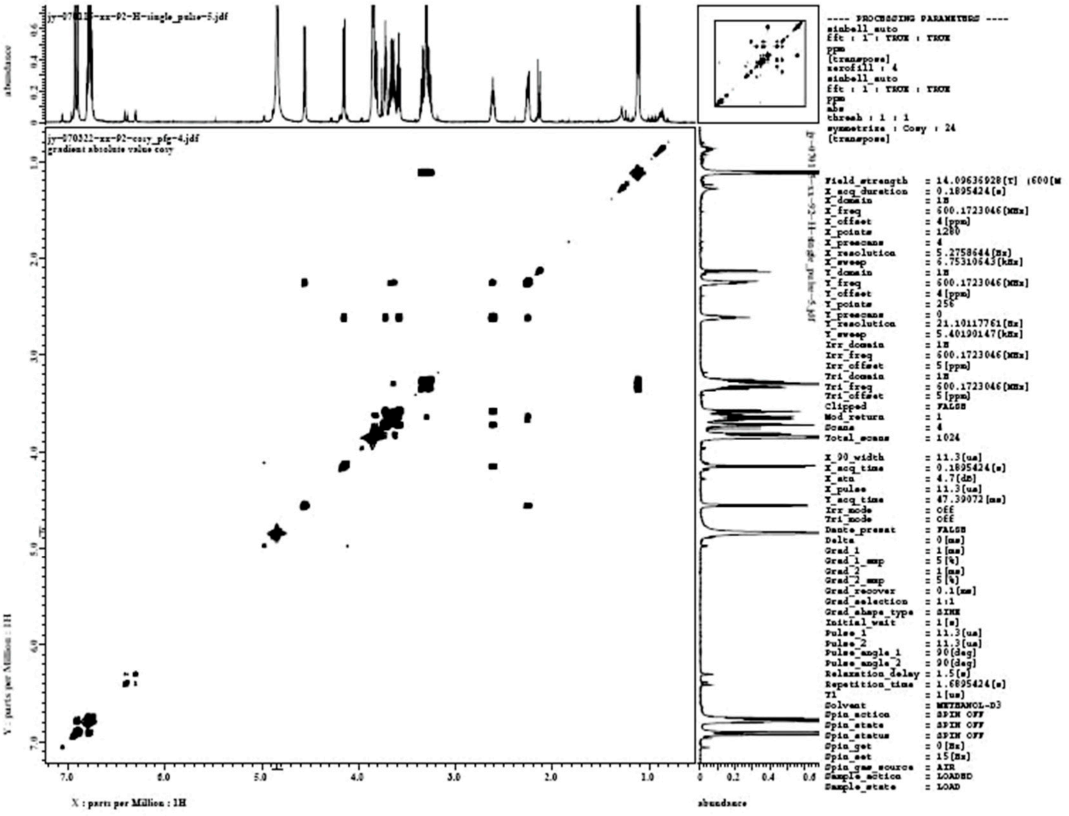

Figure S42. ^{1}H–^{1}H COSY NMR spectrum of compound **6** in CD_3OD.

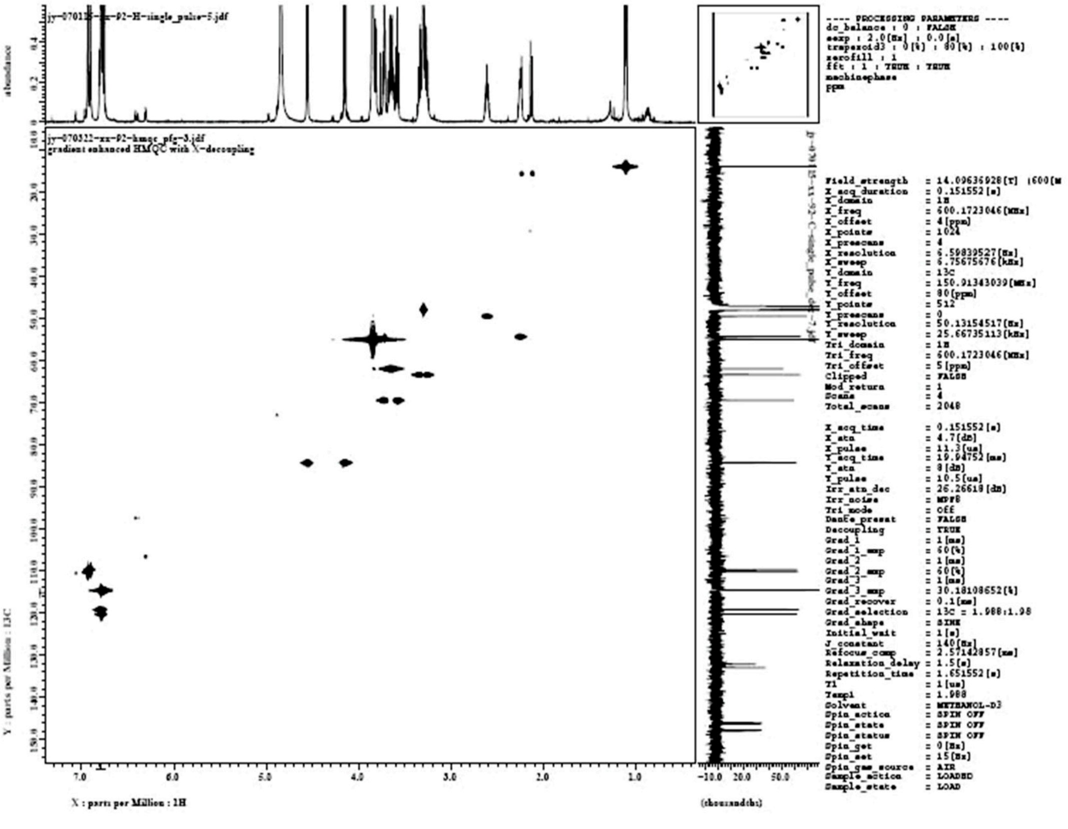

Figure S43. HMQC-NMR spectrum of compound **6** in CD_3OD.

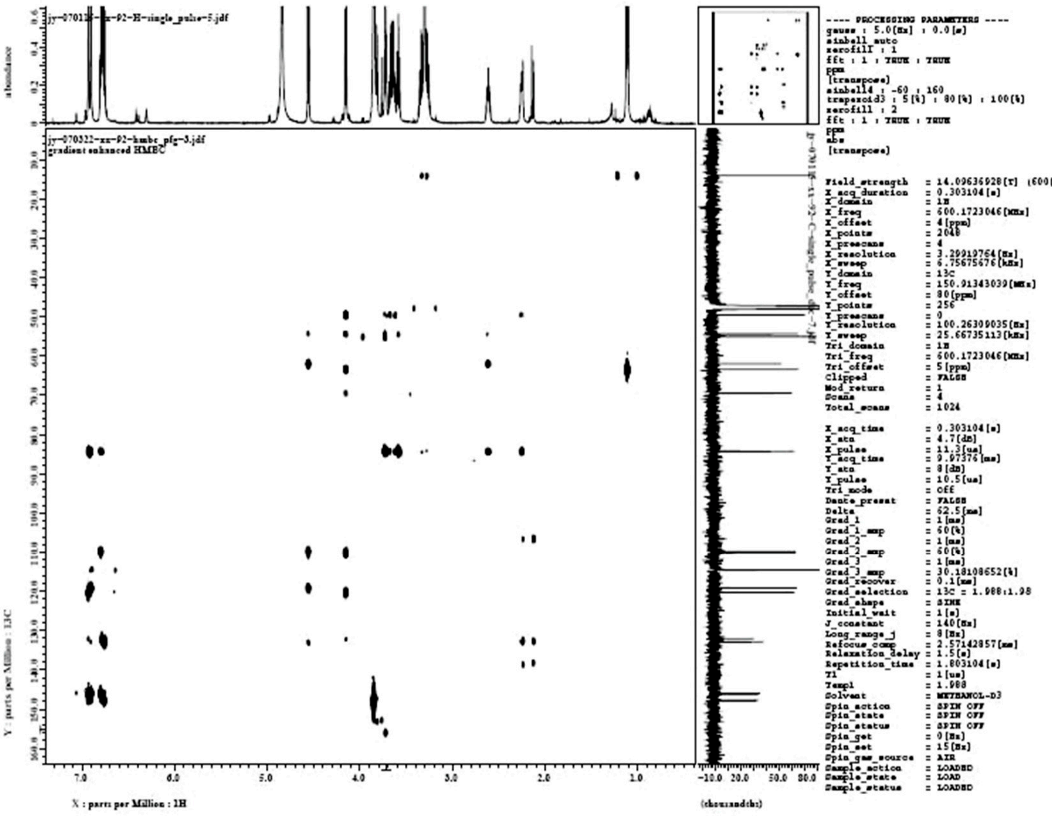

Figure S44. HMBC-NMR spectrum of compound **6** in CD_3OD.

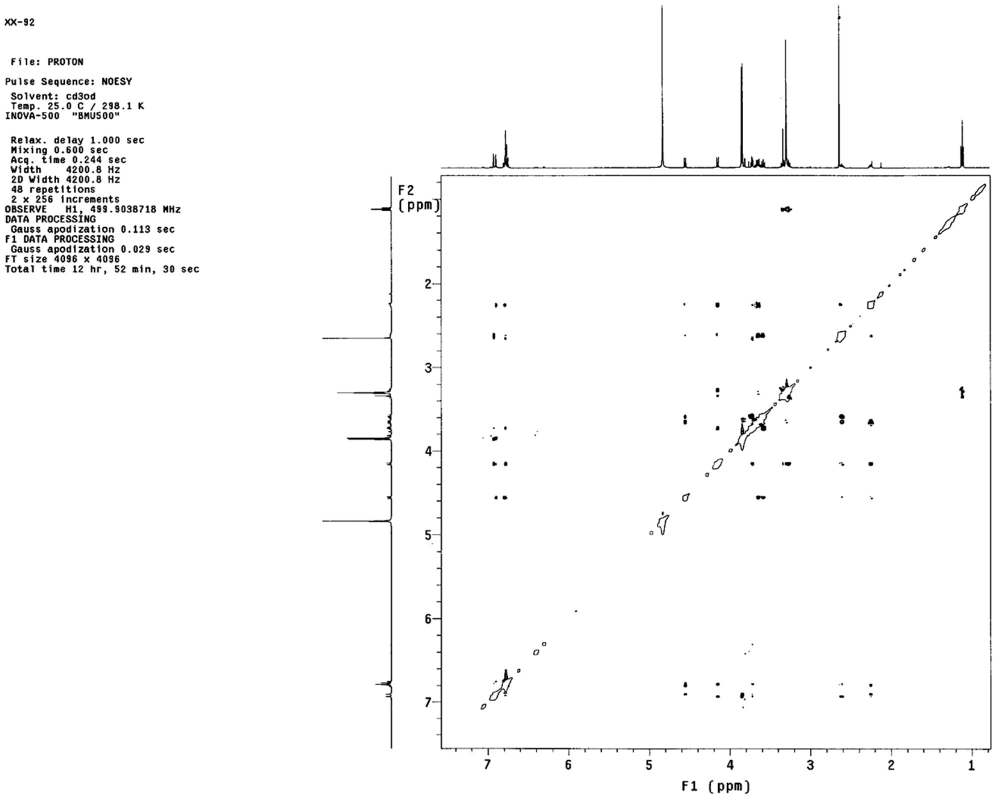

Figure S45. NOESY NMR spectrum of compound **6** in CD_3OD.

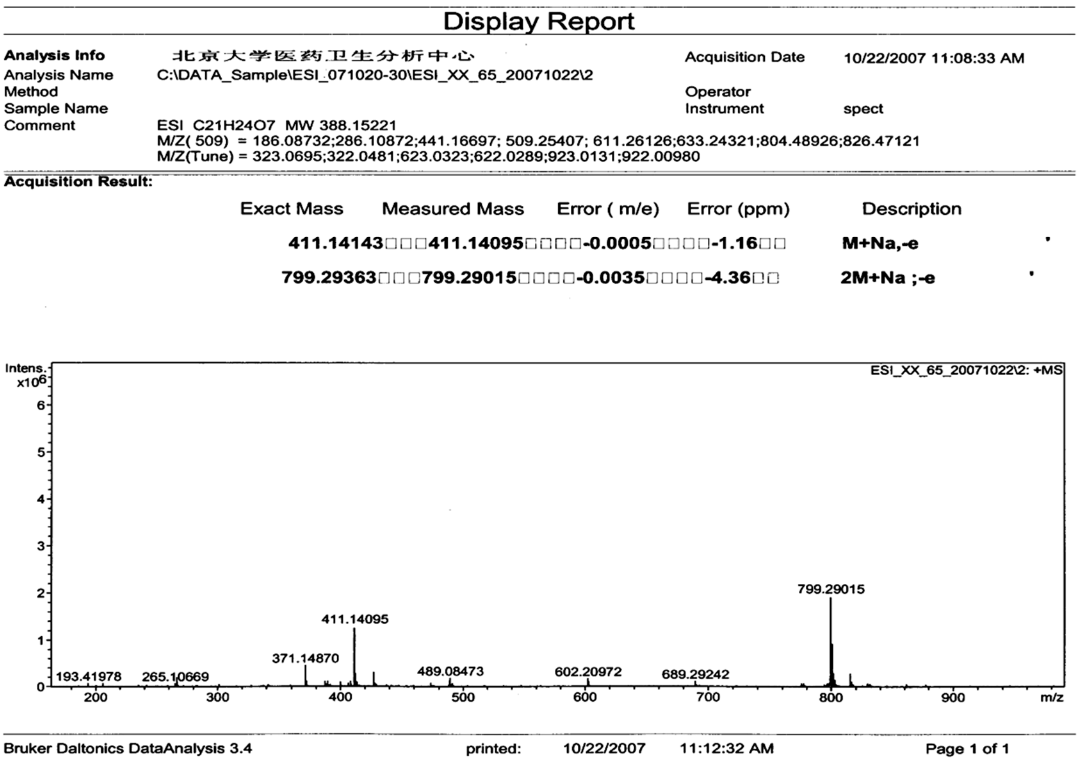

Figure S46. HRESIMS spectrum of compound **7**.

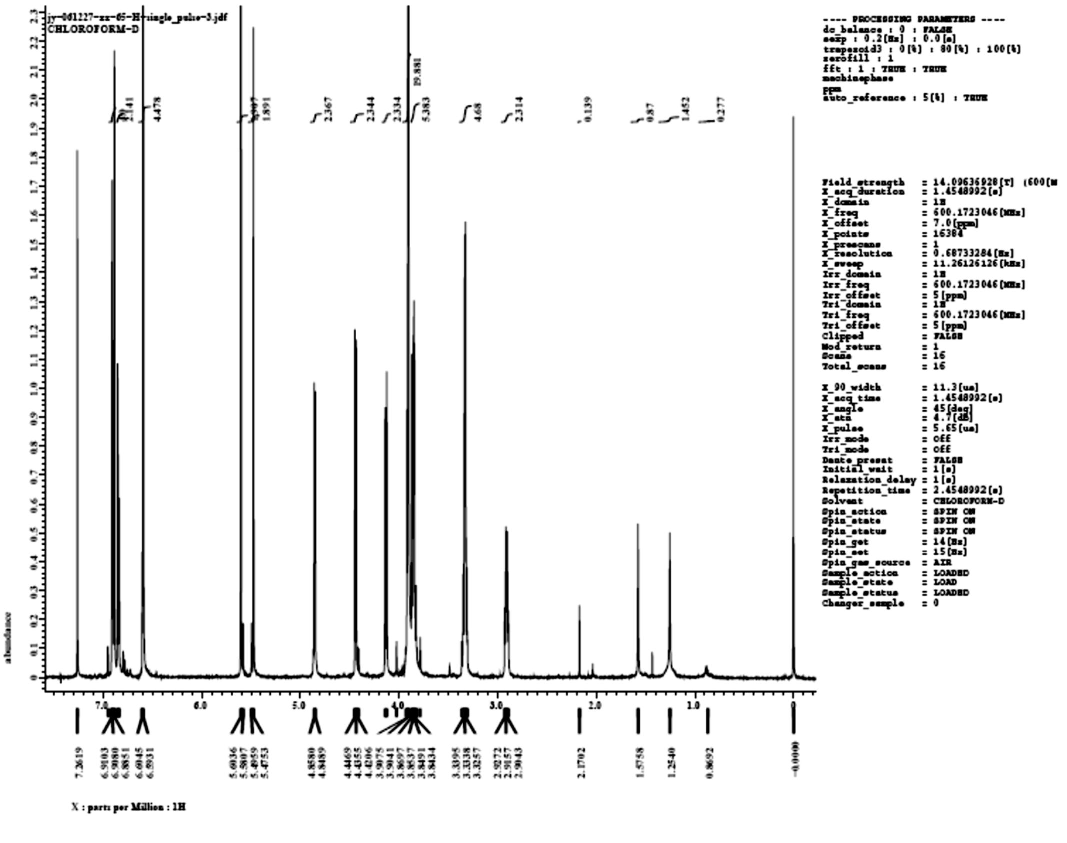

Figure S47. ^{1}H-NMR spectrum of compound **7** in $CDCl_3$.

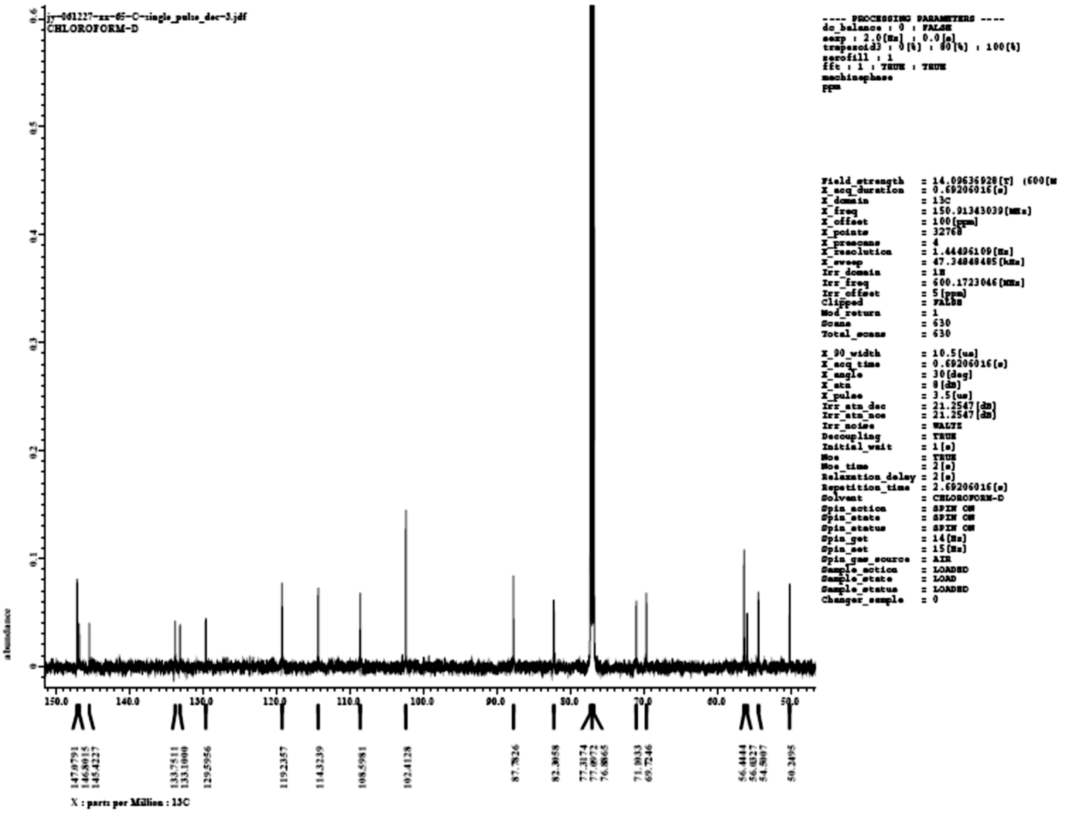

Figure S48. ^{13}C-NMR spectrum of compound **7** in $CDCl_3$.

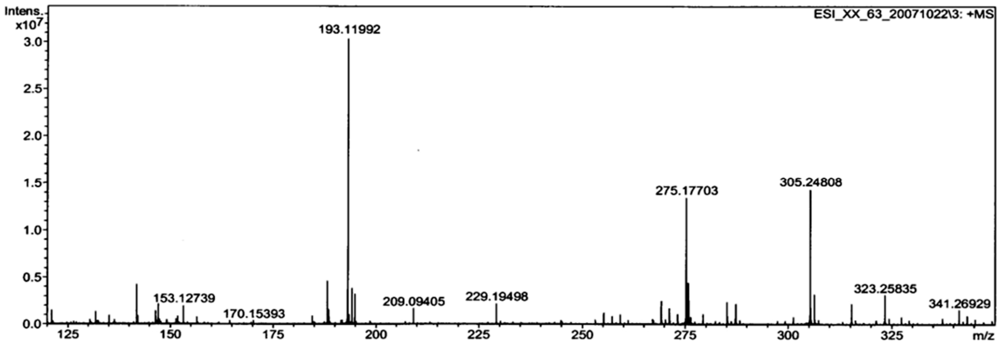

Display Report

**Analysis Info**	北京大学医药卫生分析中心	Acquisition Date	10/22/2007 2:52:25 PM
Analysis Name	C:\DATA_Sample\ESI_071020-30\ESI_XX_63_20071022\3		
Method		Operator	
Sample Name		Instrument	spect
Comment	ESI C10H16O1 MW 152.12012		

M/Z(509) = 186.08732;286.10872;441.16697; 509.25407; 611.26126;633.24321;804.48926;826.47121
M/Z(Tune) = 323.0695;322.0481;623.0323;622.0289;923.0131;922.00980

Acquisition Result:

Exact Mass	Measured Mass	Error (m/e)	Error (ppm)	Description
153.12739	153.12739	0.0000	-0.01	M+H , -e
305.24751	305.24808	0.0006	1.88	2M+H ;-e

Bruker Daltonics DataAnalysis 3.4 printed: 10/22/2007 2:55:58 PM Page 1 of 1

Figure S49. HRESIMS spectrum of compound **8**.

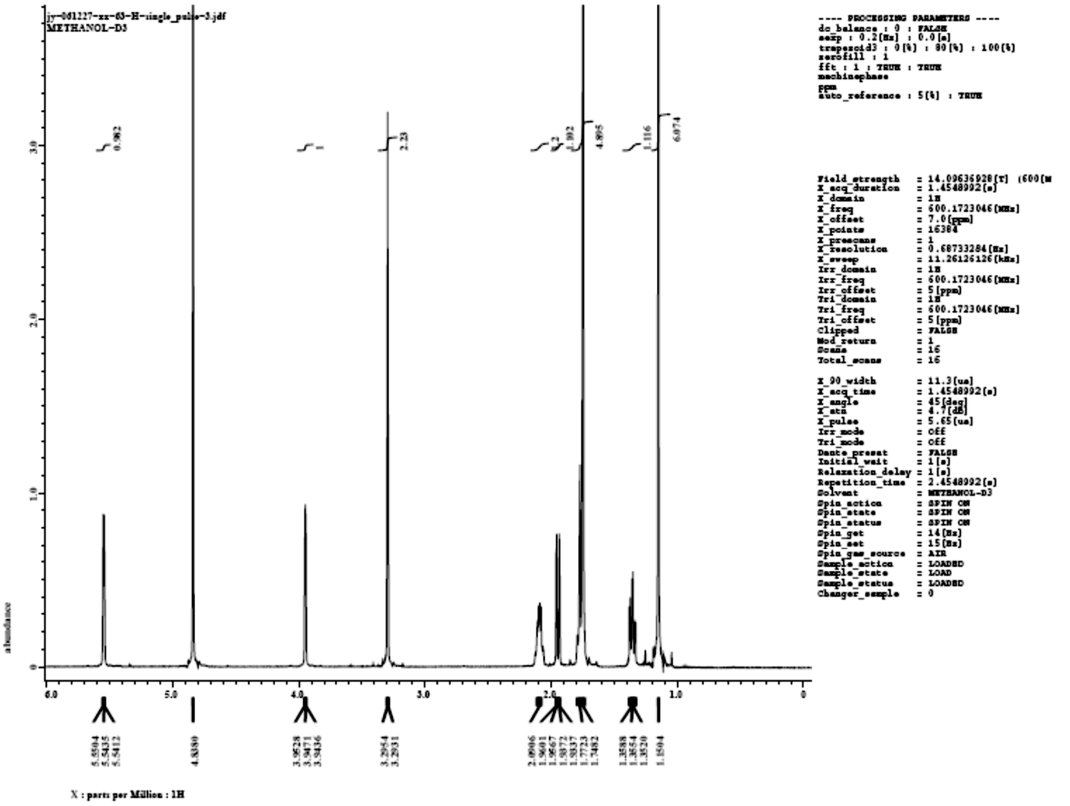

Figure S50. ^{1}H-NMR spectrum of compound **8** in CD_3OD.

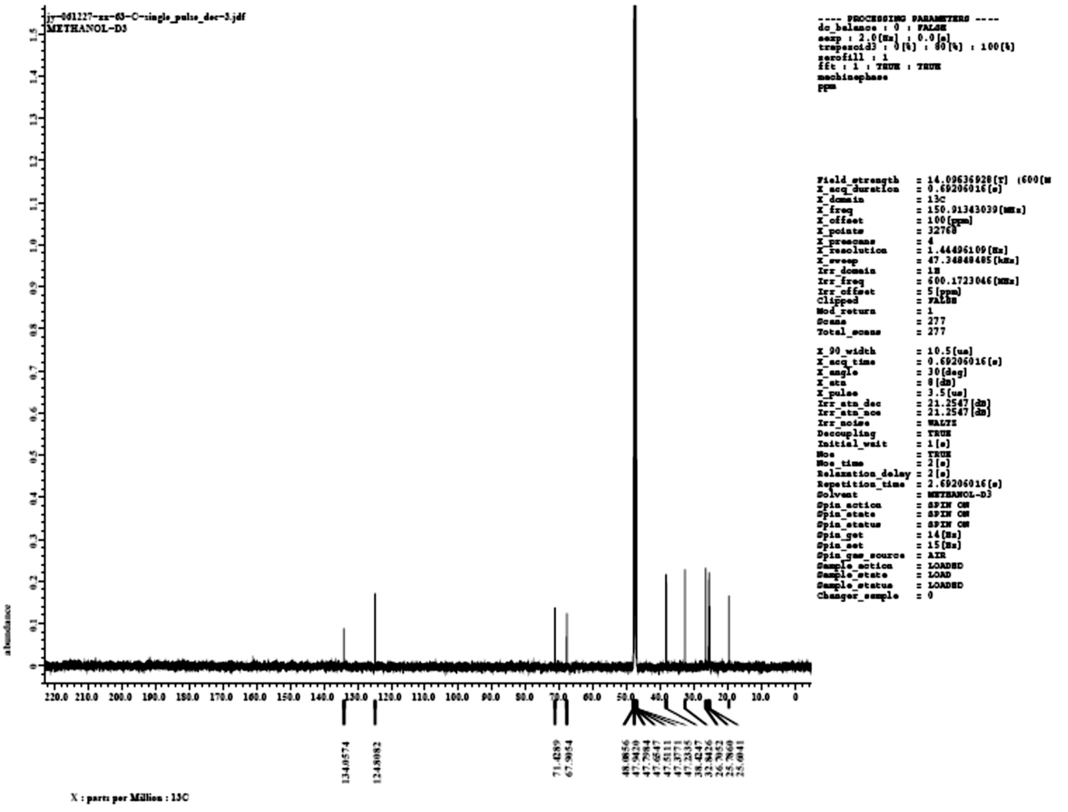

Figure S51. ^{13}C-NMR spectrum of compound **8** in CD_3OD.

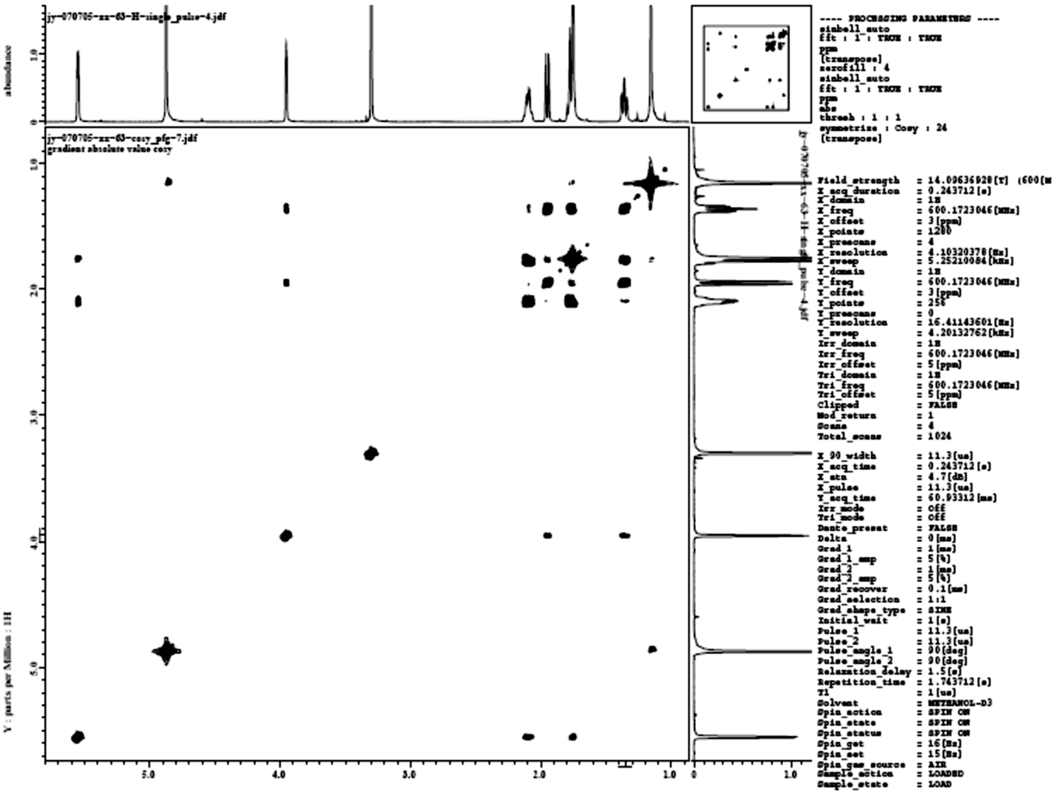

Figure S52.^{1}H–^{1}H COSY NMR spectrum of compound **8** in CD_3OD.

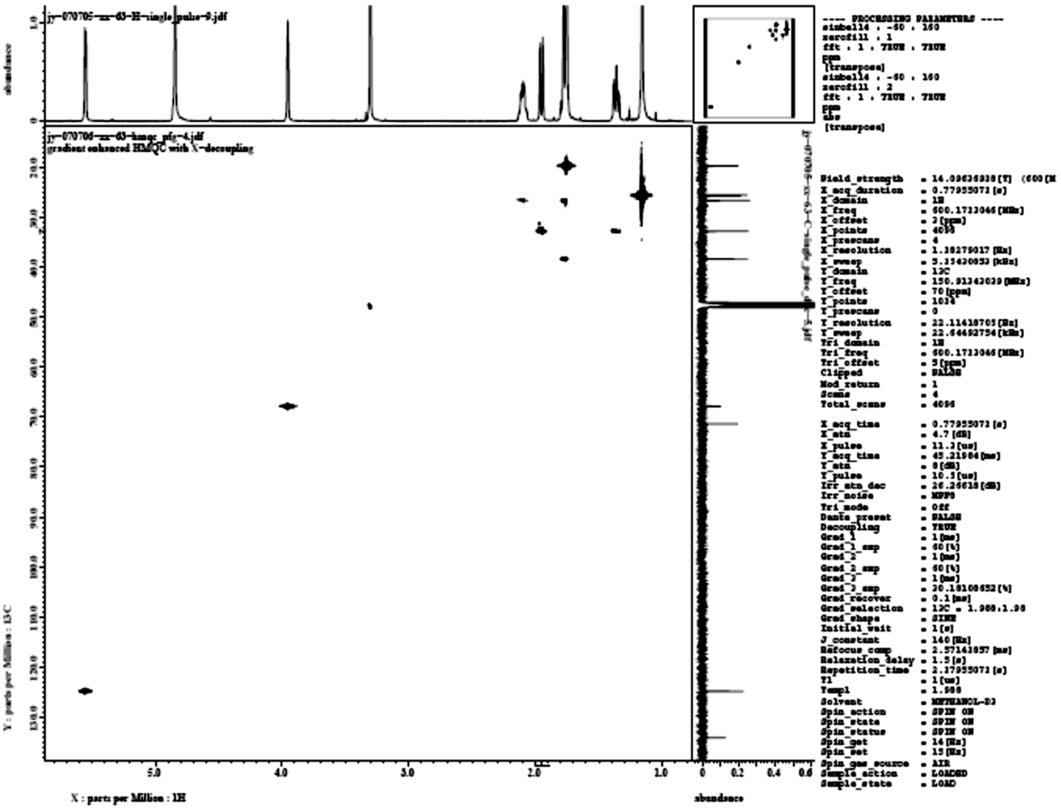

Figure S53. HMQC-NMR spectrum of compound **8** in CD_3OD.

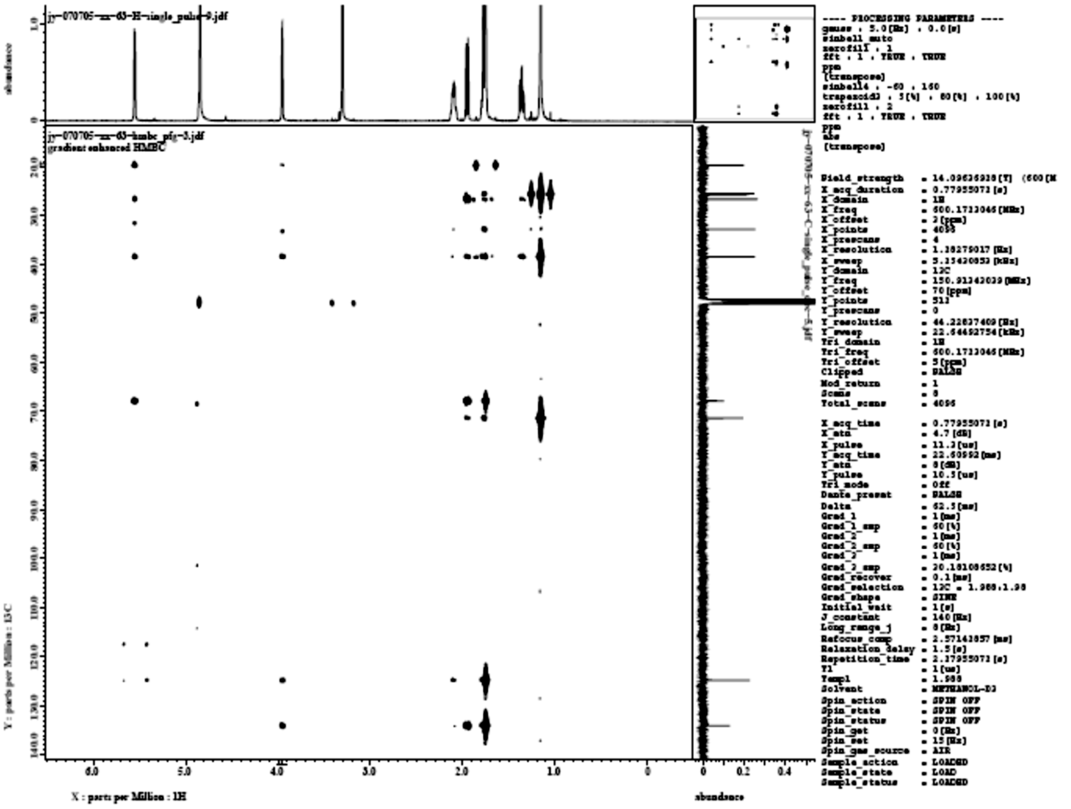

Figure S54. HMBC-NMR spectrum of compound **8** in CD_3OD.

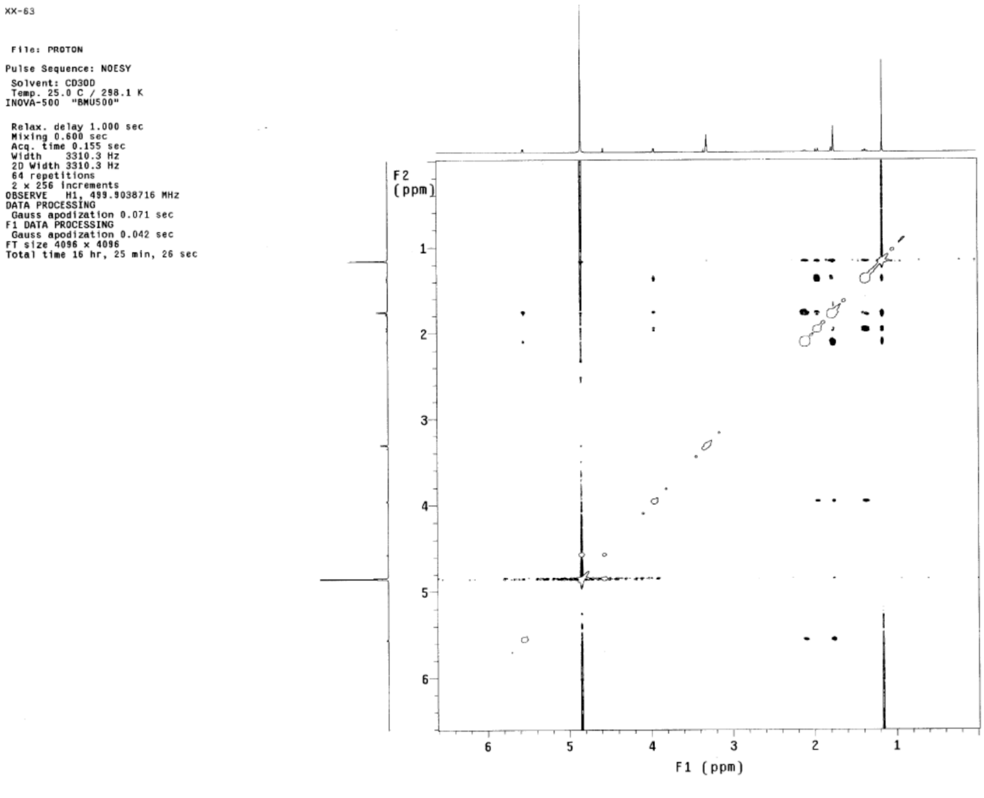

Figure S55. NOESY NMR spectrum of compound **8** in CD_3OD.

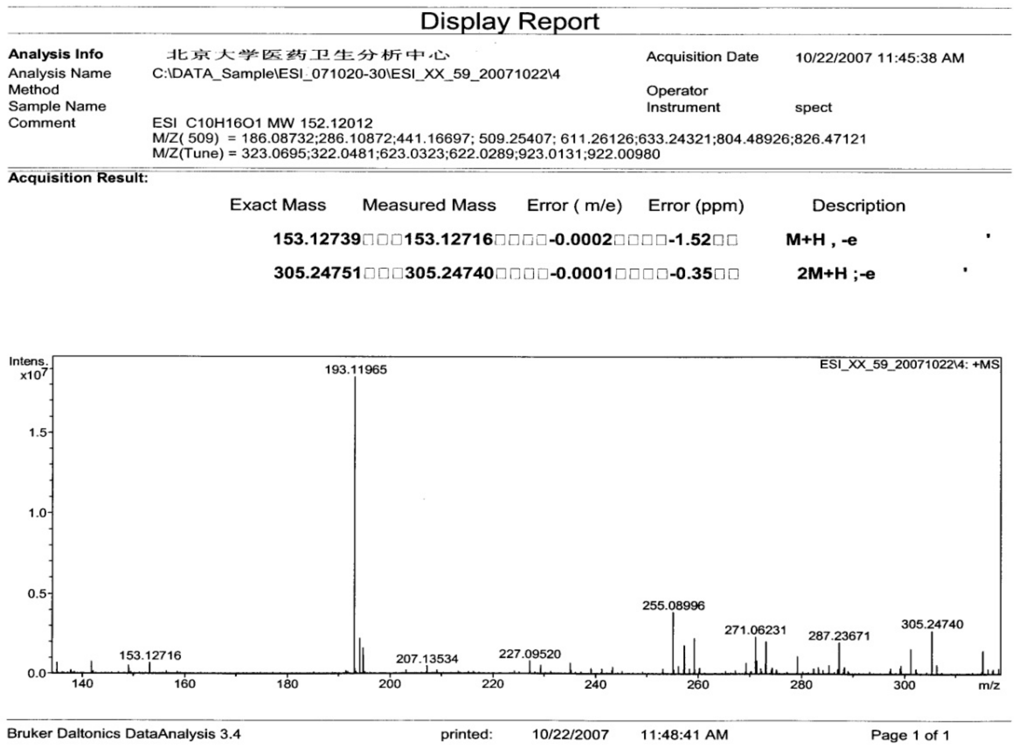

Figure S56. HRESIMS spectrum of compound **9**.

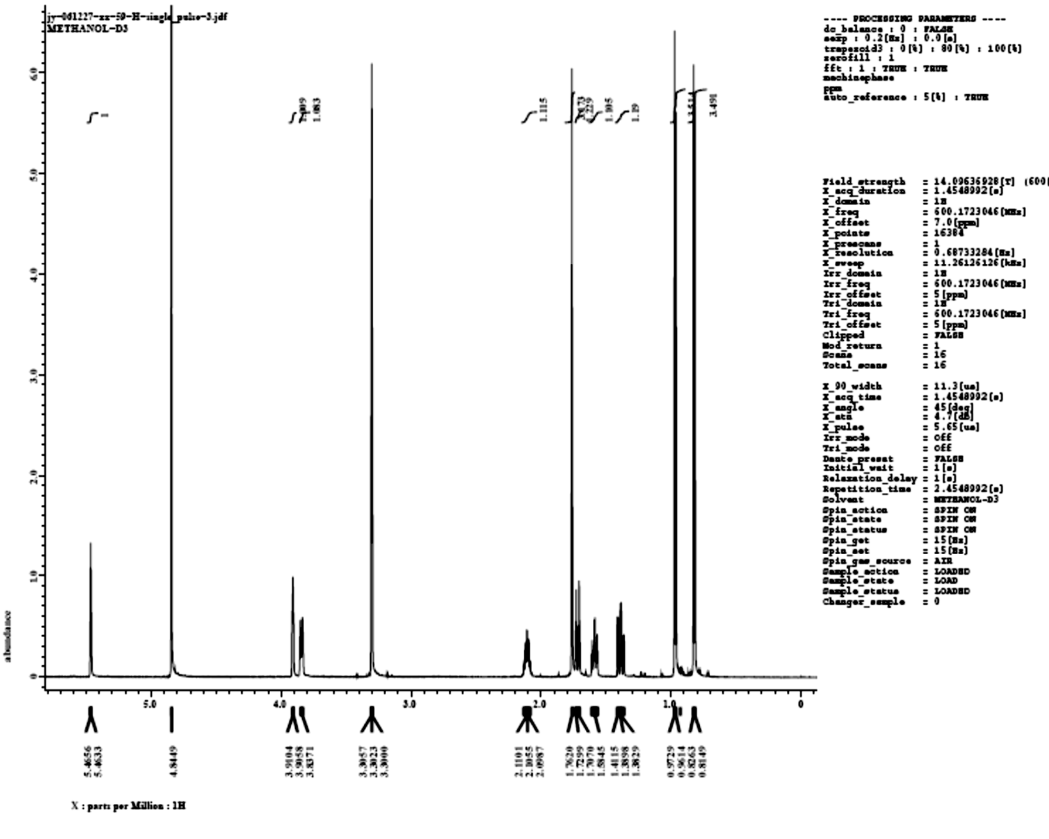

Figure S57. ^{1}H-NMR spectrum of compound **9** in CD_3OD.

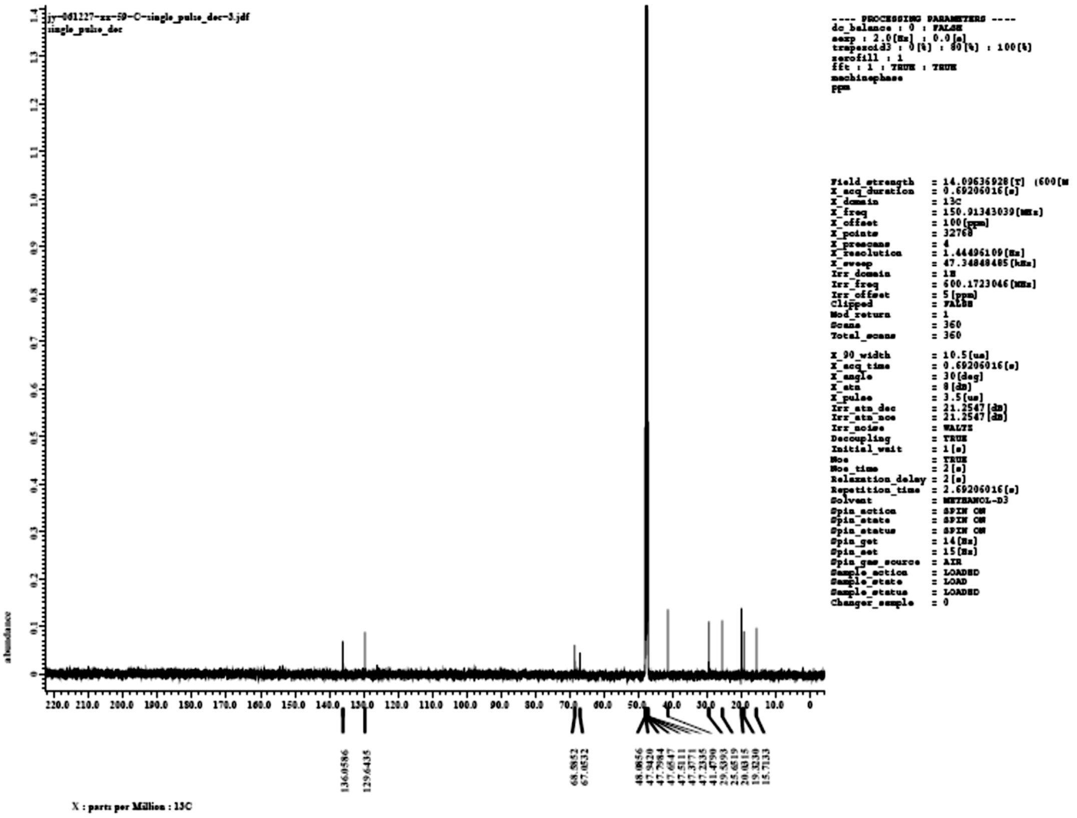

Figure S58. ^{13}C-NMR spectrum of compound **9** in CD_3OD.

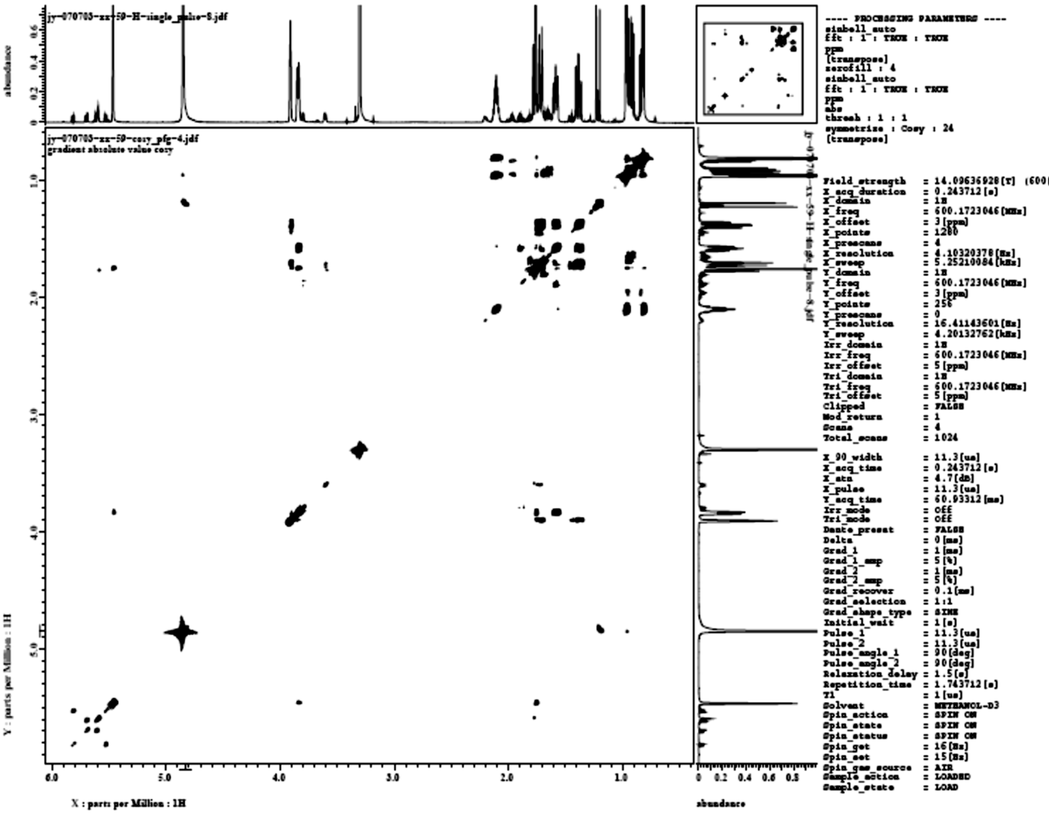

Figure S59. ^{1}H–^{1}H COSY NMR spectrum of compound **9** in CD_3OD.

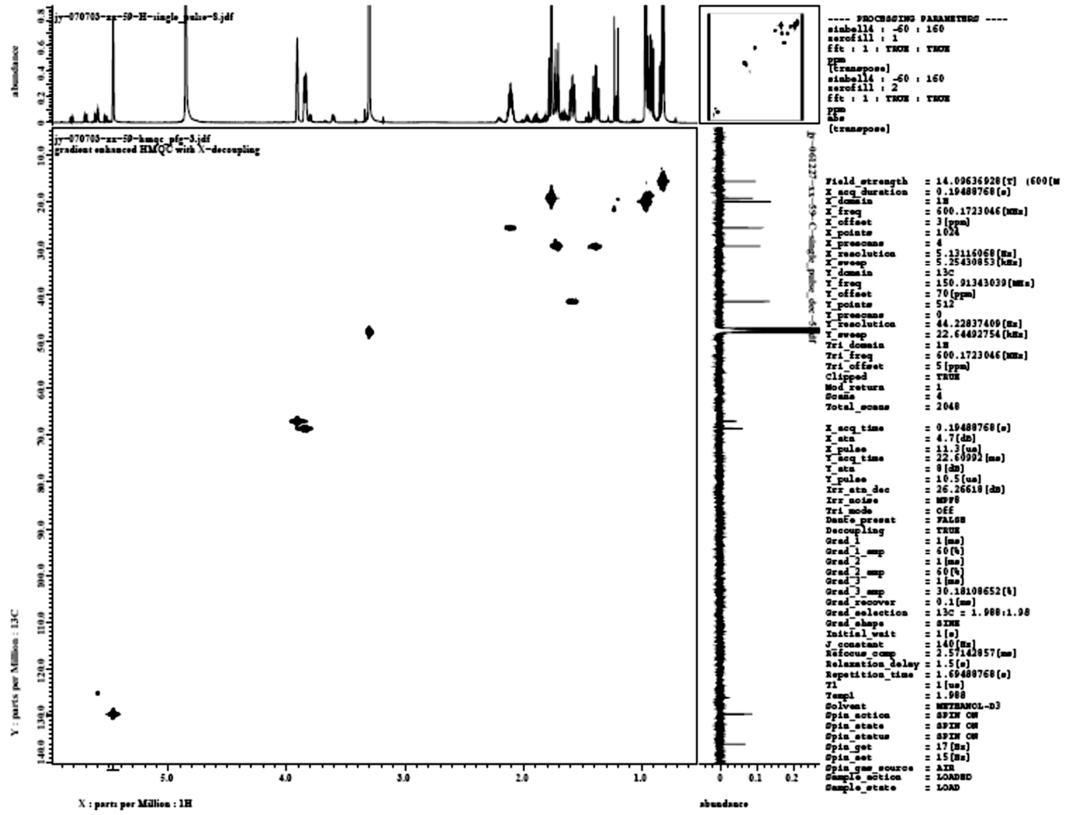

Figure S60. HMQC-NMR spectrum of compound **9** in CD_3OD.

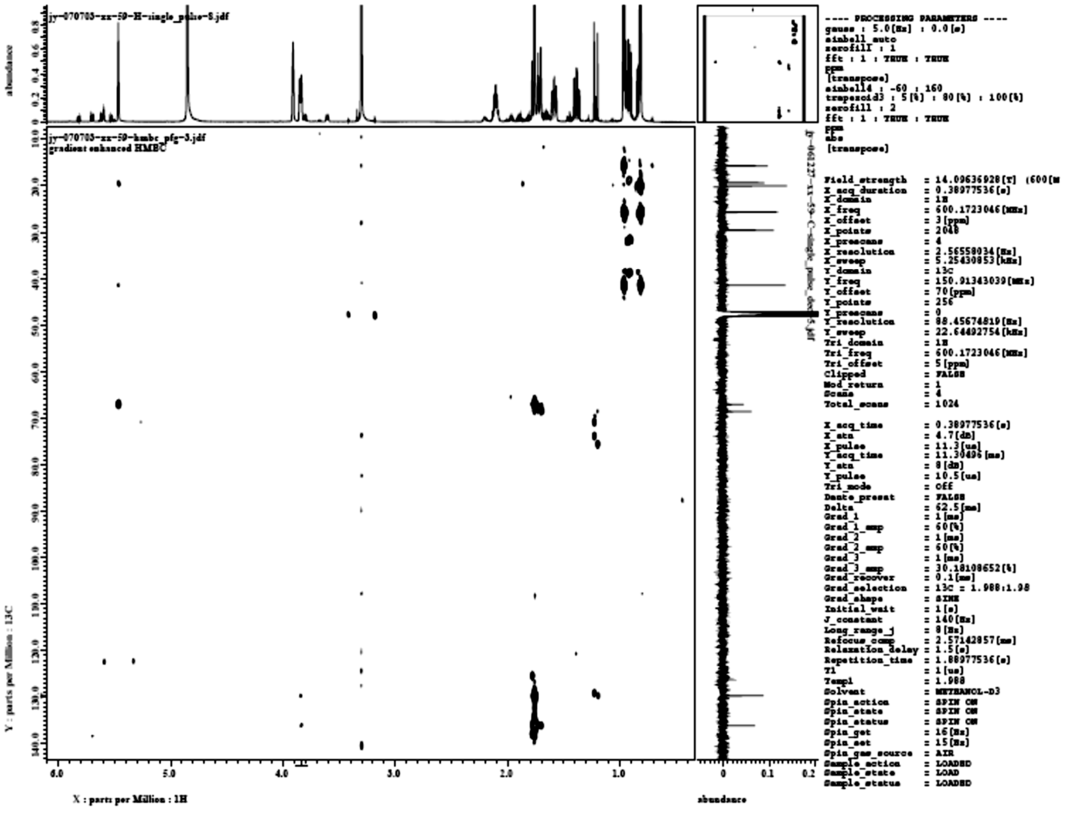

Figure S61. HMBC-NMR spectrum of compound **9** in CD_3OD.

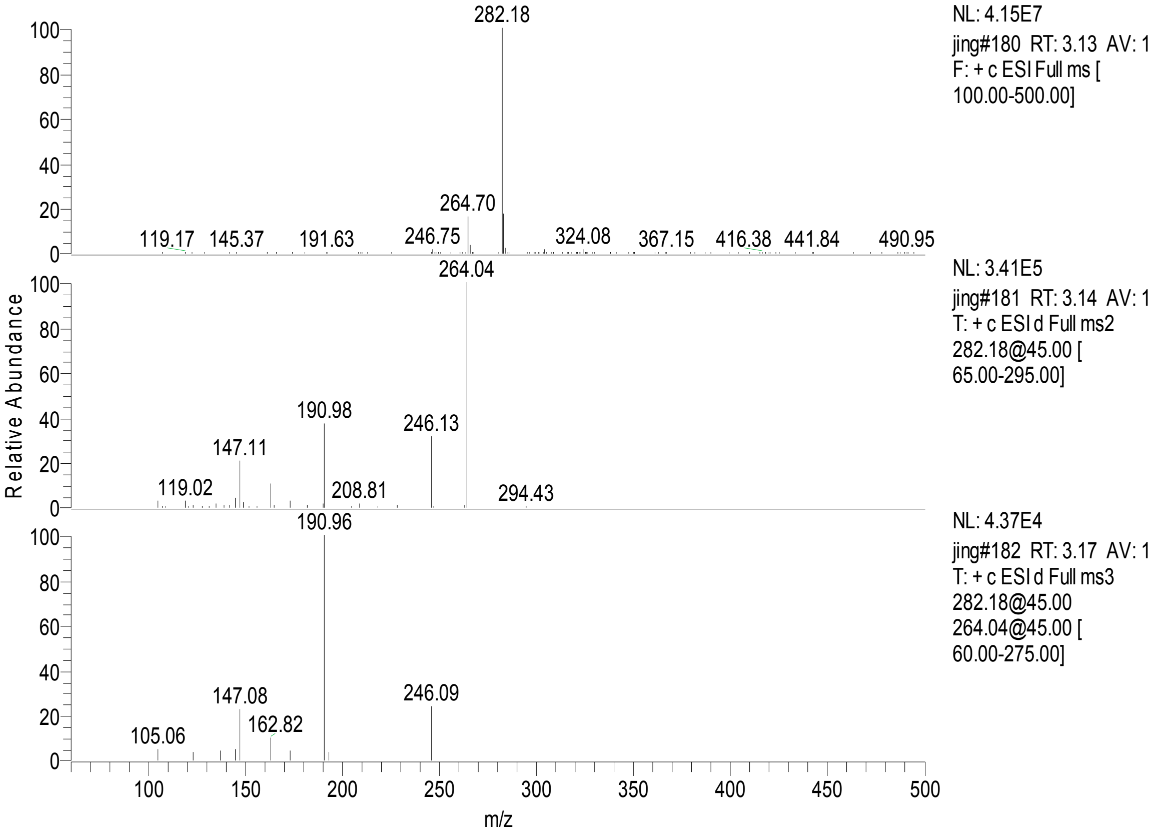

Figure S62. HPLC-MS/MS spectrum of compound **10**.

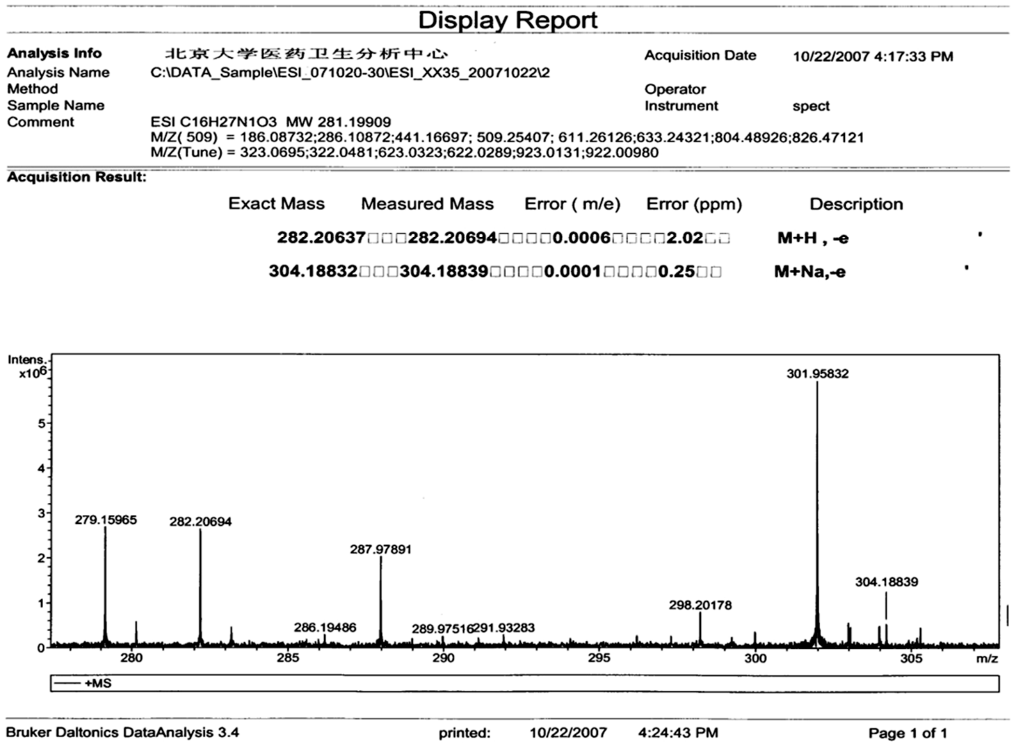

Figure S63. HRESIMS spectrum of compound **10**.

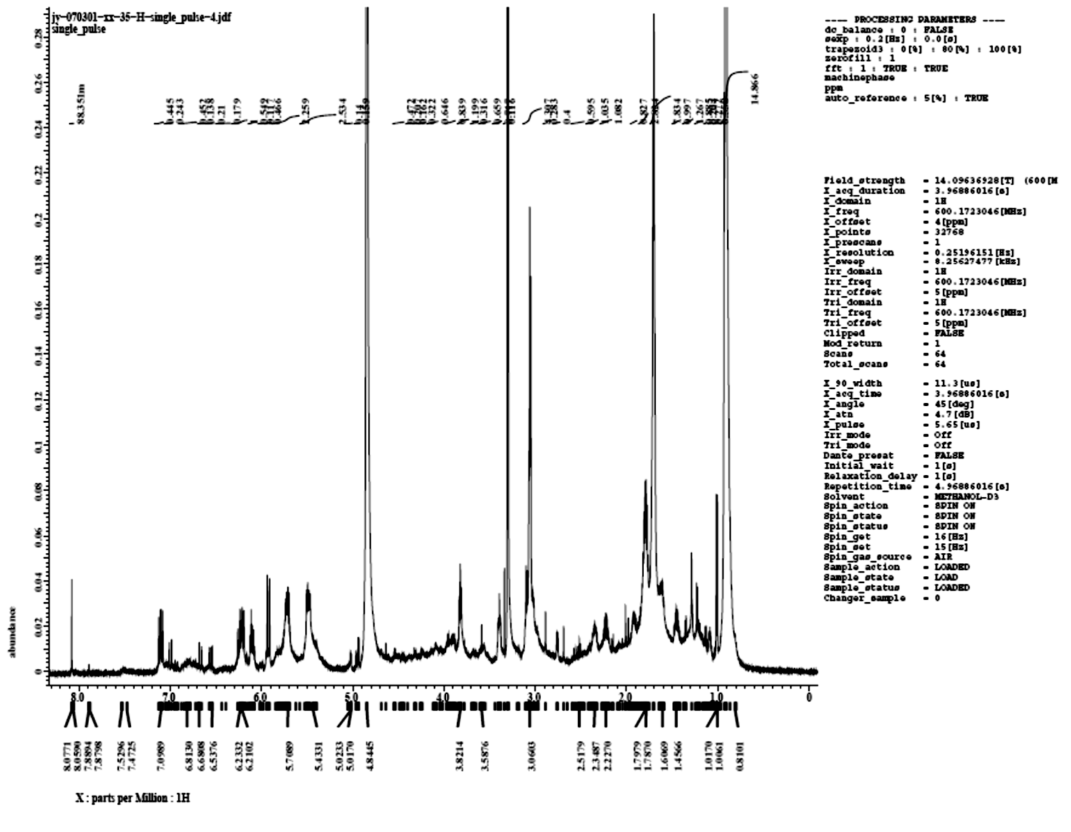

Figure S64. ^{1}H-NMR spectrum of compound **10** in CD_3OD (25 °C).

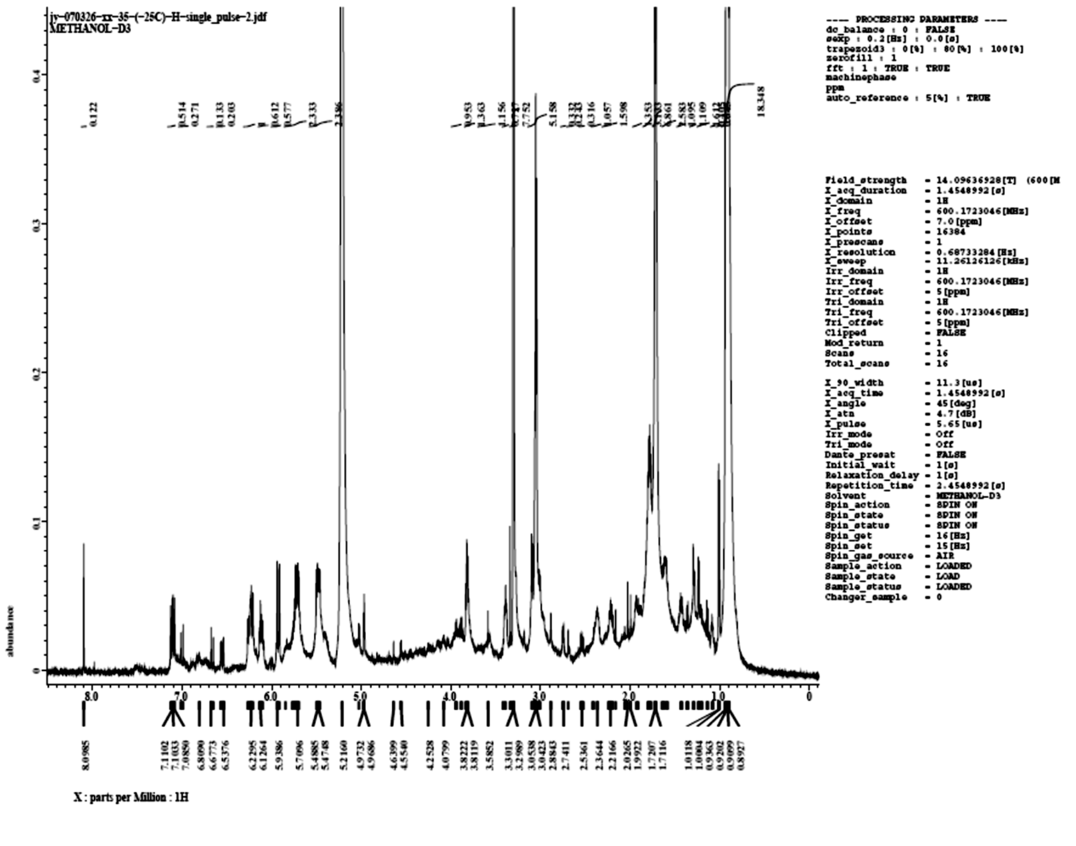

Figure S65. ^{1}H-NMR spectrum of compound **10** in CD_3OD (–25 °C).

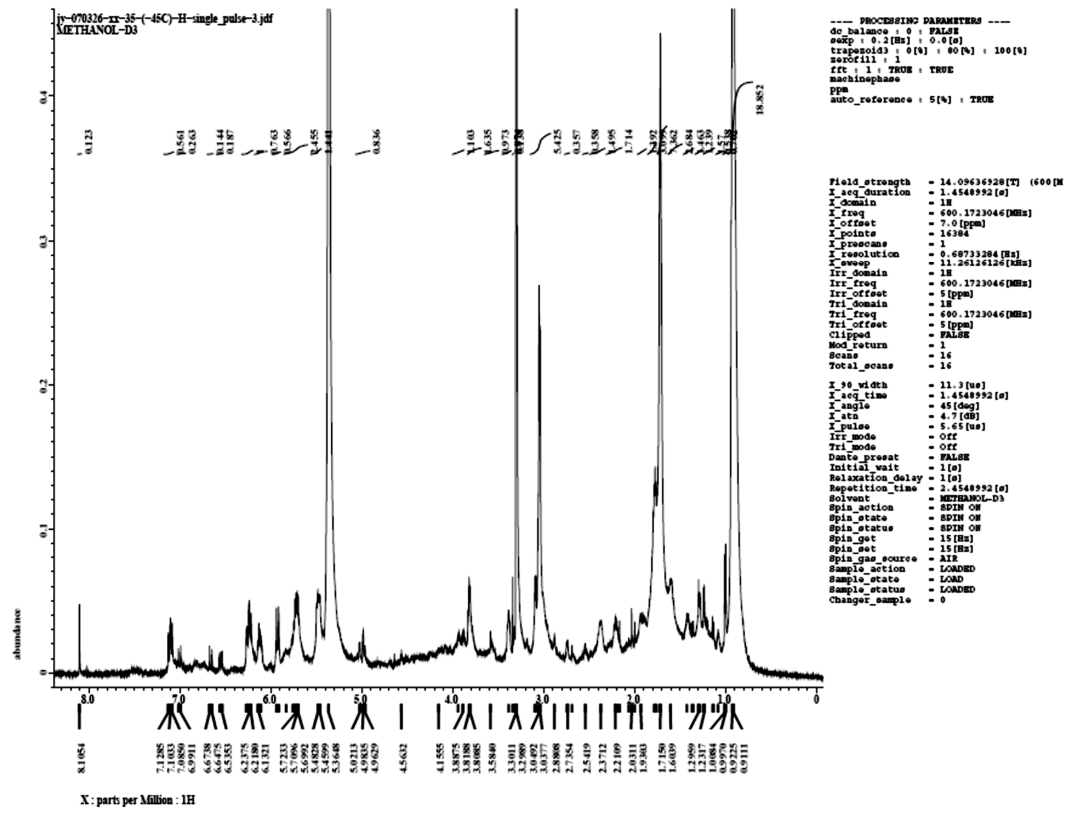

Figure S66. ^{1}H-NMR spectrum of compound **10** in CD_3OD (–45 °C).

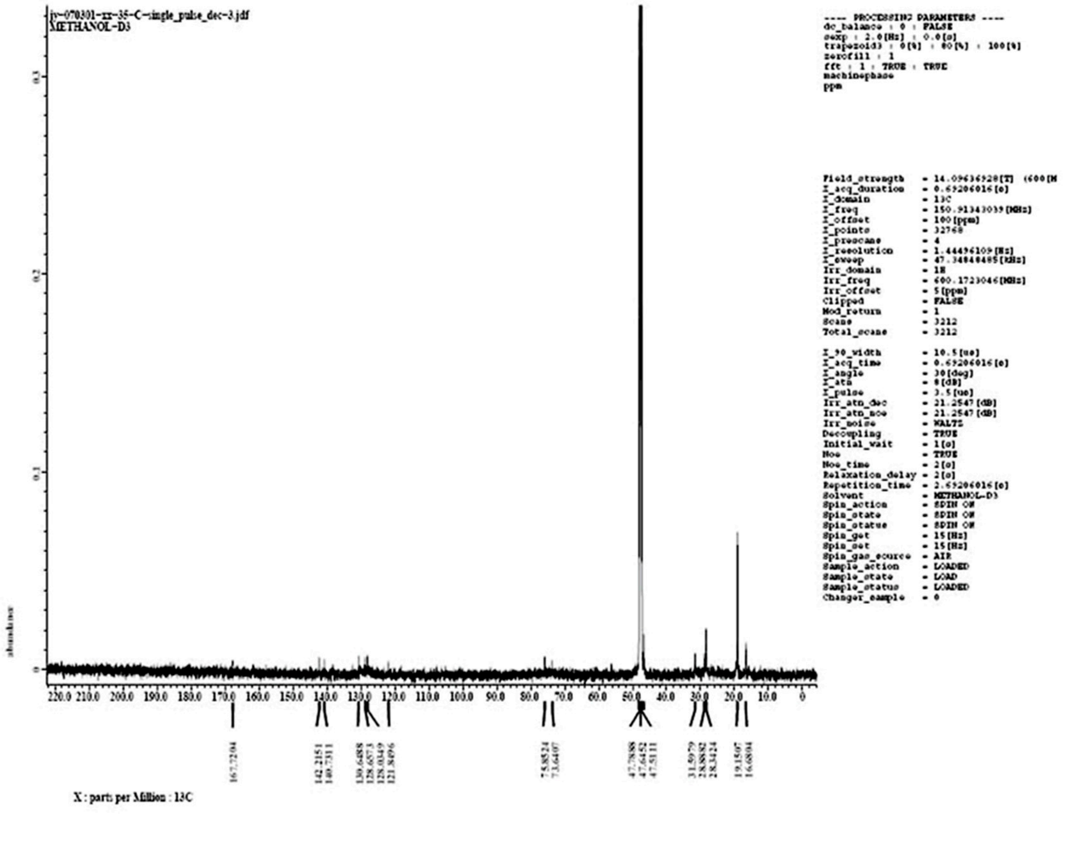

Figure S67. ^{13}C-NMR spectrum of compound **10** in CD_3OD.

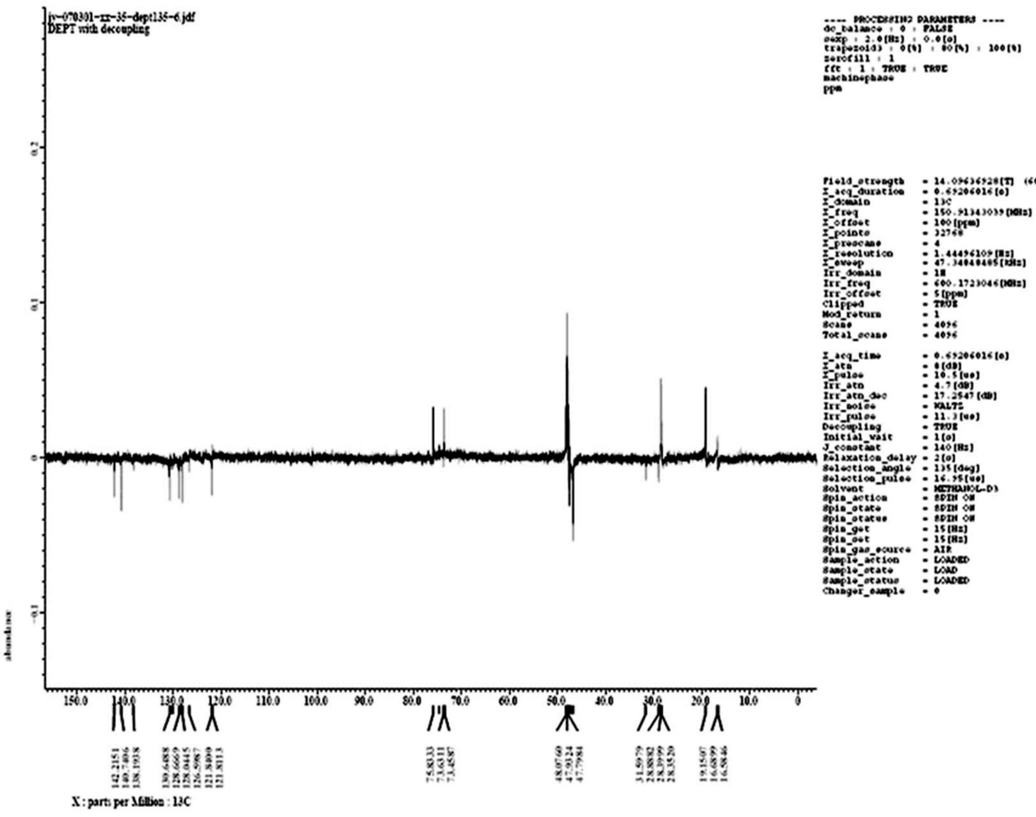

Figure S68. DEPT NMR spectrum of compound **10** in CD_3OD.

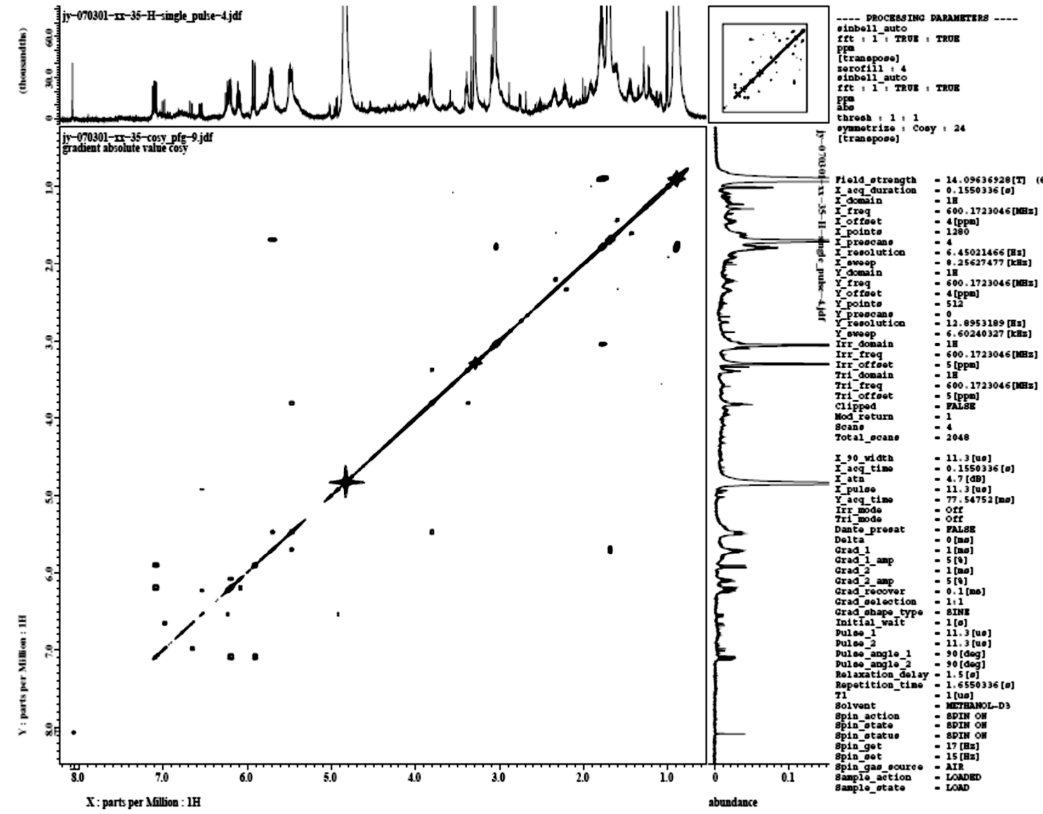

Figure S69. ^{1}H–^{1}H COSY NMR spectrum of compound **10** in CD_3OD.

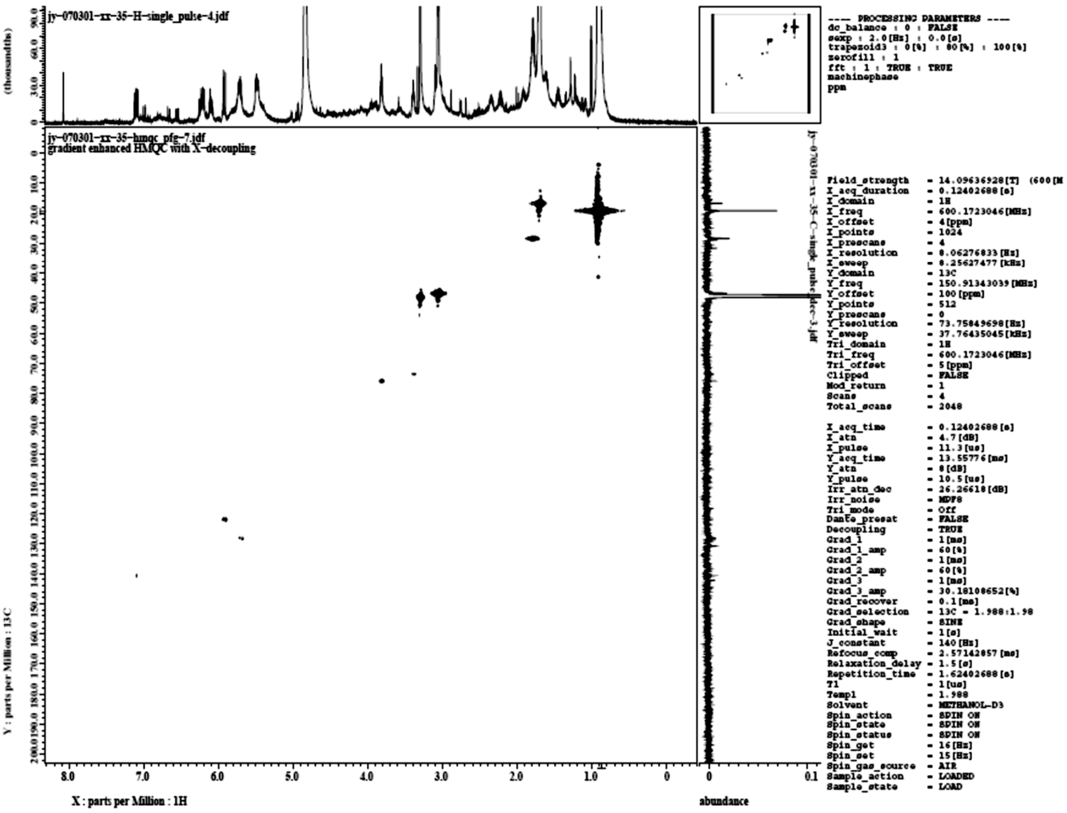

Figure S70. HMQC-NMR spectrum of compound **10** in CD_3OD.

Display Report

**Analysis Info**	北京大学医药卫生分析中心	Acquisition Date	10/22/2007 4:08:35 PM
Analysis Name	C:\DATA_Sample\ESI_071020-30\ESI_XX32_20071022\5		
Method		Operator	
Sample Name		Instrument	spect
Comment	ESI C16H27N1O3 MW 281.19909<br>M/Z(509) = 186.08732;286.10872;441.16697; 509.25407; 611.26126;633.24321;804.48926;826.47121<br>M/Z(Tune) = 323.0695;322.0481;623.0323;622.0289;923.0131;922.00980		

Acquisition Result:

Exact Mass	Measured Mass	Error (m/e)	Error (ppm)	Description
**282.20637**	**282.20632**	**-0.0001**	**-0.18**	**M+H , -e**
**304.18832**	**304.18857**	**0.0003**	**0.84**	**M+Na,-e**

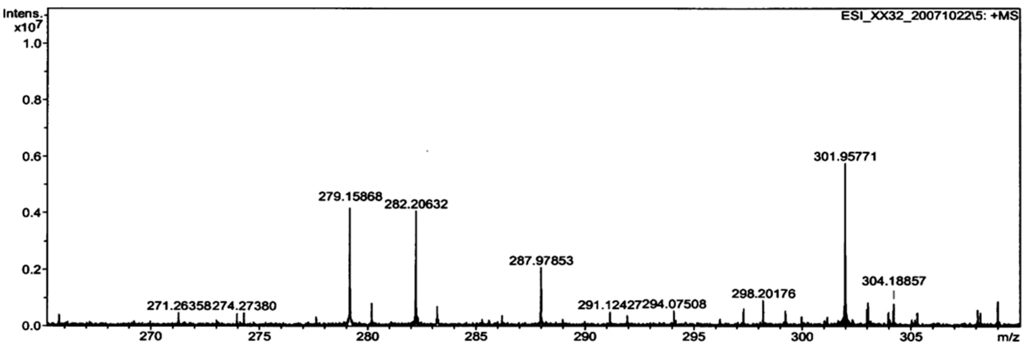

Bruker Daltonics DataAnalysis 3.4 printed: 10/22/2007 4:11:20 PM Page 1 of 1

Figure S71. HRESIMS spectrum of compound **11**.

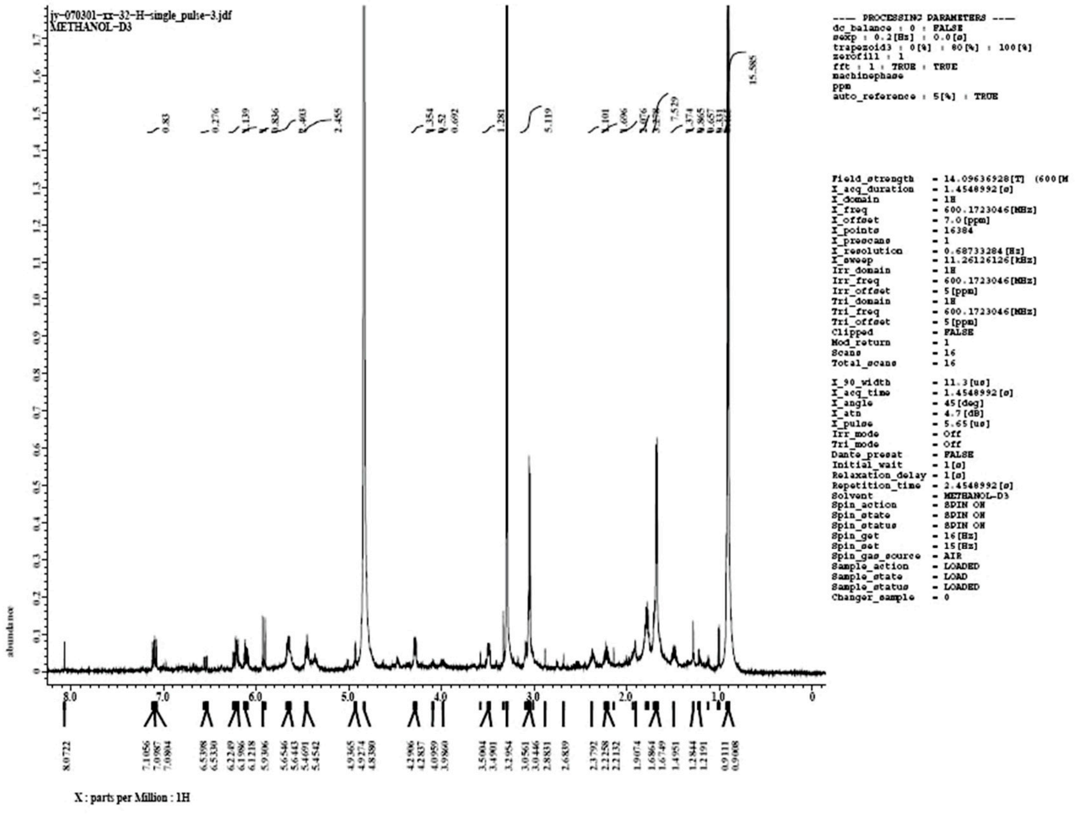

Figure S72. ^{1}H-NMR spectrum of compound **11** in CD_3OD.

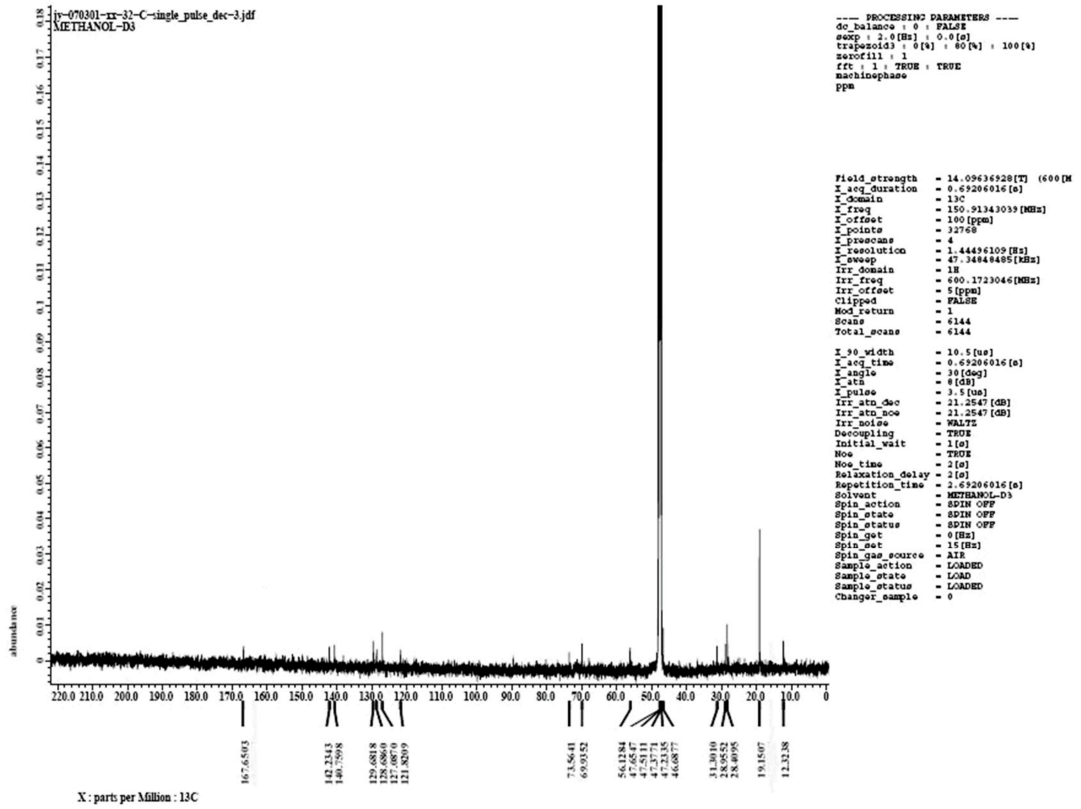

Figure S73. ^{13}C-NMR spectrum of compound **11** in CD_3OD.

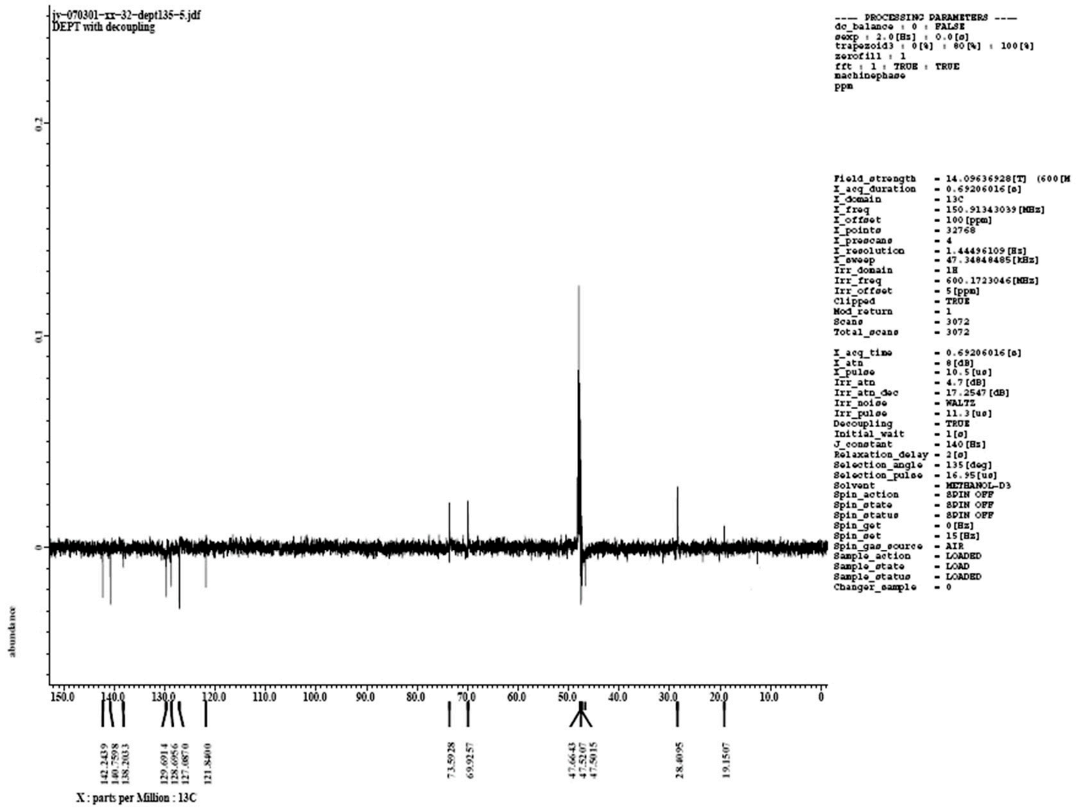

Figure S74. DEPT NMR spectrum of compound **11** in CD_3OD.

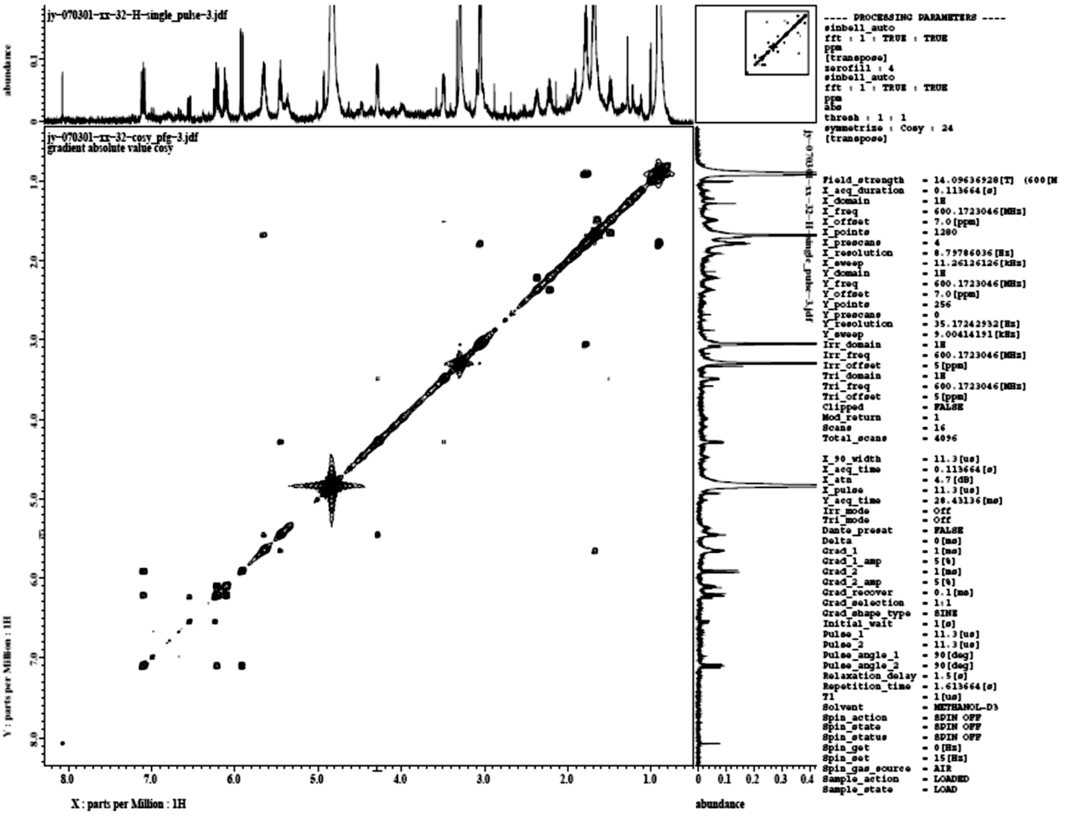

Figure S75. ^1H–^1H COSY NMR spectrum of compound **11** in CD_3OD.

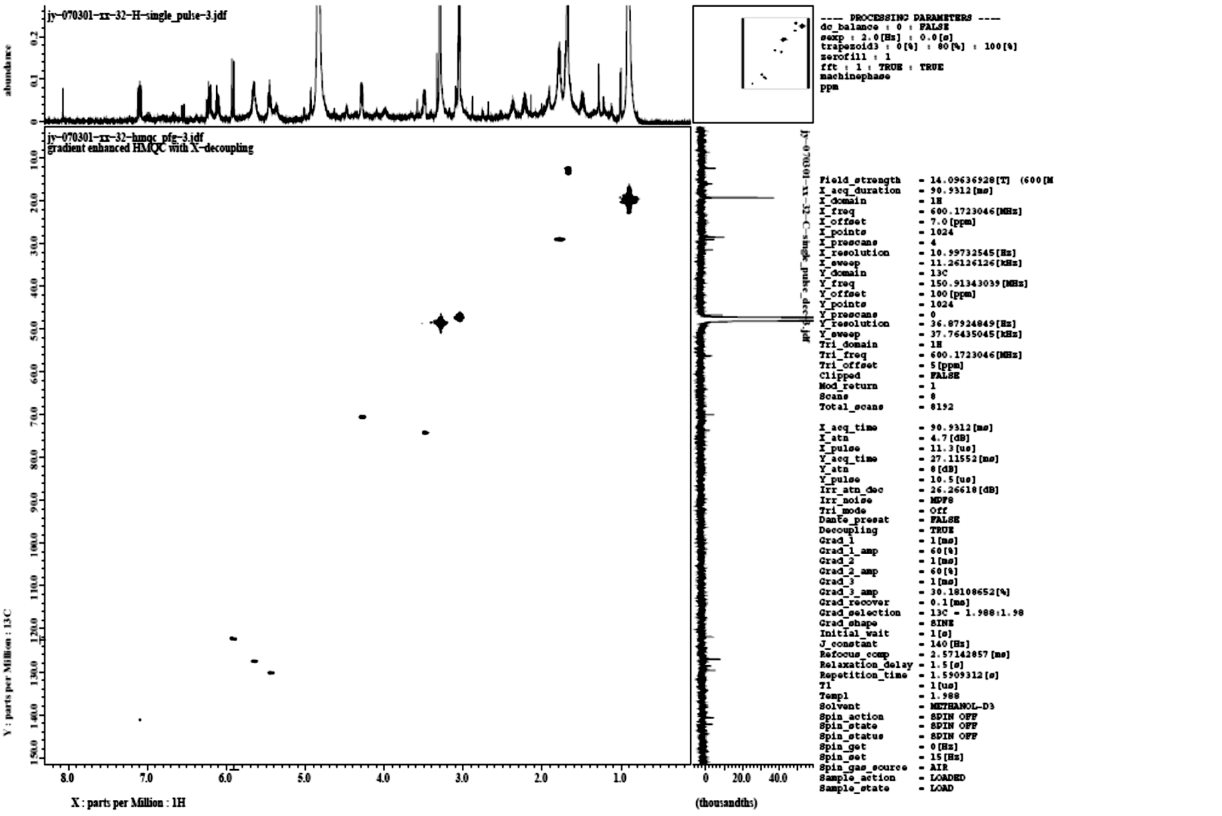

Figure S76. HMQC-NMR spectrum of compound **11** in CD_3OD.

2. Extraction and Isolation of Known Compounds 13–47

The air-dried and powdered roots and rhizomes of *A. heterotropoides* var. *mandshuricum* (36 kg) were extracted three times (2 h, 1.5 h, 1.5 h for each) under reflux with 95% ethanol and then three times (2 h, 1.5 h, 1.5 h for each) with 50% ethanol successively. The combined extracts were concentrated under reduced pressure to give a dark brown residue (9.2 kg), then 7.8 kg of it was suspended in H_2O (16.5 L) and partitioned sequentially with petroleum ether (Pet.) (60–90 °C °C) (4 × 5 L), $CHCl_3$ (4 × 5 L), EtOAc (4 × 5 L), and *n*-BuOH (4 × 5 L), respectively. The $CHCl_3$ layer (231 g) was fractionated on silica gel CC eluting with a gradient of petroleum ether–EtOAc (10:1 to 0:1) to obtain 16 fractions C1–C16. Fraction C5 and C6 were left to stand overnight and colorless columnar crystal as precipitate were collected, the colorless crystal was purified by recrystallized from EtOAc to afford compound **14** (10 g). Fraction C7 produced two different colorless needle crystals when standing overnight, and then the two needle crystals were further recrystallized from EtOAc to obtain compounds **15** (1 g) and **16** (0.8 g). The residue of fraction C7 was subjected to silica gel column chromatography eluting with a gradient of petroleum ether–EtOAc (100:15, 100:30, 0:100) to yield seven subfractions, C7-1–C7-7. C7-7 was further separated by silica gel CC and purified on Sephadex LH-20 eluting with $CHCl_3$–MeOH (6:4) to afford compound **45** (17 mg). Fraction C10 was then subjected to silica gel CC eluting with a gradient of $CHCl_3$–MeOH (1:40 to 1:30) to provide three subfractions, C10-1–C10-3. C10-1 was further separated by silica gel CC and purified on Sephadex LH-20 eluting with $CHCl_3$–MeOH (1:1) to afford compound **13** (12 mg). C10-3 was chromatographed over silica gel with petroleum $CHCl_3$–MeOH (1:30) and further separated by Sephadex LH-20 ($CHCl_3$–MeOH, 1:1) to obtain compound **41** (31 mg). Fraction C11 was subjected to silica gel CC eluting with a gradient of $CHCl_3$–MeOH (1:30 to 1:35) to yield four subfractions, C11-1–C11-4. C11-1 was subjected to chromatography on silica gel CC eluting with petroleum ether–Me_2CO (4:1) to produce seven subfractions, C11-1-1–C11-1-7. C11-1-3 was further separated by silica gel column chromatography using $CHCl_3$–EtOAc (9:1) to yield four subfractions, C11-1-3-1–C11-1-3-4. C11-1-3-3

was then separated by Sephadex LH-20 ($CHCl_3$–MeOH, 1:1) and semi-preparative HPLC eluted with a gradient of MeOH–H_2O to yield compound **21** (21 mg). C11-1-4 was separated on silica gel CC eluting with $CHCl_3$–EtOAc (9:1) and further purified by Sephadex LH-20 ($CHCl_3$–MeOH, 6:4) to afford compound **18** (33 mg). C11-1-5 was chromatographed over silica gel with petroleum ether–Me_2CO (2.5:1) as eluent, and subfraction was then separated by Sephadex LH-20 ($CHCl_3$–MeOH, 6:4) and semi-preparative HPLC eluted with a gradient of MeOH–H_2O to yield compound **17** (6.3 mg). C11-3 yielded a colorless needle crystal, collected by filtering, and further purified by washing with $CHCl_3$ to afford compound **39** (33 mg). The residue of C11-3 was subjected to silica gel CC eluting with a gradient of $CHCl_3$–MeOH to yield three subfractions, C11-3-1–C11-3-3. C11-3-2 was subjected to silica gel CC eluting with petroleum ether–Me_2CO (4.5:1) to yield compound **46** (6 mg). C11-3-3 was also chromatographed on silica gel with petroleum ether–Me_2CO (4.5:1) as eluent to obtain compounds **34** (7.2 mg) and **35** (11mg). Fraction C12 was chromatographed on silica gel with a gradient of $CHCl_3$–MeOH to give four fractions, C12-1–C12-4. C12-3 was further divided into five subfractions, C12-3-1–C12-3-5, with repeated silica gel CC eluting with $CHCl_3$–MeOH (80:1 to 15:1). Chromatography of C12-3-1 on silica gel with $CHCl_3$–EtOAc (5:1, 4:1) and petroleum ether–Me_2CO (2:1) as eluent successively and purified by semi-preparative HPLC eluting with a gradient of MeOH–H_2O to yield compound **25** (7.8 mg). C12-3-3 was chromatographed over silica gel with $CHCl_3$–EtOAc (4:1, 1:1) as eluent, and the subfraction was then separated by Sephadex LH-20 ($CHCl_3$–MeOH, 6:4) and semi-preparative HPLC eluted with a gradient of MeOH–H_2O to yield compound **44** (21 mg).C12-4 was separated on silica gel CC eluting with $CHCl_3$–MeOH (70:1 to 15:1) and further purified by Sephadex LH-20 eluting with ($CHCl_3$–MeOH, 1:1) to afford compound **47** (28 mg). Fraction C13 was chromatographed with a gradient of $CHCl_3$–MeOH (35:1, 20:1, 5:1) as eluent to give six subfractions, C13-1–C13-6. C13-6 was then subjected to silica gel CC with petroleum ether–Me_2CO (30:1) to obtain five subfractions, C13-6-1–C13-6-5, and subfraction C13-6-4 was further separated by Sephadex LH-20 (petroleum ether–$CHCl_3$–MeOH, 2:1:1) and semi-preparative HPLC with a gradient of MeOH–H_2O as the mobile phase to yield compounds **20** (2.7 mg) and **23** (34 mg). Fraction C14 was subjected to silica gel CC eluting with a gradient of $CHCl_3$–Me_2CO (25:1 to 10:1) to yield five subfractions, C14-1–C14-5. C14-4 was chromatographed over silica gel with $CHCl_3$–Me_2CO (10:1 to 5:1) and further separated by Sephadex LH-20 (petroleum ether–$CHCl_3$–MeOH, 2:1:1) to obtain compound **22** (18 mg). C14-5 was subjected to silica gel CC eluting with $CHCl_3$–Me_2CO (10:1 to 5:1) to give seven subfractions, C14-5-1–C14-5-7. C14-5-5 was purified by Sephadex LH-20 eluting with petroleum ether–$CHCl_3$–MeOH (2:1:1) and semi-preparative HPLC with a gradient of MeOH–H_2O as eluent to afford compounds **38** (6.8 mg) and **19** (9.8 mg). C14-5-7 was further purified by Sephadex LH-20 (petroleum ether–$CHCl_3$–MeOH, 2:1:1) to obtain compound **24** (21 mg). Fraction C15 was subjected to silica gel CC eluting with a gradient of $CHCl_3$–MeOH (25:1 to 10:1) and further separated by chromatographed over silica gel with $CHCl_3$–Me_2CO (5:1) and Sephadex LH-20 ($CHCl_3$–MeOH, 6:4) to obtain compound **37** (69 mg).

The EtOAc layer (50 g) was fractionated on silica gel CC eluting with a gradient of $CHCl_3$–MeOH (10:1 to 0:1) to yield 10 fractions, E1–E10. Fraction E3 was separated by silica gel CC with $CHCl_3$–MeOH–H_2O (16:1:0.1) to give compound **36** (2.5 mg). Fraction E5 was subjected to silica gel CC eluting with $CHCl_3$–MeOH–H_2O (15:1:0.1, 10:1:0.1) to yield E5-1–E5-4, and E5-4 was separated on Sephadex LH-20 eluting with MeOH to yield compound **33** (8 mg). E5-5 was further purified by semi-preparative HPLC with MeOH-H_2O as eluent to obtain compound **40** (8.2 mg). Fraction E7 yielded a yellow powder when standing overnight, and then the yellow powder was further purified to obtain compound **26** (62 mg). The residue of Fraction E7 was chromatographed on silica gel CC eluting with $CHCl_3$–MeOH–H_2O (9:1:0.1, 8:1:0.1, 5:1:0.1), and separated by semi-preparative HPLC with a gradient of MeOH–H_2O as eluent to afford compounds **27** (6.7 mg), **28** (7 mg), and **29** (41 mg). Fraction E8 was separated on silica gel CC eluting with $CHCl_3$–MeOH–H_2O (8:1:0.1, 5:1:0.1) to give nine subfractions, E8-1–E8-9, and then E8-7 was further fractionated on silica gel CC (CH_2Cl_2–isopropanol–H_2O, 4:1:0.05 to 2:1:0.05) and purified by Sephadex LH-20 with MeOH as eluent to yield compound **32** (13 mg).

The *n*-BuOH layer (400 g) was fractionated on silica gel CC eluting with a gradient of $CHCl_3$–MeOH (7:1 to 0:1) to yield 14 fractions, B1–B14. Fraction B5 yielded a white powder, collected by

filtering, which was then purified by washing with MeOH to obtain compound 43 (120mg). Fraction B6 was further separated on silica gel CC eluting with $CHCl_3$–MeOH–H_2O (5:1:0.1 to 2:1:0.1) to give nine subfractions, B6-1–B6-9. B6-7 was further divided by silica gel CC using $CHCl_3$–MeOH–H_2O (5:1:0.1), and purified by Sephadex LH-20 eluting with MeOH to afford compound **42** (14 mg). Fraction B12 was separated on repeated silica gel CC eluting with $CHCl_3$–MeOH–H_2O (2:1:0.1, 1:1:0.1) and further purified by silica gel CC (CH_2Cl_2–isopropanol–H_2O, 4:1:0.1) and semi-preparative HPLC with MeOH–H_2O as eluent to yield compounds **30** (7 mg) and **31** (6 mg), respectively.

3. Anti-Inflammatory Activity Assay In Vitro

Table S1. Anti-inflammatory activity assay in vitro [a].

Plate	Compound	OD Value [b]	I.R. (%)	Plate	Compound	OD Value [b]	I.R. (%)
	control	0.643 ± 0.064			control	0.559 ± 0.044	
	model	0.923 ± 0.032			model	0.930 ± 0.079	
	Ginkgolide B [c]	0.712 ± 0.015 ***	75.4		Ginkgolide B [c]	0.649 ± 0.028 **	75.7
	Pet. extract [d]	0.861 ± 0.085	22.1	3#	**15**	0.850 ± 0.020	21.6
1#	$CHCl_3$ extract [d]	0.708 ± 0.139 **	76.8		**19**	0.427 ± 0.024 ***	135.6 [e]
	EtOAc extract [d]	0.847 ± 0.099	27.1		**27**	0.832 ± 0.042	26.4
	n-BuOH extrac t [d]	0.867 ± 0.076	20.0		**29**	0.788 ± 0.044 *	38.3
	**14**	0.808 ± 0.069 ***	41.1		**41**	0.806 ± 0.013 *	33.4
	**17**	0.845 ± 0.029 **	27.9		control	0.710 ± 0.033	
	**18**	0.819 ± 0.023 **	37.1		model	1.046 ± 0.115	
	control	0.619 ± 0.034			Ginkgolide B [c]	0.774 ± 0.025 **	80.9
	Model	0.861 ± 0.053			**2**	0.915 ± 0.051	39.0
	Ginkgolide B [c]	0.706 ± 0.015 **	64.1		**4**	0.811 ± 0.014 **	69.9
	**1**	0.763 ± 0.048 *	40.5	4#	**22**	0.879 ± 0.052 *	49.7
	**5**	0.799 ± 0.055	25.6		**32**	0.903 ± 0.041 *	42.6
	**7**	0.721 ± 0.075 **	57.9		**33**	0.897 ± 0.010 *	44.4
	**8**	0.726 ± 0.038 **	55.8		**40**	0.802 ± 0.055 **	72.6
2#	**23**	0.795 ± 0.024	27.3		**43**	0.821 ± 0.096 **	66.9
	**24**	0.777 ± 0.053 *	34.7		**45**	0.855 ± 0.042 **	56.9
	**25**	0.737 ± 0.067 *	51.2				
	**30**	0.773 ± 0.064 *	36.4				
	**42**	0.786 ± 0.014 *	31.0				
	**44**	0.796 ± 0.142	26.9				
	**46**	0.693 ± 0.039 **	69.4				

[a] All samples were assigned to five different 96-well plates, 1#–4#; The Student's *t*-test for unpaired observations between model (stimulated by PAF alone) and control (cultured in medium alone) or tested samples was carried out to identify statistical differences; * $p < 0.05$, ** $p < 0.01$, *** $p < 0.001$; $p < 0.05$ were considered as significantly different; All the compounds were tested at 10^{-5} mol/L. [b] OD values were expressed as mean ± SD (for control and sample, $n = 3$; for model, $n = 4$). [c] Ginkgolide B, positive control. [d] Pet. extract, $CHCl_3$ extract, EtOAc extract, and n-BuOH extract represent the extract described in "*Extraction and Isolation*" (tested at 10 μg/mL). [e] The tests were repeated several times, and the results were reproducible.
